# Differences in psychologists’ cognitive traits are associated with scientific divides

**DOI:** 10.1038/s41562-025-02153-1

**Published:** 2025-04-17

**Authors:** Justin Sulik, Nakwon Rim, Elizabeth Pontikes, James Evans, Gary Lupyan

**Affiliations:** 1 Cognition, Values and Behavior Lab, Munich Interactive Intelligence Initiative, LMU Munich, Munich, Germany; 2 Department of Psychology, University of Chicago, Chicago, IL USA; 3 Graduate School of Management, University of California-Davis, Davis, CA USA; 4 Department of Sociology and Department of Data Science, University of Chicago, Chicago, IL USA; 5 Santa Fe Institute, Santa Fe, NM USA; 6 Paradigms of Intelligence, Google Research, Mountain View, CA USA; 7 Department of Psychology, University of Wisconsin-Madison, Madison, WI USA

**Keywords:** Human behaviour, Science, technology and society, Psychology

## Abstract

Scientific research is often characterized by schools of thought. We investigate whether these divisions are associated with differences in researchers’ cognitive traits such as tolerance for ambiguity. These differences may guide researchers to prefer different problems, tackle identical problems in different ways, and even reach different conclusions when studying the same problems in the same way. We surveyed 7,973 researchers in psychological sciences and investigated links between what they research, their stances on open questions in the field, and their cognitive traits and dispositions. Our results show that researchers’ stances on scientific questions are associated with what they research and with their cognitive traits. Further, these associations are detectable in their publication histories. These findings support the idea that divisions in scientific fields reflect differences in the researchers themselves, hinting that some divisions may be more difficult to bridge than suggested by a traditional view of data-driven scientific consensus.

## Abstract (web summary)

Scientific disagreements are not just a matter of using different methods or having conflicting data. Sulik et al. surveyed psychological scientists and found that disagreements are also associated with differences in researchers’ cognitive traits.

## Main

The view of science as the objective pursuit of knowledge suggests that when scientists are faced with the same data, they should reach the same conclusions. On this view, disagreements between scientists are driven by differences in what they know. As data build up and knowledge gaps diminish, so should disagreements. In many cases, this normative (pro-epistemic^1^) view is justified. Certain ideas, clearly disconfirmed by data, have all but vanished. For example, the Ptolemaic Earth-centric view was displaced by Copernican heliocentrism. Lamarck’s hypothesis of inherited characteristics was largely discarded by the scientific community as evidence mounted in favour of Darwin’s theory of natural selection^2^. Phlogiston, the luminiferous aether and caloric fluid are all consigned to the past.

While some disagreements are resolved by data that unambiguously support one side or another, many controversies persist and become entrenched schools of thought. For example, social sciences are divided by factions that favour social constructionism vs biological essentialism^3–6^, dispositional versus situational explanations for human behaviour^7,8^, and biological reductionism versus theories that emphasize social and cultural causal autonomy^9,10^.

The clustered nature of such differences suggests that fields such as psychology are fractured into distinct cultures^11^. But such divisions are not unique to psychology or the social sciences. Theoretical physics is split over whether string theory or loop quantum gravity best unifies inconsistencies in quantum theory and gravitational fields^12,13^. In biology, researchers can agree on the data while still producing substantially different explanations of it^14^. Even Lamarck’s ideas are not completely dormant, with some researchers suggesting that a Lamarckian lens can inform recent discoveries in epigenetics^2,15,16^.

What factors determine whether researchers align themselves with one school of thought or another? One possibility is that researchers across scientific divisions do not, in fact, know the same things. A researcher who is most familiar with data supporting a particular school of thought will naturally align themselves with it. A related factor—one that can help explain how such differential knowledge can arise—is educational and professional networks^17,18^. A graduate student finding themselves in a lab that uses a specific method or espouses a specific theory will become an expert in that method or theory and its associated literature while being less knowledgeable of alternative approaches.

It is also possible that academic disagreements persist due to individual differences not directly related to the research itself. For instance, a previous study observed sincere differences of opinion about the inferential value of different research methods for controversial topics in psychology and sought to explain those differences by appeal to political ideology^6^. However, this approach produced mostly null results.

Another possibility is that disagreements stem in part from differences in researchers’ cognitive traits causing them to find certain types of approaches, findings and theories more or less compelling. Indeed, scientists inhabiting various factions often differ substantially in their theoretical positions despite using the same research methods and being aware of the same empirical data^19^. Differences in cognitive dispositions may draw scientists to pursue certain approaches in the first place. After all, most of the time, a graduate student does not simply find themselves in a certain lab—they actively choose it. It is possible that these choices are made in part because of a match between an approach or philosophy favoured by the student and that practiced in the lab, unintentionally reinforcing existing fractures. If some common cognitive dispositions contribute to the formation of research clusters, it would be unsurprising that schools of thought are both ubiquitous and persistent.

The idea that researchers’ work is impacted by their psychological traits—in tandem with their education and social contexts—has a long history in the psychology of science^20–22^. In particular, science is a kind of problem solving^23,24^, and cognitive traits predict differences in problem solving from hypothesis generation to hypothesis testing^25–27^. Cognitive traits have been linked to what field someone chooses: higher systematizing quotients have been associated with people who pursue physical sciences while higher empathizing quotients has been associated with pursuing the humanities^28^. Scientists report greater spatial visual imagery than humanities researchers; the latter report higher object visual imagery^29,30^. Differences in some personality dimensions has also been found to predict endorsement of mechanistic/objectivist vs organismic/holistic worldviews across fields^31^.

Work examining these kinds of associations among researchers within a field is rarer. Within psychology, researchers who use more qualitative approaches have been found to favour more holistic explanations, while those who use more quantitative approaches favour more analytic explanations^32^. Differences along particular psychological dimensions have also been linked to specific disagreements in specialist subfields. For example, cognitive scientists’ stance in the imagery debate (whether visual imagery plays a constitutive or largely epiphenomenal role^33,34^) has been linked to the vividness of the scientists’ own visual imagery^35,36^.

The studies just mentioned have either focused on broad differences across sciences and humanities without explaining persistent disagreements between people who study similar things in similar ways, or else concern smaller-scale disagreements within a highly specialized topic. Neither helps us in explaining how a broad field might fracture into schools of thought. Importantly, the existing work also has not controlled for differences in research methods and topics of study, making it difficult to infer the role of a researcher’s cognitive dispositions.

## Study overview

To better understand the associations between scientific divisions and differences in cognitive dispositions, even among researchers studying the same topics, we conducted a large-scale survey of researchers in psychology and allied disciplines such as cognitive neuroscience.

**Extended Data Fig. 1:**
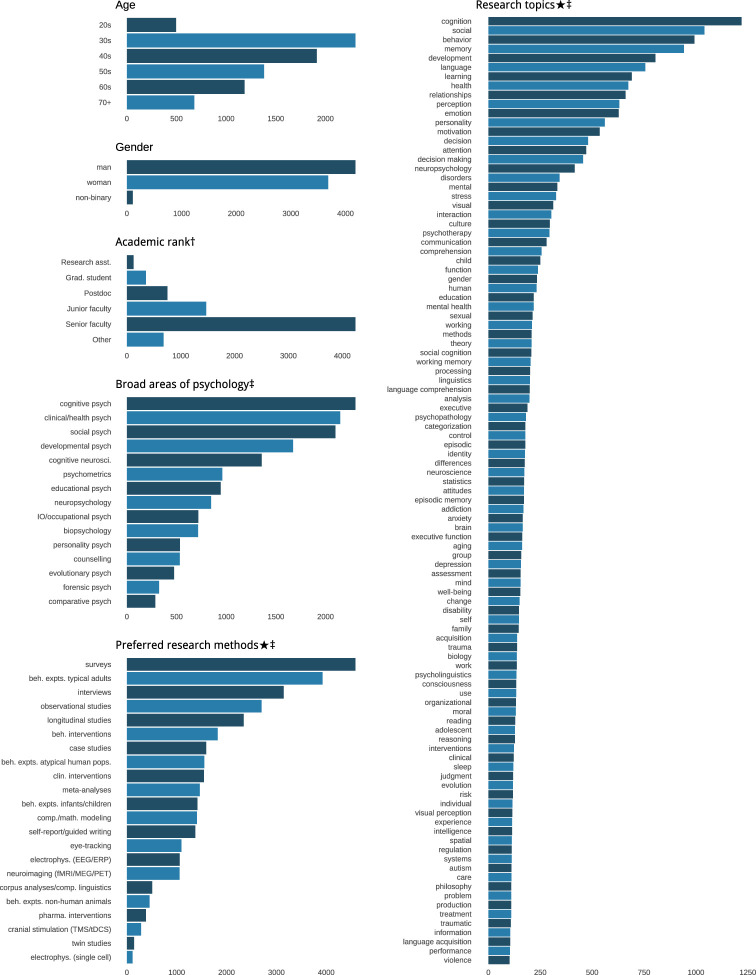
Histograms of demographic and research variables. † excluding respondents not in academia; ‡ respondents could choose more than one category; ⋆ plot truncated to show only counts > 100.

We recruited academic researchers to complete a survey that probed their stance on controversial themes in psychology (Extended Data Table 1). The survey also included several validated scales measuring individual differences in cognitive traits. To understand academics’ specific research backgrounds, we asked which areas of psychology they identified with, which topics they studied and which common research methods or tools they typically used. These classes of survey variable (controversial themes; cognitive measures; research areas, topics and methods) are summarized in Table 1, with further details provided in Methods, as well as Extended Data Fig. 1 and Extended Data Table 1. Respondents also provided demographic information such as their gender, age and academic position (graduate student, junior faculty and so on).

**Table 1:** Overview of main survey variables

Variable class	Example construct	Example item/response
Controversial themes	Social environment	Most human behaviours cannot be productively studied without reference to people’s social environment.
	Thinking ~ language	Without language, human cognition would be unrecognizable.
	Neurobiology essential	The key to understanding human behaviour is to understand its (neuro)biological mechanisms.
Cognitive measures	Tolerance of ambiguity	I have always felt there is a clear difference between right and wrong.
	Visual imagery (spatial)	I can easily imagine and mentally rotate three-dimensional geometric figures.
	Cognitive structure	I want things to proceed according to plan.
Research areas	-	Cognitive psychology
	-	Clinical psychology
	-	Social psychology
Research topics	-	Memory
	-	Language
	-	Relationships
Research methods	-	Surveys
	-	Behavioural experiments with typical adults
	-	Interviews

For more details and the remaining constructs, see Methods, Extended Data Fig. 1 and Extended Data Table 1.

To evaluate whether differences in researchers’ stances and cognitive traits are associated with differences in actual scientific output, we also asked respondents for their consent to anonymously link their survey responses with their publication records in the Web of Science and Microsoft Academic Graph. We applied machine learning techniques to these data to build a citation model (indicating what literature the researchers draw on), a semantic model based on published abstracts and titles (indicating what sort of work they publish), and a co-authorship model (indicating potential paths of mutual influence).

Using these data, we sought to understand whether differences in cognitive traits/dispositions among psychology researchers are associated with their stances on various controversial themes; whether these associations remain when controlling for research areas, methods and topics; and whether these various patterns of associations are detectable in published scientific outputs. If scientific schools of thought are associated with interpersonal differences, they may be more deeply entrenched and more difficult to bridge than the traditional view of science suggests.

## Results

### Descriptive overview

Extended Data Fig. 1 shows an overview of respondents’ (*n* = 7,973) demographics and research interests (also see Methods for demographics). The modal rank was ‘senior faculty’. The three most common areas of study among respondents were cognitive psychology (2,301), clinical/abnormal/health psychology (2,148) and social psychology (2,100); the three most common research methods were surveys (4,586), behavioural experiments with typical adults (3,927) and interviews (3,146). For topics of study, we excluded keywords entered by 10 or fewer respondents and grouped rarer topics with more common ones (for example, respondents who studied ‘group processes’ or ‘group relations’ both appear under ‘group’ in Extended Data Fig. 1). Responses covered a diverse range of topics. For instance, Extended Data Fig. 1 shows that the topics ‘parenting’, ‘eating’, ‘speech’, ‘leadership’ and ‘media’ include almost a hundred participants each. Although we cannot be certain that our sample is representative of academic psychology, our sample covers a wide range of research areas, topics, methods and demographic groups.

### Responses to controversial themes

**Fig. 1:**
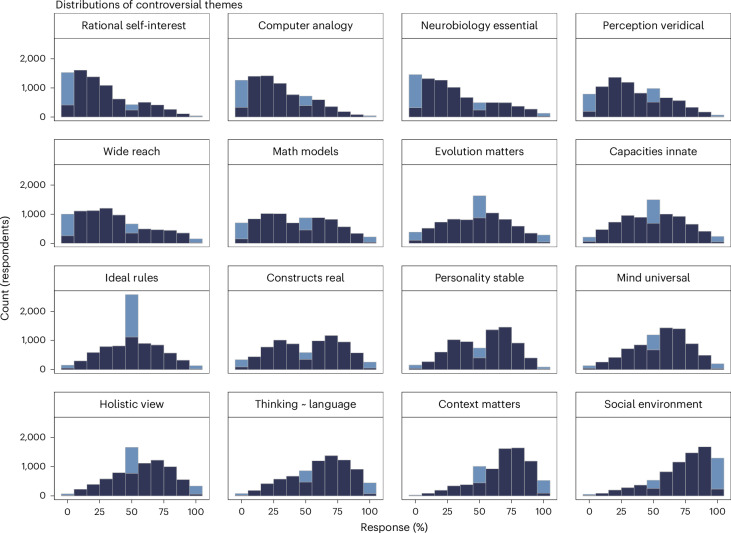
Histogram of responses to controversial themes. Responses shown on a percentage scale where 100% represents complete agreement with the upper anchor label in Extended Data Table 1 and 0% represents complete agreement with the lower anchor label. Three distinctive response patterns are highlighted in lighter colour: extreme responses whereby participants moved the response slider all the way to 0 or 100%; and responses of exactly 50%, which necessitated moving the slider off the midpoint where it was initially, and then purposefully moving it back onto the midpoint (leaving it untouched on the midpoint would have prevented them from continuing to the next trial). Thus, for instance, the middle bin for theme ‘ideal rules’ contains some responses precisely at 50% in lighter blue and some responses near 50% in darker blue. Similarly, the left-most bin for theme ‘rational self-interests’ contains some responses exactly at 0% in light blue and some responses near 0% in dark blue. We highlight these distinct response strategies to emphasize the connection with regression models that quantify bimodality, extreme responses and midpoint spikes in Supplementary Table 1.

**Extended Data Fig. 2:**
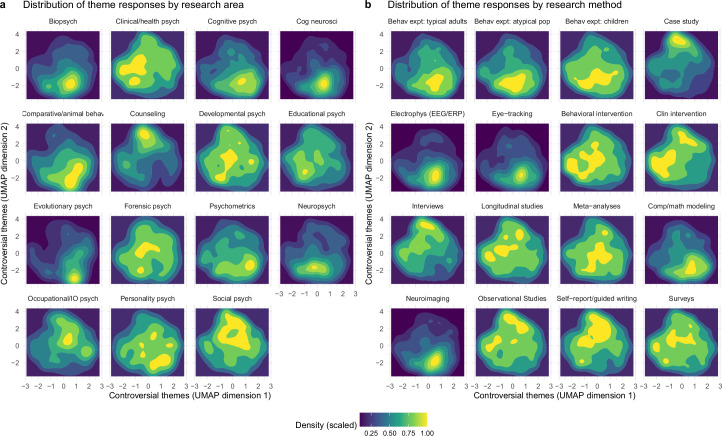
Distributions of research areas and methods projected onto a 2D UMAP representation of controversial themes. Contour plots providing a high-level overview of how responses to controversial themes were distributed, projecting responses to all 16 themes onto a 2D space (UMAP parameters: number of nearest neighbors = 15; minimum distance = 0.1). Plot panels show distributions for (**a**) each research area; and (**b**) the most common research methods. These illustrate, for instance, how some research areas (for example, cognitive neuroscience, biopsychology) show greater concentration within this space than others (for example, social and developmental psychology are rather more dispersed). Shading represents density of the research area/methods responses associated with each participant in 2D space, not the controversial themes originally input into the UMAP algorithm. Density is scaled, such that the peak density in each sub-panel has a value of 1.

For all 16 themes (described in Extended Data Table 1), some respondents endorsed positions at both extremes (Fig. 1). Some themes showed a degree of consensus. For example, most respondents thought the ‘Homo economicus’ conception of human self-interested rationality is a poor model for human behaviour (‘rational self-interest’ mean response = 27.7, s.d. = 24.3); and they agreed that human behaviour should be studied with reference to people’s social environment (‘social environment’ mean response = 74.1, s.d. = 22.4). Other themes showed a bimodal distribution, indicating more substantial disagreement. Respondents were split on whether the things studied by psychologists such as short-term memory really exist or are just theoretical constructs (‘constructs real’) and whether personality is largely stable across the lifespan (‘personality stable’). Finally, several themes showed less-decisive responses. The large spike at the midpoint of ‘ideal rules’ suggests that many respondents where not sure what to think when asked whether psychology should focus on discovering general rules that govern cognition or studying the ways in which cognition departs from such ideal rules. Supplementary Table 1 presents regression models that quantify bimodality and peaks such as the spike at the midpoint of ‘ideal rules’. Furthermore, Extended Data Fig. 2 provides a simultaneous overview across all 16 controversial themes by projecting these onto a two-dimensional space using uniform manifold approximation and projection (UMAP).

### Predictors of respondents’ stances on controversial themes

**Fig. 2:**
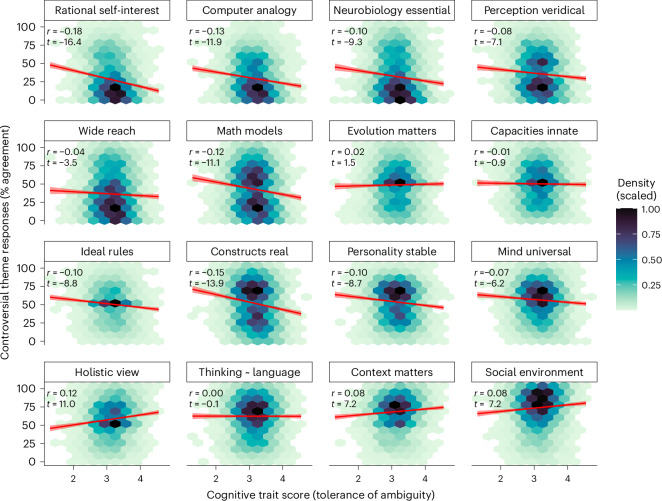
Controversial themes regressed on cognitive trait ‘tolerance of ambiguity’. Illustrative plots of the linear relationships between each controversial theme and one of the cognitive traits: tolerance of ambiguity. Hexes show binned 2D density plots (with density scaled such that the peak density in each subpanel has a value of 1). Linear fits are shown in red (mean predictions with 99.9% CI ribbons). The correlation coefficient (Pearson’s *r*) corresponding to each fit is annotated in the top left of each panel, along with a *t*-value (for a full table of numeric results, see Supplementary Table 4). The OSF repository (https://osf.io/zyec9/) contains additional analyses, including non-parametric (Spearman’s *r*) models of the above, illustrating how these associations do not depend on an assumption of linearity. The OSF repository also contains similar plots for the other controversial themes, along with density plots for the binary variables such as research areas and methods.

**Extended Data Fig. 3:**
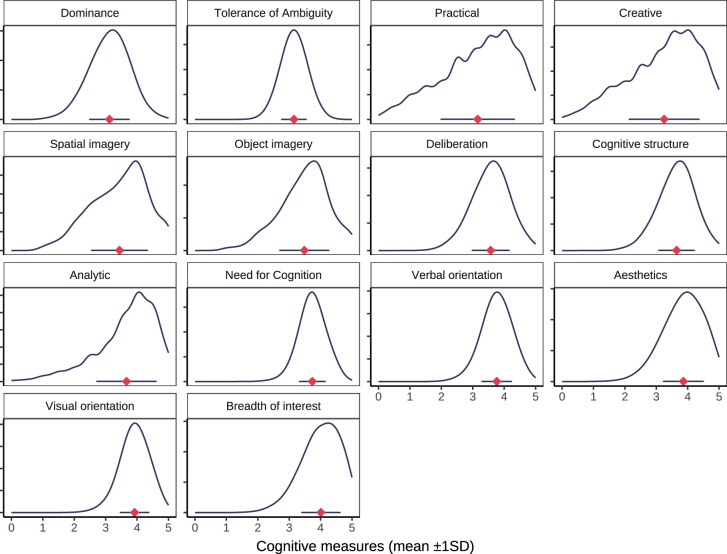
Distributions of cognitive trait variables. Means ± 1SD (SD whiskers reflecting n=7973 except for Need for Cognition where n=6005).

We began by regressing respondents’ stances regarding the controversial themes on their cognitive traits (see distributions in Extended Data Fig. 3), areas of psychology, research methods and gender (focusing here on binary genders as including non-binary respondents as a predictor category did not reach the threshold for significance, corrected for multiple comparisons). To illustrate, Fig. 2 plots the linear relationships between all 16 themes and one of the cognitive traits (tolerance of ambiguity). Linear relationships for the other cognitive traits are summarized in Appendix 2 (extended regression outputs). The OSF repository (https://osf.io/zyec9/) includes additional plots and analyses using non-parametric rank models to show that associations between controversial themes and cognitive traits do not depend heavily on an assumption of linearity.

**Fig. 3:**
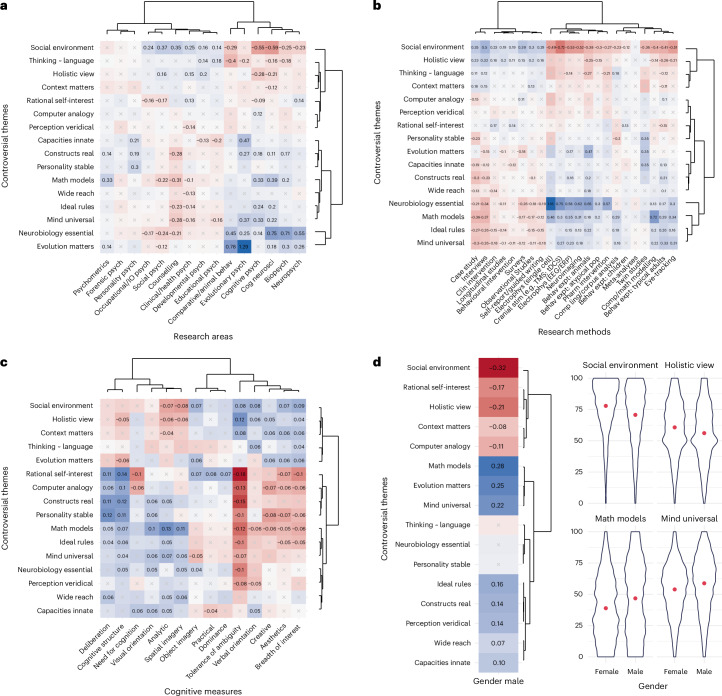
Regression coefficients for controversial themes. **a**–**d**, Regression coefficients for controversial themes as a function of research areas, (**b**) research methods (**b**), cognitive traits (**c**) and gender (**d**). For tables of full numeric results, see Supplementary Tables 2–5. Cells marked ‘x’ are non-significant (with Bonferroni correction for multiple comparisons—the number of cells in each panel—yielding thresholds *P* < 0.000208 for **a**, *P* < 0.000142 for **b** and *P* < 0.000223 for **c**). All continuous variables are *z*-scored. Plot margins show hierarchical clusters (Ward’s method). **d**, In place of clusters for gender, given the low dimensionality of the space representing gender, two themes are shown where men gave lower scores than women and two with the reverse pattern (violin plots show full response distributions, with group means in red). For further distribution plots including non-binary participants, see the OSF repository at https://osf.io/zyec9/.

The associations between the controversial themes and other variables (cognitive traits, areas of psychology, research methods and gender) are summarized in Fig. 3. For instance, the fit parameters from Fig. 2 form one column labelled ‘tolerance of ambiguity’ in Fig. 3c.

The ordering of variables within a given panel reflects hierarchical clusters derived from the coefficients within that panel’s cells^37^. For example, in Fig. 3a, controversial themes from ‘social environment’ to ‘perception veridical’ in the top half of the panel all appear in the same broad cluster because they have similar associations with the various research areas. Conversely, research areas from ‘comparative/animal behaviour’ to ‘neuropsychology’ in the right half of the panel are clustered together because they have similar associations with the various controversial themes. Correspondingly, the clusters of controversial themes in Fig. 3b are defined by themes having similar associations across the various research methods, whereas the clusters of themes in Fig. 3c are defined by associations with cognitive traits.

Note that although certain cells in Fig. 3 are marked with an ‘x’ to indicate that those individual associations did not reach the Bonferroni-adjusted significance threshold (all *P* values reported here are two-tailed), the clusters (as well as subsequent regression models below) are evaluated using all cell values.

#### Areas of psychology and research methods

Individual cells in Fig. 3a,b show many of the relationships one would expect if our measures have construct validity. For example, researchers in evolutionary and comparative psychology thought that psychological theories should focus more on the evolution of human mental faculties (evolutionary psych: *β* = 1.293, bootstrapped 95% CIs [1.205, 1.381], *t* = 28.703, *P* < 0.001; comparative psych: *β* = 0.778 [0.661, 0.895], *t* = 13.058, *P* < 0.001). Cognitive neuroscientists thought that (neuro-)biological mechanisms are essential for explaining human behaviour (*β* = 0.753 [0.697, 0.809], *t* = 26.333, *P* < 0.001), as did biopsychologists (*β* = 0.712 [0.637, 0.787], *t* = 18.570, *P* < 0.001) and neuropsychologists (*β* = 0.551 [0.480, 0.621], *t* = 15.369, *P* < 0.001).

The importance of (neuro-)biological explanations was higher among respondents who reported using electrophysiological methods (single cell *β* = 1.163 [0.980, 1.345], *t* = 12.497, *P* < 0.001; EEG *β* = 0.580 [0.517, 0.643], *t* = 17.933, *P* < 0.001), cranial stimulation (*β* = 0.748 [0.630, 0.865], *t* = 12.496, *P* < 0.001), neuroimaging (*β* = 0.622 [0.559, 0.686], *t* = 19.288, *P* < 0.001) or behavioural experiments with animals (*β* = 0.651 [0.558, 0.745], *t* = 13.646, *P* < 0.001). Those who reported using mathematical models in their research also indicated that mathematical models are more important to scientific progress in psychology (*β* = 0.723 [0.668, 0.778], *t* = 25.595, *P* < 0.001).

More importantly, the data also reveal less obvious associations between beliefs, areas of study and methods. For instance, researchers who use physiological and neuroimaging/stimulation methods reported lower belief in the importance of social environment (cranial stimulation: *β* = −0.720 [−0.838, −0.603], *t* = −12.031, *P* < 0.001; neuroimaging: *β* = −0.515 [−0.579, −0.452], *t* = −15.856, *P* < 0.001; single-cell electrophysiology: *β* = −0.492 [−0.676, −0.308], *t* = −5.25, *P* < 0.001; EEG: *β* = −0.527 [−0.590, −0.463], *t* = −16.227, *P* < 0.001). While there may be practical limitations to the number of interacting people that can be studied simultaneously with such methods, this need not go together with a theoretical belief about the non-importance of social context in explaining human cognition, yet our data show just such a relationship.

These patterns of association are quite extensive (and can be explored in more detail with our data dashboard linked in OSF at https://osf.io/zyec9/). A controversial theme may be associated with multiple methods, or a method may be associated with responses to multiple themes. For instance, social environment was also deemed less important among respondents who reported using pharmacological interventions (*β* = −0.270 [−0.373, −0.168], *t* = −5.165, *P* < 0.001), eye tracking (*β* = −0.508 [−0.571, −0.445], *t* = −15.857, *P* < 0.001), mathematical modelling (*β* = −0.398 [−0.455, −0.341], *t* = −13.686, *P* < 0.001) and behavioural experiments with various populations (typical adults: *β* = −0.413 [−0.456, −0.370], *t* = −18.863, *P* < 0.001; children: *β* = −0.123 [−0.180, −0.065], *t* = −4.188, *P* < 0.001; atypical populations: *β* = −0.300 [−0.356, −0.245], *t* = −10.701, *P* < 0.001; animals: *β* = −0.375 [−0.470, −0.281], *t* = −7.805, *P* < 0.001). Conversely, those preferring case studies showed exactly the opposite theoretical commitments for 12 out of the 16 themes compared with those preferring behavioural experiments (Fig. 3b). A range of methods—from case studies to self-reports—were associated with similar stances on multiple beliefs, including the value of certain explanations (neurobiology essential, math models), whether psychology should aim at uncovering rules governing cognition (ideal rules) or how widely individual minds differ (mind universal).

#### Cognitive traits

That one’s choice of research area and research method is associated with how one thinks about research is not especially surprising and need not indicate a causal relationship. A researcher who, for whatever reason, finds explanations that stress social context unpalatable may gravitate toward methods and research questions that focus on the individual (or specific component systems) as units of analysis. It is more surprising if one’s stance on scientific questions is associated with putatively stable^38^ cognitive or dispositional traits (Fig. 3c).

The trait associated with the broadest range of controversial themes was tolerance of ambiguity^39,40^. Respondents who reported being more tolerant of ambiguity were less likely to endorse a ‘Homo economicus’ view of behaviour (rational self-interest *β* = −0.181 [−0.202, −0.159], *t* = −16.412, *P* < 0.001), less likely to think that constructs such as working memory really exist (constructs real *β* = −0.154 [−0.176, −0.132], *t* = −13.926, *P* < 0.001), and less willing to see computers as a useful analogy for studying cognition (computer analogy *β* = −0.132 [−0.154, −0.110], *t* = −11.865, *P* < 0.001).

Conversely, ambiguity-tolerant researchers are more likely to say that accounts grounded in single processes (such as working memory) are worse explanations than accounts that consider several such processes in concert (holistic view *β* = 0.122 [0.100, 0.144], *t* = 10.981, *P* < 0.001), more likely to say explanations of human cognition should be sensitive to context (context matters *β* = 0.080 [0.058, 0.102], *t* = 7.155, *P* < 0.001), and more willing to appeal to social environment in explaining behaviour (social environment *β* = 0.080 [0.058, 0.102], *t* = 7.172, *P* < 0.001). Other cognitive traits that patterned similarly to tolerance of ambiguity were verbal orientation and abbreviated scales centering on creativity and breadth of interest.

Another cluster of cognitive traits focusing on cognitive structure and analytic thinking was associated with responses to the controversial themes in a broadly opposite direction to the above ambiguity/creativity cluster. For instance, participants who reported higher cognitive structure (a greater need for logic in their thinking and deliberation in planning in their lives) were more likely to endorse the ‘Homo economicus’ view of behaviour (rational self-interest *β* = 0.136 [0.114, 0.158], *t* = 12.259, *P* < 0.001), or to believe that constructs such as working memory really exist (constructs real *β* = 0.115 [0.093, 0.137], *t* = 10.333, *P* < 0.001), among others.

Interestingly, two aspects of visual imagery belonged to different clusters in Fig. 3c. Spatial imagery patterned more like the analytic or structure cluster, whereas object imagery patterned more like the creativity or ambiguity cluster, although there were exceptions to this pattern: unlike the rest of the creativity/ambiguity cluster, object imagery was associated with higher endorsement of rational self-interest (*β* = 0.073 [0.051, 0.095], *t* = 6.546, *P* < 0.001) and greater interest in grounding explanations of cognition in neurobiology (*β* = 0.044 [0.022, 0.066], *t* = 3.947, *P* < 0.001).

#### Illustrative case studies

**Fig. 4:**
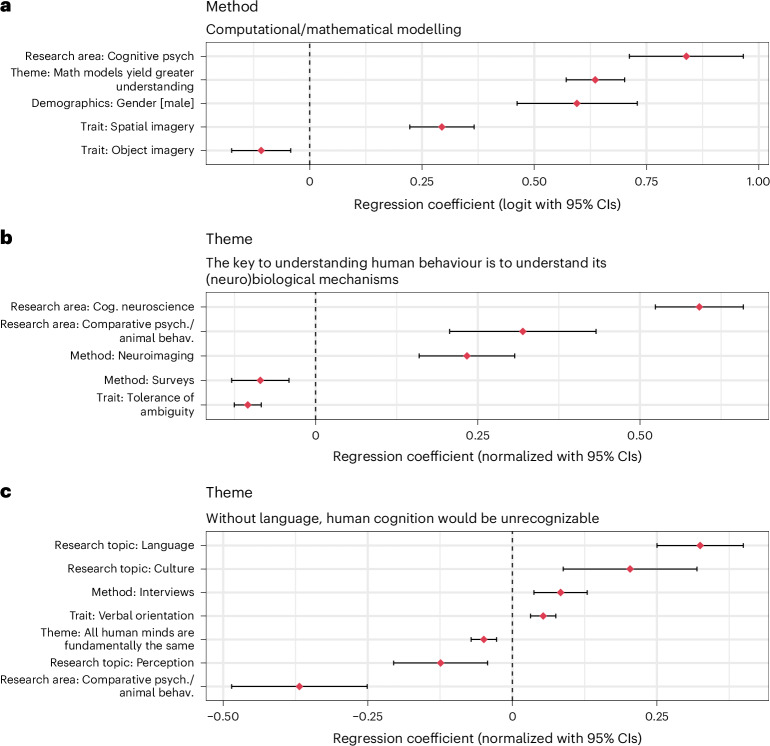
Three illustrative case studies, combining heterogeneous survey variables. **a**–**c**, Regression coefficients predicting three outcome variables. **a**, Whether researchers use computational/mathematical modelling in their work. **b**, Responses to controversial theme ‘neurobiology essential’. **c**, Responses to controversial theme ‘thinking ~ language’. Points show regression coefficients (with 95% CIs, reflecting sample sizes *n* = 7,865 for **a**; *n* = 7,973 for **b** and **c**). When the outcome variable is binary (**a**, ‘method’), the coefficients are expressed in logits. All continuous variables are *z*-scored, yielding standardized coefficients. Note that the *x* axes differ among panels to reflect the range of associations for three different outcomes.

It is instructive to inspect how the patterns shown in Fig. 3 can be used to understand relationships among researchers’ stances on controversial themes, research practices and stances on various controversial themes. Figure 4 shows three examples in which we visualize the relative contribution of various regression predictors to help contextualize the effect sizes (pivoting, in this subsection, to regressions with multiple predictors).

Higher usage of computational/mathematical models (illustrative case A) was associated with being a researcher in cognitive psychology (*β* = 0.839 [0.712, 0.966], *z* = 12.956, *P* < 0.001), being a man (*β* = 0.595 [0.462, 0.729], *z* = 8.715, *P* < 0.001) and endorsement of the importance of mathematical models to scientific progress (*β* = 0.636 [0.571, 0.701], *z* = 19.161, *P* < 0.001). It was also associated with greater spatial imagery (*β* = 0.294 [0.223, 0.366], *z* = 8.052, *P* < 0.001)—an intriguing replication of previous work showing links between spatial imagery and mathematical aptitude^41,42^. By contrast, lower usage of computational/mathematical models was associated with more vivid object imagery (*β* = −0.108 [−0.174, −0.042], *z* = −3.227, *P* = 0.001).

Endorsement of the primacy of biological explanations (a kind of biological reductionism, case B) was associated with being a cognitive neuroscientist (*β* = 0.592 [0.524, 0.659], *t* = 17.102, *P* < 0.001), being a comparative psychologist (*β* = 0.319 [0.207, 0.432], *t* = 5.548, *P* < 0.001) and using neuroimaging methods (*β* = 0.233 [0.160, 0.307], *t* = 6.210, *P* < 0.001). By contrast, lower endorsement of this theme also associated with using survey methods (*β* = −0.085 [−0.129, −0.041], *t* = −3.776, *P* < 0.001) and with tolerance of ambiguity (*β* = −0.105 [−0.125, −0.084], *t* = −9.839, *P* < 0.001).

In case C, endorsement of the centrality of language in human cognition^43^ was positively associated with doing research on language (*β* = 0.325 [0.250, 0.399], *t* = 8.527, *P* < 0.001) and culture (*β* = 0.204 [0.088, 0.319], *t* = 3.452, *P* < 0.001) or using interviews as a method (*β* = 0.083 [0.037, 0.129], *t* = 3.556, *P* < 0.001). It was negatively associated with doing research on perception (*β* = −0.124 [−0.205, −0.043], *t* = −2.992, *P* = 0.003), working in comparative psychology (*β* = −0.368 [−0.485, −0.251], *t* = −6.158, *P* < 0.001) or endorsing the belief that all human minds are fundamentally the same (*β* = −0.049 [−0.071, −0.027], *t* = −4.401, *P* < 0.001). Intriguingly, researchers who themselves are more verbally oriented^44^ were also more likely to report believing that human cognition is more linked to language (*β* = 0.053 [0.032, 0.075], *t* = 4.794, *P* < 0.001).

### Underlying beliefs

There are striking patterns of similarity in how the controversial themes cluster across the various panels in Fig. 3: regardless of whether themes are clustered according to their associations with research areas, methods or cognitive traits, certain themes tend to cluster together. Might these clusters arise from latent factors representing people’s underlying beliefs or worldviews?

**Fig. 5:**
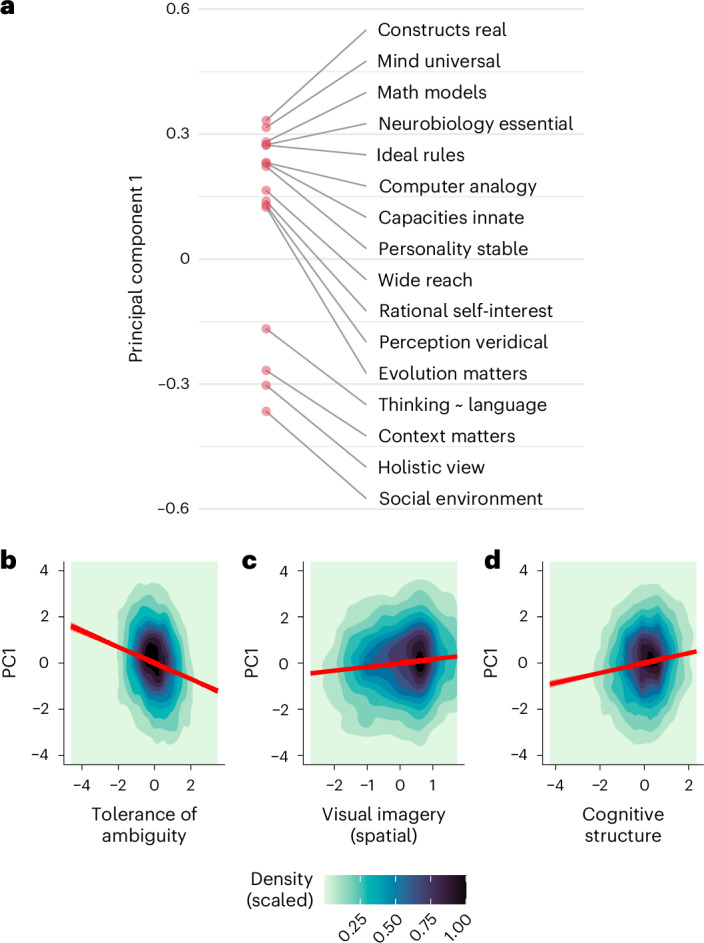
Principal components representation of controversial themes, including associations with cognitive traits. **a**, Themes projected onto the first principal component (PC1). **b**–**d**, Individual differences in cognitive traits are associated with participants’ scores along the first principal component (PC1). Red lines show linear fits, overlaid on 2D density contour plots of the data (density scaled such that the peak density in each subpanel has a value of 1). For further details, see https://osf.io/nkbdr.

For an initial assessment of the plausibility of this idea, we conducted a principal components analysis of responses to the controversial themes. We then extracted the first (most explanatory) component (Fig. 5a) and examined its associations with various traits. Themes with positive loadings on the principal component are concerned with an emphasis on objective reality and quantitative universal explanations. Themes with negative loadings reflect concern for the social environment, context dependence and the importance of taking a more holistic view of human behaviour and cognition.

We projected responses to controversial themes onto the first principal component and regressed these scores on the cognitive traits. Some of the associations between this latent dimension and various individual cognitive traits are shown in Fig. 5b–d: people who were more tolerant of ambiguity scored lower on this latent factor (*β* = −0.212 [−0.233, −0.190], *t* = −19.3, *P* < 0.001); people with more spatial imagery (itself associated with mathematical ability^45^) scored higher (*β* = 0.101 [0.079, 0.123], *t* = 9.07, *P* < 0.001), as did those with greater need for cognitive structure (*β* = 0.132 [0.110, 0.154], *t* = 11.9, *P* < 0.001).

However, a single underlying dimension seems overly reductive. Parallel analysis indicated that the themes contain 5 to 6 latent factors. We conducted an exploratory factor analysis, specifying 5 factors and oblique (geomin) rotation. The model had excellent fit: root mean square error of the approximation (RMSEA) = 0.019, Tucker–Lewis index (TLI) = 0.96, comparative fit index (CFI) = 0.983.

**Fig. 6:**
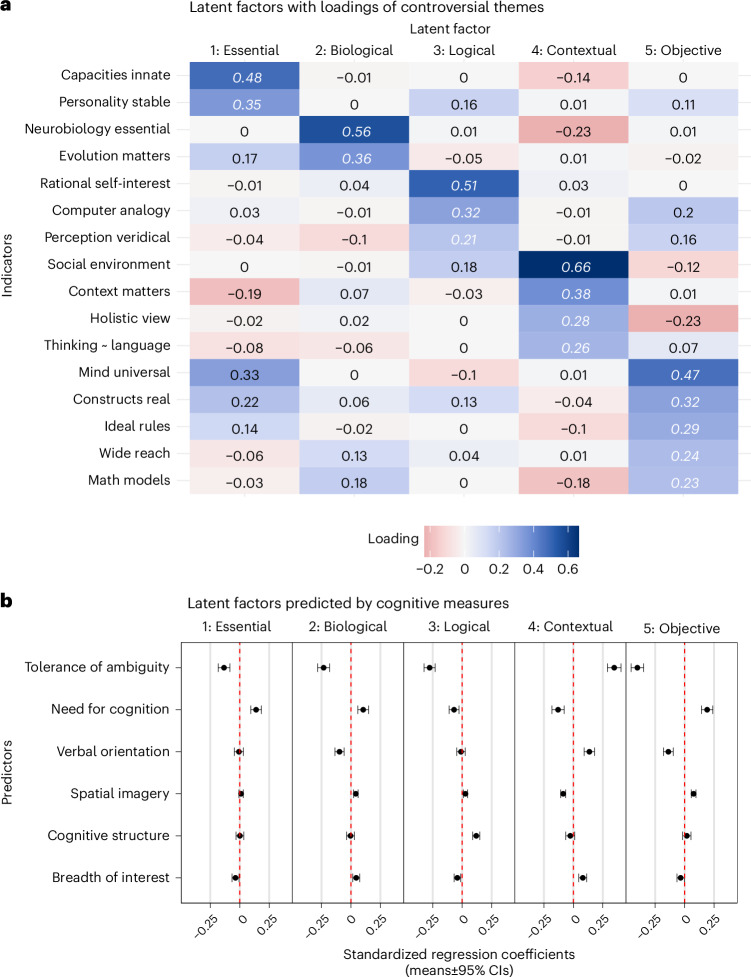
Exploratory factor analysis of controversial themes, including associations with cognitive traits. **a**, Standardized loadings (lambdas) for a 5-factor exploratory factor analysis, representing how individual themes contribute to each latent factor. Cell colour indicates loading direction and strength (bluer, more positive; redder, more negative). Cell texts in white italic font merely highlight which latent factor each theme loads highest on. **b**, Standardized regression coefficients indicating how 6 cognitive traits predict latent factor scores (coefficient with 95% CIs, reflecting *n* = 7,973). Thus, the aforementioned latent factor 1 (‘essential’) is negatively associated with tolerance of ambiguity, positively associated with need for cognition and so on. For a numeric table of regression coefficients, see Supplementary Table 7.

We report standardized loadings (*λ* with 95% CIs) for the two themes most strongly loading on each factor (factor loadings for the rest of the themes are shown in Fig. 6a). Factor 1 (‘essential’) was most strongly associated with themes ‘capacities innate’ (*λ* = 0.478 [0.422, 0.533]) and ‘personality stable’ (*λ* = 0.353 [0.296, 0.409]). Factor 2 (‘biological’) was most strongly associated with themes ‘neurobiology essential’ (*λ* = 0.557 [0.468, 0.646]) and ‘evolution matters’ (*λ* = 0.362 [0.293, 0.432]). Factor 3 (‘logical’) was most strongly associated with themes ‘rational self-interest’ (*λ* = 0.509 [0.435, 0.583]) and ‘computer analogy’ (*λ* = 0.316 [0.263, 0.369]). Factor 4 (‘contextual’) was most strongly associated with themes ‘social environment’ (*λ* = 0.662 [0.534, 0.790]) and ‘context matters’ (*λ* = 0.376 [0.332, 0.420]). Finally, factor 5 (‘objective’) was most strongly associated with themes ‘mind universal’ (*λ* = 0.473 [0.383, 0.563]) and ‘constructs real’ (*λ* = 0.319 [0.253, 0.385]).

To examine associations between these latent factors and individual differences in cognitive traits, we scored each participant along each factor, using factor loadings to weight responses for each theme. The resulting scores for each latent factor were then regressed on select cognitive measures. Figure 6b shows that ‘tolerance of ambiguity’ consistently had strong associations across all latent factors. Participants who were more tolerant of ambiguity were (1) less likely to believe in stable, innate cognitive traits, (2) less likely to endorse evolutionary or neurobiological explanations as the main way to understand human behaviour, (3) less likely to think that human cognition is rational or analogous to symbolic computation, (4) more likely to think that social, contextual or holistic considerations are vital for psychology, and (5) less likely to think that psychological constructs have objective existence or that psychology is about looking for ideal rules governing the human mind. For full numeric results, see Supplementary Table 6.

### Cognitive traits are associated with beliefs, controlling for research

We next sought to understand whether associations between cognitive traits and stances on the controversial themes remain when controlling for researchers’ areas of psychology, research methods and topics. To do so, we pivoted to a higher-dimensional representation of responses. We treated each class of variable—controversial themes, cognitive traits, research areas, topics or methods—as vectors in a respective N-dimensional space (for example, a participant’s responses to the 16 controversial themes are a vector in a 16-dimensional theme space). The cosine similarity between two vectors within a given space represents two participants’ similarity for the variable class represented by that space. Correlations in pairwise cosine similarities across spaces reflect whether participants’ similarity in one space maps onto similarity in another: for example, whether researchers with similar cognitive styles also have similar responses to controversial themes.

**Fig. 7:**
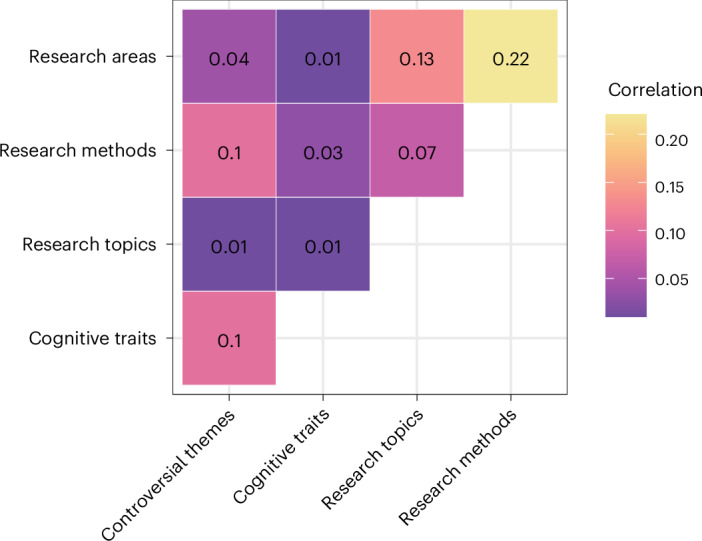
Cosine similarities for the survey responses across abstract spaces representing each class of variable. Correlations between cosine similarities across all pairs of participants, with vectors representing participants’ responses to each type of variable in the survey (controversial themes, cognitive traits, research topics, research methods, areas of psychology). For correlations including bibliometric models, see Extended Data Fig. 5.

**Extended Data Fig. 4:**
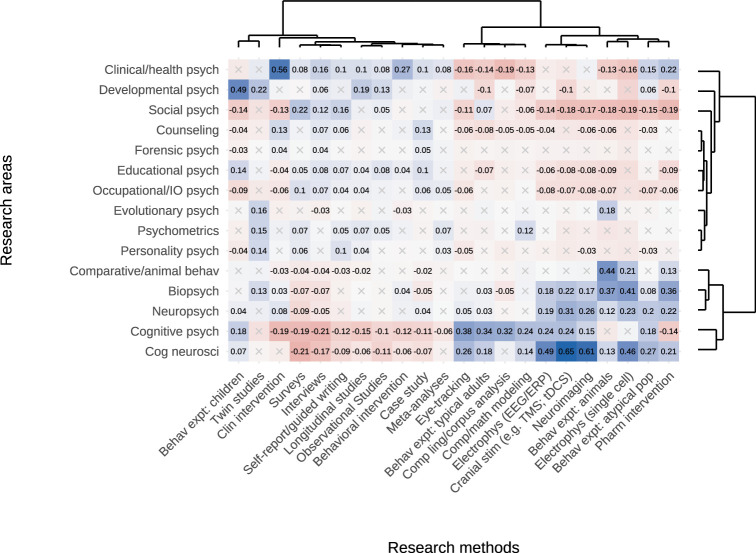
Regression coefficients for research areas as a function of research methods. Like Fig. 3, each cell here gives the regression coefficient for the simple model where individual research areas are regressed on individual research methods (though as the y-variable here is binary, unlike the controversial themes in Fig. 3, these are binomial regression coefficients expressed in logits). Cells marked with ‘x’ are non-significant (Bonferroni correction for multiple comparisons—the number of cells in the panel—yielding threshold *p* < .0001515). The margins of the plot show hierarchical clusters derived using Ward’s method. If the vectors of individual responses for research areas represent one high-dimensional space and the vectors of individual responses for research methods represent another high-dimensional space, then the correlation between all pairs of research-area vector cosine similarities and all pairs of research-methods cosine similarities is *ρ* = 0.22 (Figs. 5, 7), representing a conservative estimate for the overall association between these two types of response. For numeric results, see Appendix 2, Supplementary Table 6.

Figure 7 shows zero-order correlations between cosine similarities for the survey responses across spaces. Intuitively, we would expect that people in similar research areas would use similar methods and research similar topics (see Extended Data Fig. 4). Given these expectations, the observed correlations between research areas and methods (*ρ* = 0.22) and between research areas and topics (*ρ* = 0.13) provide a sense of scale—a benchmark for interpreting correlations between such abstract high-dimensional spaces. The association between controversial themes and cognitive traits (*ρ* = 0.1) was similar in magnitude to that between research areas and topics, and about half the size of the association between a respondent’s research area and the methods they reported using.

We used a series of regression analyses to examine whether cognitive traits were associated with responses to controversial themes, controlling for research areas, methods and topics (and report 99% CIs here). Regressing cosine similarities between controversial themes on the similarities for the other variables using multimember random effects^46^ (see Methods), we found that similarity in cognitive traits was a significant predictor of theme similarity even after controlling for other variables (*β* = 0.0218 [0.0213, 0.0223], *t* = 105.78). This was about half the effect size of the strongest predictor, research methods similarity (*β* = 0.0455 [0.0451, 0.0459], *t* = 291.83, Extended Data Fig. 6a) and similar in size to similarity of research areas (*β* = 0.0264 [0.0260, 0.0267], *t* = 191.61). Research topic similarity had a smaller effect (*β* = 0.0076 [0.0072, 0.0079], *t* = 58.06). In summary, researchers’ stances on the controversial themes were associated not only with what kind of research they do (methods, area and topics) but also with the cognitive traits that we assessed.

### Associations with research activity

Are researchers’ cognitive traits and stances on controversial themes associated with publishing different types of work? We investigated this by linking survey responses to respondents’ publications and bibliometric data. We measured research activities in three ways: (1) co-authorship (with whom did the respondents publish?), (2) content (a measure of semantic content of paper titles and abstracts) and (3) citations (what literature did the respondents’ papers build upon?).

To assess co-authorship similarity, we embedded psychology papers appearing in Web of Science (WoS) in a 128-dimensional ‘social space’ using their co-author information, thereby representing the article’s positions within the network of academic co-authors (see Methods). For the model of semantic content, we embedded the titles and abstracts of published papers into a 128-dimensional ‘semantic space’ using word embedding models^47^. We also embedded articles in a 128-dimensional ‘intellectual space’ as a function of their position within the set of cited previous research with network embedding models^48^.

**Extended Data Fig. 5:**
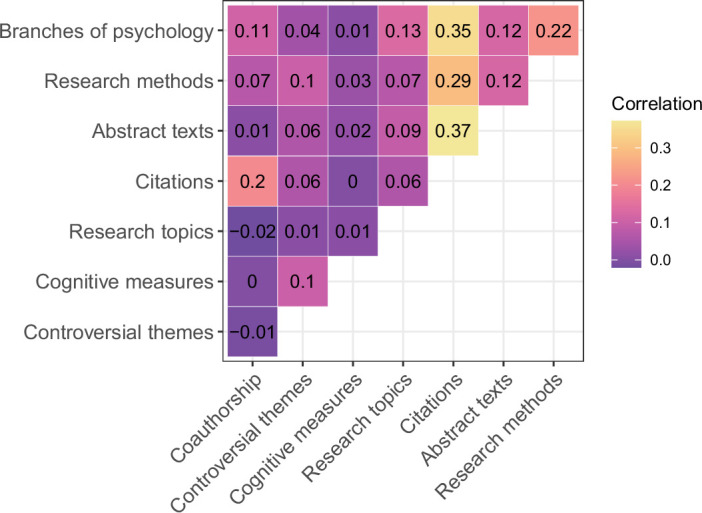
Cosine similarities for survey responses and bibliometric models across abstract spaces representing each class of variable. Correlations between cosine similarities across all pairs of participants, with vectors either consisting of participants’ responses to each type of question in the survey (controversial themes, cognitive traits, research topics, research methods, areas of psychology) or extracted from three models of publication space: word embedding model of texts of abstracts and titles from an author’s published articles; network models representing the works cited in each article (thus representing the knowledge base assumed in the article); and patterns of co-authorship across those articles.

Next, for those survey participants who gave consent to the anonymous linking of their survey answers with these bibliometric models, we obtained a vector representation of their position within each of the aforementioned bibliometric spaces. Thus, two people who co-author with a similar set of people will be represented by similar vectors in the co-author space; two people who cite similar literature will be represented by similar vectors in the citation space; two people whose abstracts and titles are similar will be represented by similar vectors in the semantic space (see Methods). Correlations between these publication models, as well as the survey data, are shown in Extended Data Fig. 5.

We first regressed co-authorship similarity on similarities in controversial themes, cognitive scales, research topics, research methods and areas of psychology (again with multimember random effects; as previously reporting 99% CIs). The strongest predictors of co-authorship similarity were research area similarity (*β* = 0.1224 [0.1217, 0.1230], *t* = 500.34), research methods similarity (*β* = 0.0986 [0.0979, 0.0993], *t* = 358.35) and research topic similarity (*β* = 0.0393 [0.0387, 0.0399], *t* = 170.2). This fits the intuition that scientists who do similar research tend to collaborate with a similar set of co-authors. Similarity in responses to controversial themes (*β* = 0.0146 [0.0136, 0.0155], *t* = 40.41) and cognitive trait similarity (*β* = 0.0061 [0.0052, 0.0070], *t* = 16.93) explained unique variance in co-authorship, albeit to a smaller degree.

**Extended Data Fig. 6:**
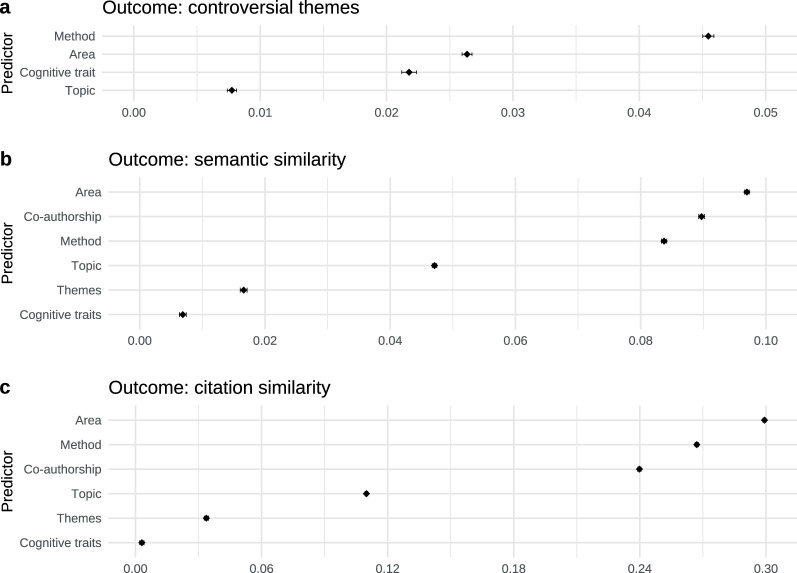
Regression coefficients predicting between-participant similarity in controversial themes and bibliometric models. Regression coefficients for each set of predictors on outcomes variables (**a**) controversial themes; (**b**) semantic model of abstracts and titles; (**c**) citation model. Points indicate standardized coefficients with error bars reflecting 99%CIs (though in some cases these are too small to be seen clearly, relative to the size of the point representing the coefficient). For similarities within the survey data (panel *a*), CIs are based on n=6932 participants, yielding 24,022,846 unordered pairwise comparisons. For similarities across survey and bibliometric data (panels *b*, *c*), CIs are based on n=4829 participants, yielding 11,657,206 unordered pairwise comparisons.

Next, we regressed semantic similarity (from participants’ published abstracts and titles) and citation similarity (reflecting the previous work that informs our participants’ published papers) on the same predictors, also including the aforementioned co-authorship similarity as a covariate (Extended Data Fig. 6b,c).

The strongest predictors of semantic similarity were research area (*β* = 0.0970 [0.0966, 0.0973], *t* = 725.54), co-authorship (*β* = 0.0897 [0.0893, 0.0901], *t* = 566.60), research methods (*β* = 0.0837 [0.0833, 0.0841], *t* = 559.78), research topics (*β* = 0.0476 [0.0473, 0.0479], *t* = 381.10), controversial themes (*β* = 0.0166 [0.0161, 0.0171], *t* = 85.18) and cognitive traits (*β* = 0.0069 [0.0064, 0.0074], *t* = 35.24).

Similarity in citations was associated with similarity in area of psychology (*β* = 0.2992 [0.2986, 0.2999], *t* = 1227.98), research methods (*β* = 0.2671 [0.2664, 0.2678], *t* = 979.13), co-authorship (*β* = 0.2398 [0.2390, 0.2405], *t* = 830.55), research topics (*β* = 0.1099 [0.1093, 0.1105], *t* = 482.08), controversial themes (*β* = 0.0337 [0.0328, 0.0347], *t* = 95.00) and cognitive traits (*β* = 0.0031 [0.0021, 0.0040], *t* = 8.60).

Similar to the results using co-authorship similarity, the works cited by an article and the semantic content of its abstract and title are most strongly associated with concrete features of the research ecology: the researchers’ broad area of psychology, their preferred research methods and their co-author network, with research topics having a more moderate association. Still, independent significant associations for researchers’ stances on controversial themes and their cognitive traits were detectable. These estimates are probably conservative, given that the vectors entering into the cosine similarities represent the full set of responses which, as Fig. 3 illustrates, includes individual associations with moderate effect sizes as well as associations that are weak or non-significant. Appendix 4 in Supplementary Information combines the above results using structural equation models.

## Discussion

Our findings provide evidence for the idea that science represents an ecology of diverse perspectives on contentious themes not straightforwardly resolved by the accumulation of more evidence.

Researchers often disagree. These disagreements can stem from differences in knowledge, training and expertise. But access to the same data and use of common methodology do not guarantee agreement. Instead, disciplines often fracture into schools of thought with diverging stances. We focused on divisions among researchers in psychological science, asking them about their stances on 16 controversial themes (Fig. 1). These included far-reaching issues such as the importance of the social environment on cognition, the connection between language and thought, and the utility of computational metaphors and mathematical models for human cognition.

Where researchers stood on these topics was correlated in both expected and unexpected ways with what the researchers study and how they study it. For example, use of neuroimaging was associated with having a lower belief in the importance of social environments. This suggests that it is not simply harder for those using neuroimaging techniques to study social groups—they are also less impressed by theories that view social context as foundational for understanding human cognition. More generally, the use of specific research methods comes bundled with foundational beliefs about what is important and what signifies plausibility and ultimately, truth itself. Differences in researchers’ stances on our controversial themes were also associated with individual differences in various cognitive traits ranging from ‘tolerance of ambiguity’, ‘creativity’ and ‘breadth of interest’ to the ‘need for cognition’, ‘visual imagery’ and ‘deliberation’.

Some epistemic commitments may reflect widely held stereotypes, such as women valuing social context more than men. Others such as the association between spatial imagery and math are large-scale replications of a pattern well known to education researchers (for review, see ref. ^45^). But similar to the bulk of an iceberg invisible beneath the water, most associations are much less obvious. These include the correlation of gender with realism, visual orientation with biological reductionism and many others (Fig. 3).

The results illustrate that incommensurability—difficulty of mapping between one scientific paradigm and another, whether due to differing theories, concepts, methods or measures^49^—is not purely an issue of abstract logic but is also a matter of individual cognition^50^. Divergence in stances on controversial themes and underlying cognitive traits do not simply mean that researchers solve problems in different ways. Rather, they reflect differences in how scientists approach, represent and reason about those problems. Moreover, our findings suggest that psychologists have different attitudes regarding what solutions are epistemically satisfying and worthy of contributing to the canon of psychological fact. These divisions run deep.

Insofar as many such differences in foundational beliefs are often hidden, one of the goals of this project was to make these divisions visible and available for public evaluation and critique. We do not suggest that certain cognitive traits are associated with worse science; rather that they may predict the approaches and answers that psychologists obtain in their research and thus might slow, side-track or indefinitely stall the resolution of truth claims within psychological science. In the worst-case scenario, cognitive differences could be exploited to prop up or even canonize an evidentially weak position that is intuitively attractive to researchers in positions of power.

An alternative cross-cutting division of cognitive labour, where cognitively diverse researchers study more topics with more approaches, might make for better psychological science^51,52^. Such cognitive diversity is particularly crucial at the stage of identifying questions and framing problems^53,54^. However, this would require that researchers who are not predisposed to frame and investigate psychological phenomena in a particular way would undertake what they might perceive as unsuitable or illogical research for the good of science. This poses the supreme challenge of allocating researchers and resources over questions and methods to resolve contentious issues.

### Limitations

#### Choice of field

This is a large-scale study of an entire field’s epistemic or theoretic commitments and their associations with cognitive traits. But a limitation is that we focused on only one field. We chose psychology, in part because it is (and aspires to be) pluralistic^24,55,56^. We do not think that the main claim here—that cognitive traits are associated with what kinds of ideas scientists find appealing, even holding their research areas and methods constant—is limited to psychology. We would expect to find similar patterns (although naturally involving different theoretic content) if we had chosen other social or behavioural sciences, and potentially even if we had chosen a harder science for this first look. Indeed, an assay of scientific status found psychology to be more similar to biology than sociology^57^, although Fig. 5a hints at some heterogeneity in this regard. However, it is important to demonstrate this empirically, and we look forward to future research that tests these ideas in other disciplines.

#### Effect sizes

Most effect sizes reported here are small in size. For instance, the correlations in Fig. 3c that passed the Bonferroni-corrected threshold for significance ranged from ∣*r*∣ = 0.04 to ∣*r*∣ = 0.18. Some potential worries regarding these effect sizes are (1) that they could be random noise, only rendered significant due to our large sample size (even after applying the Bonferroni corrections); and (2) if real, these effect sizes are too small to be consequential.

Regarding the first worry, we see our sample size (and the corresponding breadth of research areas, topics and methods illustrated in Extended Data Fig. 1) as a major strength of our approach—and crucial for investigating divisions of thought across a field as broad as psychology. We also address this concern in Supplementary Information, Appendix 5, assessing the robustness of results by repeatedly simulating a range of sample sizes from 300 to 3,000, rerunning benchmark analyses for each simulated sample size, illustrating how our reported associations would still be detectable at much smaller sample sizes.

More importantly, the consistency of the patterns in our data speaks against the idea that they are random noise. For instance, across panels of Fig. 3, the themes ‘social environment’, ‘context matters’ and ‘holistic view’ consistently cluster together, regardless of whether those clusters are defined according to associations with research methods, research topics, cognitive measures or gender. It is hard to see how such consistent patterns would emerge if each of those associations was just noise amplified by a large sample size.

Regarding the second worry, we think it is important to calibrate how large of an effect size would be plausible for this phenomenon. Previously reported^31^ correlations between personality traits and the sorts of theoretic commitments considered here ranged from *r* = 0.28 to *r* = 0.38. We consider this an upper estimate of plausibility, as that previous study examined fields from physics and mathematics to sociology and anthropology. Two average psychologists would likely be more similar in terms of theoretic commitments than an average physicist and an average sociologist, so effect sizes within a field should be smaller than the aforementioned effect sizes across such a broad range of fields.

Moreover, looking beyond academia for plausible effect sizes linking worldviews with attitudes to science, our recent estimate^58^ for the correlation between conservative political ideology and distrust in science in a large international sample is *r* = 0.23. If traits as easily observable as personality and political ideology have an effect on science beliefs just greater than *r* = 0.2, we would surely expect any effects of information processing dispositions to be harder to detect. After all, it is easier to judge whether one’s closest friends are extroverted or liberal than it is to judge whether they rely more on object versus spatial imagery.

Overall, if scientists’ thinking were strongly constrained by obvious traits, we all would surely have noticed this by now^54^, and it would offer a pessimistic view of science as being irredeemably under the thumb of human cognitive bias. If, instead, associations with cognitive traits are detectable (even in the conservative way we have attempted here, for instance by including all null effects from Fig. 3 in the vectors feeding into correlations in Fig. 7) as small-scale factors within the large-scale dynamics of publishing papers, then this would be more in keeping with the complex nature of science as an open-ended undertaking: one that is impacted by human cognition but not wholly subject to it.

It remains for future research to understand the practical importance of how small cognitive effects might play out during scientists’ development, training and career choices, social networks and complex research projects—topics at the intersection of psychology of science^20–22^ and science of science^59,60^.

#### Response rate and recruitment

A contribution of our study is that our findings are based on a large number of academics (7,973). However, this sample size nevertheless reflects a low response rate (3%) as the survey link was sent out to 278,692 email addresses comprising authors who had published in psychology journals as recorded in WoS. This includes people who may have retired, changed careers, as well as emails that are no longer active and filtered by their mail programs. A review of surveys with similar methods reveals comparable response rates: refs. ^61,62^ also used emails from WoS authors resulting in a 3% and 4% response rate, respectively, and ref. ^63^ obtained email addresses through web links on Twitter accounts, resulting in a 2% response rate.

**Extended Data Fig. 7:**
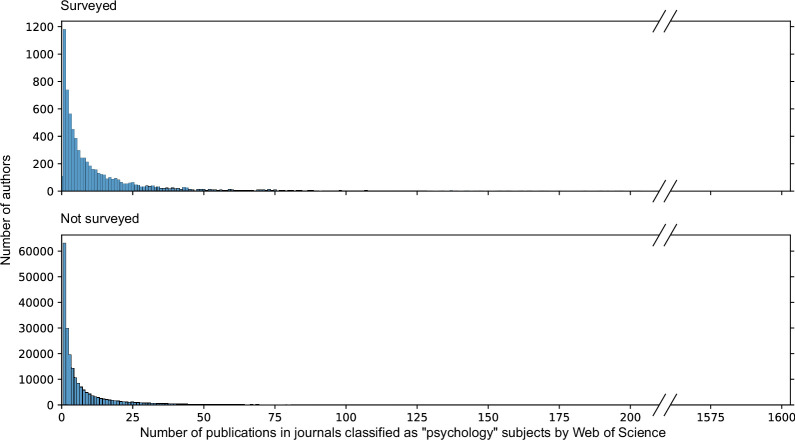
Distributions of publication counts, split between survey respondents and those who were invited but did not respond. Histograms showing the number of publications in journals classified as ‘psychology’ by WoS that we were able to index using the e-mail addresses to which invitations to participant were sent (using procedure identical to that described in Bibliometric models section of Materials). The top row shows the distribution of the survey respondents (median=6; mean=15.67). The bottom row shows the distribution of non-respondents, or e-mail addresses we emailed but did not get a survey response (median=3; mean=11.24). Note that the *x*-axis is broken after 210 for better visualization.

**Extended Data Fig. 8:**
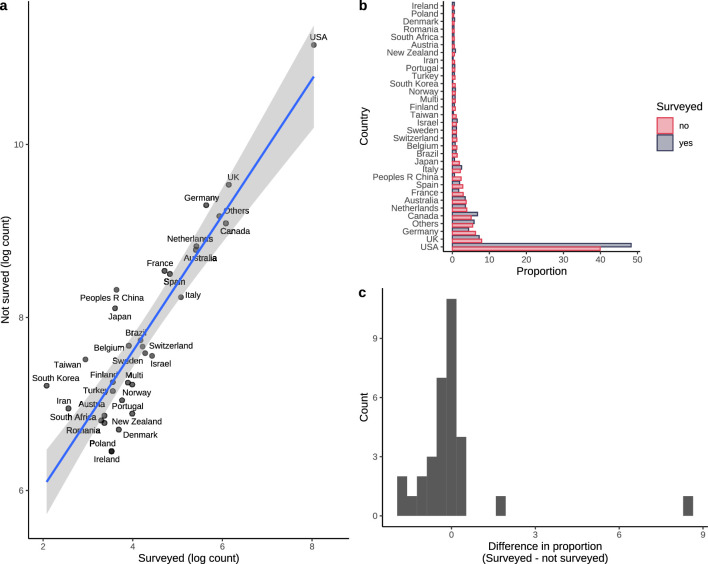
An overview of the inferred countries of email addresses to which we sent invitations, comparing those who responded with those who did not. Our survey did not ask about participant’s geographic locations, and generic email domains such as ‘gmail.com’ do not license inferences about location. However, for university (or other institutional) email domains we are generally able to infer what countries those institutions are based in. We were able to do so for 6421 respondents so, even if this is not full coverage, it represents the bulk of the survey data. (**a**) A comparison of the log counts of those who responded to the survey and those who did not, with a linear fit (predicted log mean, with 95% CIs); (**b**) A comparison of the proportion represented by each country (as a percentage), either as a proportion of our survey respondents (blue) or as a proportion of invitation emails that did not receive a response (red); (**c**) A histogram of the difference between each country’s proportions from panel **b**, subtracting the proportion who did not respond from the proportion that did. For example, the largest value in the histogram is a difference of 8.36% percentage points representing the USA because 48.28% of our survey respondents had email domains in the US whereas 39.92% of emails that were invited but did not respond had the same. Given these indications that there is a good match in respondent vs non-respondent countries apart from the USA, which is somewhat over-represented, we believe our sampling strategy reached a wide range of participants (even if it is not a representative sample of the global academic psychology community).

A related concern is whether our respondents are representative of academic psychologists. Extended Data Fig. 1 shows that our respondents represent a range of demographic characteristics, academic ranks, research topics and methods. Second, we compared respondents with non-respondents, using data available in WoS, which shows that the median first year of publication for respondents is 2009 versus 2008 for non-respondents, and the median most recent publication year is 2017 versus 2013, respectively. Extended Data Fig. 7 shows the distribution of the number of publications for respondents versus non-respondents, and Extended Data Fig. 8 shows the inferred countries they are from. Overall, these comparisons suggest that our survey respondents are largely similar to non-respondents, but they publish more (especially in psychology), have published more recently, and are slightly more likely to be located in the USA (the distribution of respondents over other countries is similar to the distribution among non-respondents, as Extended Data Fig. 8 illustrates). These differences should be taken into consideration when interpreting our results.

#### Selection and measurement of cognitive traits

To keep the survey to a manageable size, our assessment of cognitive traits/dispositions was necessarily brief and selective. The measures we used have high reliability (for example, the study validating tolerance of ambiguity^39^ reported a split-half reliability of 0.86 and a 6-month retest reliability of 0.63). However, high reliability in either sense can coexist with lifespan malleability and context dependence^38,64^. As with all retrospective question-based assessments, one must retain a degree of skepticism concerning people’s ability to accurately self-report their traits. That we find consistent and meaningful relationships despite these limitations of self-reported traits suggests the existence of an underlying measurable signal.

## Conclusions

Our findings support the idea that ‘science is a human enterprise, and understanding the development of scientific knowledge depends on an account of the thought processes of humans’^65^. Our research provides evidence for a pluralistic view of what constitutes an explanation and what makes explanations satisfying, persuasive and ultimately motivating for the community of scientists^66^. Variation in cognitive traits is associated with variation in perspectives on controversial topics and in turn, positions on controversial topics are associated with how likely it is for people to cite similar literatures and ultimately write similar articles. This also means that divisions in perspective run deep and pose a fundamental challenge for interdisciplinary research between communities with distinct epistemic commitments and composed of individuals with different cognitive profiles.

## Methods

### Participants

We extracted authors’ email addresses that appeared in articles published in 2,066 psychology journals in Web of Science (the full list of journals is provided in OSF at https://osf.io/zyec9/), yielding 278,692 email addresses. To these addresses, we emailed an invitation to participate, containing a link to the survey. Recipients were invited to forward the invitation to colleagues. In total, 7,973 people completed the survey, hosted on the Qualtrics platform. The final sample included 4,182 men, 3,683 women and 108 non-binary respondents. The modal decade of age was 30–39. Further participant information is provided in Extended Data Fig. 1.

Incomplete responses were not analysed. Participants who completed the survey were offered a chance to win gift vouchers in a lottery, or to choose a charity for us to donate to. The survey portion of the research project was judged exempt by the University of Chicago Social and Behavioral Sciences Institutional Review Board; the protocol for the additional stage of informed consent (for linking survey responses with models of bibliometric data, which appeared at the end of the survey) was approved by the same board (protocol number IRB14-1367-AM003).

### Materials

#### Themes

We generated 16 themes that we thought would provoke disagreement among psychologists. We piloted these on psychology graduate students mostly from the psychology department at UW-Madison to ensure that the descriptors were comprehensible and that both extremes were endorsed by at least some respondents. As this was the case, we considered these themes controversial. The themes and anchor labels are shown in Extended Data Table 1.

#### Cognitive traits

The full questionnaires for the scales described here are provided in OSF at https://osf.io/zyec9/. Phrases in single quotation marks below map onto labels in Fig. 3c.

Two scales probed differences in what are sometimes called ‘cognitive styles’. The Verbalizer-Visualizer Quotient (VVQ)^44^ comprised two subscales measuring people’s ‘verbal orientation’ (for example, ‘I prefer to read instructions about how to do something rather than have someone show me’) or ‘visual orientation’ (for example, ‘I find illustrations or diagrams help me when I’m reading’). Of note, the correlation between these two orientations is slightly positive (*r* = 0.11). We also included two subscales of the Object-Spatial Imagery and Verbal Questionnaire (OSIVQ)^29^ designed to probe vividnes of ‘object’ imagery (‘I can close my eyes and easily picture a scene that I have experienced’) and ‘spatial’ imagery (‘I can easily imagine and mentally rotate three-dimensional geometric figures’). The visual orientation from the VVQ is moderately correlated with both object imagery (*r* = 0.36) and spatial imagery (*r* = 0.40).

The next two scales focused on differences in preferred information processing. ‘Need for cognition’^67^ measures one’s disposition to engage in effortful cognition (‘I like to have the responsibility of handling a situation that requires a lot of thinking’). ‘Tolerance of ambiguity’^39^ taps into a person’s tendency to seek out and enjoy tasks that are poorly structured or lacking in clear cues (‘a problem has little attraction for me if I don’t think it has a solution [reverse scored]’).

We also took advantage of the finding that abbreviated scales can be used to simultaneously measure multiple constructs with sufficiently high construct validity^68^. ‘Cognitive structure’ and ‘deliberation’ reflect methodicalness (‘I plan my life logically’, ‘I like to act on a whim [reverse scored]’). ‘Aesthetics’ and ‘breadth of interest’ relate to intellectual curiosity (‘I love to hear about other countries and cultures’, ‘I read a large variety of books’). ‘Dominance’ is a facet of extroversion about leading or influencing others (‘I like being the authority who has everyone’s attention’, ‘I find it easy to manipulate others’).

Finally, we included three single-item scales probing three aspects of self-rated skills^69^: participants were asked to rate their ‘creative’, ‘analytic’ and ‘practical’ abilities from very weak to very strong.

### Procedure

Participants were first presented with an information screen explaining the general scope of the project, followed by a consent form. After providing informed consent to begin the study, participants indicated their stances on the 16 themes, presented in a random order. Participants responded using a slider, with 0% indicating complete agreement with the label presented on the left and 100% indicating complete agreement with the label on the right. The labels anchoring the left and right sides of the scale were randomized during presentation but were then realigned during analysis so that the 0% label always corresponds to the left label in Extended Data Table 1.

Participants then completed the cognitive trait surveys. These were grouped into blocks as reflected by the separate paragraphs above. The order of blocks was randomized. All responses were on 5-point Likert scales (from ‘strongly disagree’ to ‘strongly agree’) except for the three self-reported ability scales which were given as percentages from low to high ability.

Participants then provided biographic information (age, gender, rank) and free-text responses indicating up to 5 topics that they researched (for example, episodic memory, categorization, language, sleep).

They were then given a list of broad areas of psychology (for example, social psychology, cognitive psychology), and clicked on check boxes to select whichever they worked in. An ‘other’ check box allowed further open-text responses if their research areas were not given. Similarly, they clicked on check boxes to select whichever methods they used in research (as opposed to practice). Again, an ‘other’ check box allowed for further free-text responses if their preferred methods were not given.

In the final part of the survey, which required an extra stage of consent, we explained that we wanted to understand how responses to the survey mapped onto people’s published work. If participants consented, we would use their provided email addresses to create a link between their survey responses and the bibliometric models. We explained that once this link was made, the email addresses would be discarded, so any mapping between survey responses and vectors in the bibliometric models would be anonymous.

### Bibliometric models

To assess the distance of authors in terms of their linguistic patterns (that is, what words they use; the semantic model), reference patterns (that is, what articles they cite in their articles; the citation model) and co-authorship patterns (that is, what group of people they write their articles with; the co-authorship model), we embedded individual authors into three geometric vector spaces with 128 dimensions and calculated their distance in those spaces. To do this, we first collected the bibliographic data from the WoS database. We only used articles classified as being written in English and published in journals that were classified as psychology journals in the WoS database (that is, the journal’s subject had the word ‘psychology’ in the WoS database). For the semantic model, we also linked the articles in the WoS database to the Microsoft Academic Graph (MAG) database via digital object identifier to retrieve additional titles and abstracts missing from the WoS database (note that the MAG database was not used for the other two bibliometric models).

Using the bibliographic data, we embedded the articles into three different vector spaces. For the semantic model, we embedded 733,133 articles that had valid titles and abstracts in the database by running the Doc2vec^47^ algorithm implemented in the Python library Gensim^70^ on the found titles and abstracts. Doc2vec and related unsupervised machine learning models have transformed modern natural language processing. These models ‘discover’ semantics from linguistic context and validate the distribution hypothesis that words occurring in the same contexts tend to have similar meanings by performing at human level on analogy tests^47,71^, question answering^72,73^ and a wide range of language understanding tasks. It has also been demonstrated that embedding texts produced by persons in given times and places can replicate surveyed associations among people from those same times and places^74–77^. Here we operationalized the ‘intellectual’ distance between papers as the cosine similarity between paper titles+abstracts. This approach produces estimates of greater semantic similarity than bibliometric approaches for assessing the co-citation of articles or journals^78^ while not assuming that the compared works frame themselves with respect to the same previous work.

For the citation model and the co-authorship models, we first constructed a citation network and a co-authorship network using the data in the WoS database, respectively. For the citation network, the citation between the articles was treated as directed unweighted edges (total number of citations: 16,495,908), and the articles were treated as nodes (total number of articles: 1,190,495). Only articles that had valid citation data in the database were used to construct the citation network. For the co-authorship network, we constructed a network where the articles were treated as nodes (total number of articles: 511,508), and the nodes were connected with an undirected edge if they shared at least one author (total number of co-author connections: 9,228,646). The edges were weighted by the number of authors the articles shared. In addition, to account for the possibility that first or last authors contribute more towards a paper’s framing, we upweighted edges connected by first or last authors by a factor of 4. The articles were included in the network if the first name, the last name and the organization of the author(s) were in the database. The articles were considered to be written by the same author if and only if the first name, the last name and the organization of the authors were matched between the articles. After building the networks, we ran the Node2vec algorithm^48^ (algorithm code available in GitHub at https://github.com/eliorc/node2vec) on the network to embed the articles into each vector space. Network embedding models have revolutionized network prediction and description, just as text embedding models have transformed natural language processing^79^. Additional details on the bibliometric models are provided in Supplementary Information, Appendix 3.

After embedding the articles into the vector spaces, we calculated the average of vectors corresponding to the articles that each survey participant published, yielding vectors for each author. To acquire the articles that each survey participant wrote, we first queried the email address to the WoS database (note that this step was done in the entire WoS database we had access to, not just the psychology journals) and collected all first name, last name and organization tuples (for example, Jane, Doe, XXX University) that matched the email address. Then, we collected all articles that matched the set in the WoS database and considered these articles to be authored by the survey participant. This process was only done for participants who agreed to have their data cross-referenced to the bibliographic database and provided their email addresses. In the semantic model, we were able to link 6,637 survey participants to at least one valid article (mean = 11.91 articles per participant, s.d. = 18.42). In the citation model and co-authorship model, we found 6,779 participants (mean = 13.67 articles per participant, s.d. = 22.60) and 5,708 participants (mean = 11.42 articles per participant, s.d. = 15.61) that had at least one article represented in the model, respectively. Finally, we used the cosine similarity between the author vector pairs to calculate the similarity between the authors in each model, which was used in the statistical analysis (see below).

### Regression with multimember random effects

As authors appeared multiple times in the analysis of similarities across high-dimensional spaces, we required a random effect for each author in the relevant regression models. Each row in the data represents the similarity between a pair of authors (author A and author B), but as their ordering does not matter, it would be inappropriate to include one random intercept for author A and another for author B (a given participant could appear in the author A column on one occasion and in the author B column on another occasion). Multimembership random effects (with R package lmerMultiMember^46^) allow us to specify a random effect, with pairs of authors as unordered levels.

### Reporting summary

Further information on research design is available in the Nature Portfolio Reporting Summary linked to this article.

## Supplementary information

Supplementary Information Appendices 1–5 containing Supplementary Tables 1–9 and Figs. 1–3.

Reporting Summary

## Figures and Tables

**Extended Data Table 1:**
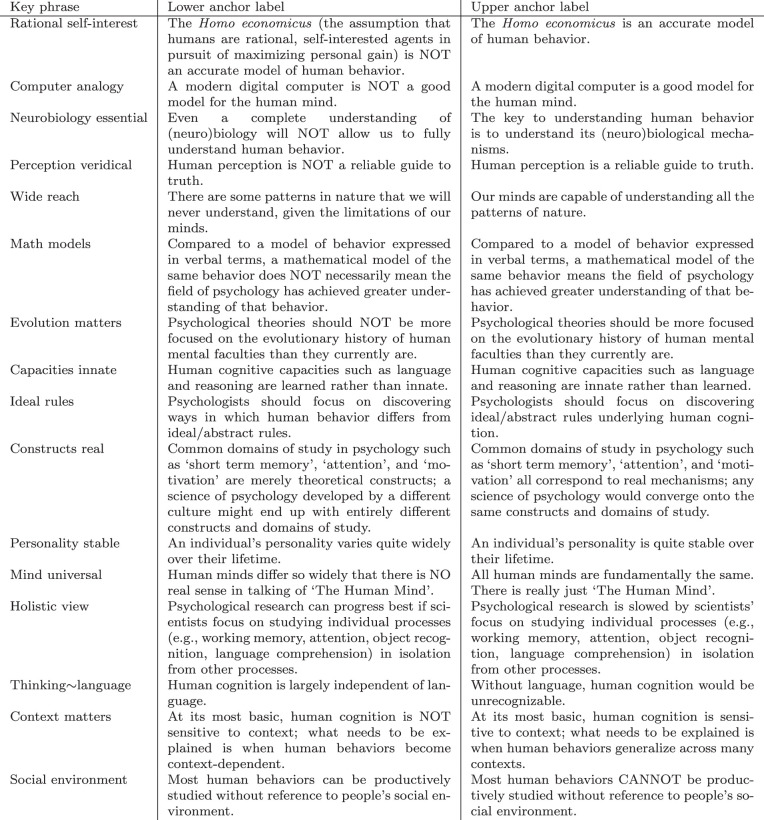
Key-word identifiers for the 16 controversial themes, with the descriptive labels for both ends of the response scales displayed to respondents

Left-right orientation of these was randomized per participant per scale, so ‘lower’ and ‘upper’ here refer to the positive/negative orientation of the axes in analyses. Responses range from 0% (completely agree with lower anchor label) to 100% (completely agree with upper anchor label).

## Data Availability

Anonymous survey data are available in OSF at https://osf.io/zyec9/ (ref. ^80^). The wiki of this repository describes cases where we either censored or grouped data to hide rare responses before making data open. Microsoft Academic Graph (MAG) data can be obtained freely for download at https://www.microsoft.com/en-us/research/project/microsoft-academic-graph/. Web of Science (WoS) data can be obtained for a licensing fee from Clarivate https://clarivate.com/. Our open data include anonymous codes that link anonymous survey responses with the anonymous, aggregated bibliometric model (specifically, aggregated cosine similarity metrics). We cannot provide non-aggregated versions of these linked data, including links to MAG/WoS data, as that would involve breaching the terms of informed consent (as specified by our IRB) by making it possible to infer participant identities.

## Code availability

Analysis scripts (in R) are available in OSF at https://osf.io/zyec9/ (ref. ^80^).
