# Supplementary material for: Differences in psychologists’ cognitive traits are associated with scientific divides
Source: Nat Hum Behav. 2025 Apr 17;9(6):1147–61. doi: 10.1038/s41562-025-02153-1 (PMC12185325; doi:10.1038/s41562-025-02153-1)
Supplement: Supplementary file 1 — Appendices 1–5 containing Supplementary Tables 1–9 and Figs. 1–3. [file 41562_2025_2153_MOESM1_ESM.pdf]

nature human behaviour

Supplementary information https://doi.org/10.1038/s41562-025-02153-1

Differences in psychologists' cognitive traits are associated with scientific divides

In the format provided by the authors and unedited

Supplementary Materials: Contents

Appendix 1 Bayesian models of responses to controversial themes 2

Appendix 2 Extended regression outputs 6

Appendix 3 Additional details on the bibliometric models 37

Appendix 4 Structural equation models 38

Appendix 5 Robustness checks 43

References 45

Appendix 1 Bayesian models of responses to controversial themes

To model the distributions of responses shown in Figure1 of the main text, we developed custom Bayesian models to account for various idiosyncratic features of the data:

- The response format was a percentage scale, so responses map onto the range [0, 1]. Beta regressions are apt for modeling data in (0, 1) so they are an obvious choice here, but as they cannot model 0s or 1s, this necessitates additional model components ('inflation' components) for responses at either extreme end of the scale.
- Additionally, a number of themes showed a spike at exactly 50%, which represents a distinctive response strategy: Participants had to move the slider to progress to the next item in the survey, so if they wanted to answer exactly 50% rather than some number near 50% (perhaps to avoid making a choice, no matter how trivial, for either left- or right-wards movement), this necessitated moving the slider off the mid-point where it was initially, then moving it back to 50%. We thus included an additional inflation component for responses exactly at the mid-point.
- Many of the themes showed a bimodal response pattern, so all responses that were not one of the unique strategies above (i.e., everything not at 0, 0.5 or 1) were handled with a mixture model. This estimated parameters for two Beta distributions, each representing one of two latent groups in the data.

Thus, each theme is modeled as a combination of inflation components (0, 1 or 0.5, highlighted with lighter blue in Figure 1 and mixture components (everything else, shown in darker blue in Figure 1). The Stan code for these custom models is available at `https://osf.io/zyec9/`.

Supplementary Table 1 shows the model estimates. Below we show how the table contents correspond to the above points and illustrate how to interpret the estimates using the first theme below ('Rational self-interest') as an example.

For each theme, the first inflation component is a logistic model of how many participants responded at *either* end of the scale. As parameter 'Zero or one' is a proportion, the value 0.144 implies that 14.4% of participants moved the slider all the way to the right *or* to the left of the scale for theme 'Rational self-interest'.

Conditional on having moved the slider to either extreme, the next component is a logistic model how many extreme responses that were at the upper rather than the lower extreme. As parameter 'Conditional one' is a proportion, the estimate 0.028 means that 2.8% of extreme responses were 1s, representing full agreement with the upper anchor label for 'Rational self-interest' in Table Supplementary Table 1. In turn, this implies that 97.2% (1-0.028=0.972) of extreme responses were 0s, representing full agreement with the lower anchor label in Supplementary Table 1.

Conditional on *not* having moved the slider to either extreme, the next component is a logistic model of how many responses were at 50%. Again, as parameter 'Mid' is a

proportion, the estimate 0.027 means that 2.7% of non-extreme participants purposefully chose the exact mid-point for 'Rational self-interest'.

The remaining components are mixture components, modeling all non-inflation responses (everything other than 0, 0.5 or 1) as a mixture of two latent groups. Column 'Proportion' shows the estimated proportion of responses falling into each group (model θ). Thus, 74.8% of non-inflation responses were in Group 1 and 25.2% were in Group 2.

Each latent group is modeled as a Beta distribution with a mean (a percentage-response transformation of the model's logistic μ parameter) and a precision parameter (model ϕ). Thus, the mean response for the first latent group was 20.7% on the response scale and for the second latent group it was 63.5%.

Supplementary Table 1: Estimated parameters (with 95% Credibility Intervals) from Bayesian models of responses to each controversial theme.

Theme	Component	Group	Proportion	Parameter	Estimate (95%CIs)
Rational self-interest	Inflation			Zero or one	0.144 [0.138, 0.15]
				Conditional one	0.028 [0.02, 0.036]
				Mid	0.027 [0.024, 0.031]
	Mixture	1	0.748 [0.72, 0.772]	Mean	0.207 [0.2, 0.214]
				Precision	9.477 [8.899, 10.094]
		2	0.252 [0.228, 0.28]	Mean	0.635 [0.607, 0.658]
				Precision	7.325 [6.168, 8.484]
Computer analogy	Inflation			Zero or one	0.122 [0.116, 0.128]
				Conditional one	0.04 [0.03, 0.05]
				Mid	0.047 [0.043, 0.051]
	Mixture	1	0.58 [0.473, 0.678]	Mean	0.206 [0.196, 0.218]
				Precision	9.748 [8.913, 10.686]
		2	0.252 [0.228, 0.28]	Mean	0.635 [0.607, 0.658]
				Precision	7.325 [6.168, 8.484]
Neurobiology essential	Inflation			Zero or one	0.155 [0.149, 0.162]
				Conditional one	0.82 [0.069, 0.095]
				Mid	0.037 [0.034, 0.041]
	Mixture	1	0.701 [0.676, 0.723]	Mean	0.222 [0.215, 0.229]
				Precision	8.757 [8.222, 9.333]
		2	0.299 [0.277, 0.324]	Mean	0.681 [0.658, 0.7]
				Precision	6.573 [5.667, 7.479]
Perception veridical	Inflation			Zero or one	0.082 [0.077, 0.087]
				Conditional one	0.081 [0.065, 0.099]
				Mid	0.064 [0.059, 0.069]
	Mixture	1	0.608 [0.537, 0.667]	Mean	0.24 [0.227, 0.253]
				Precision	9.388 [8.581, 10.227]
		2	0.392 [0.333, 0.463]	Mean	0.593 [0.55, 0.629]
				Precision	5.84 [4.813, 6.946]
Wide reach	Inflation			Zero or one	0.11 [0.104, 0.116]
				Conditional one	0.153 [0.133, 0.173]
				Mid	0.044 [0.04, 0.048]
	Mixture	1	0.775 [0.755, 0.793]	Mean	0.279 [0.271, 0.287]
				Precision	6.663 [6.273, 7.081]
		2	0.225 [0.228, 0.28]	Mean	0.635 [0.607, 0.658]
				Precision	7.325 [6.168, 8.484]

Math models	Inflation			Zero or one	0.092 [0.087, 0.98]
				Conditional one	0.248 [0.222, 0.275]
				Mid	0.058 [0.053, 0.062]
	Mixture	1	0.505 [0.469, 0.539]	Mean	0.243 [0.231, 0.255]
				Precision	9.01 [8.217, 9.81]
		2	0.495 [0.461, 0.531]	Mean	0.652 [0.633, 0.669]
				Precision	6.509 [5.808, 7.234]
Evolution matters	Inflation			Zero or one	0.066 [0.061, 0.07]
				Conditional one	0.454 [0.418, 0.49]
				Mid	0.102 [0.097, 0.108]
	Mixture	1	0.509 [0.026, 0.991]	Mean	0.464 [0.421, 0.489]
				Precision	3.282 [2.364, 3.685]
		2	0.491 [0.009, 0.974]	Mean	0.52 [0.487, 0.604]
				Precision	3.479 [1.912, 4.406]
Capacities innate	Inflation			Zero or one	0.042 [0.038, 0.046]
				Conditional one	0.54 [0.495, 0.584]
				Mid	0.106 [0.101, 0.112]
	Mixture	1	0.548 [0.096, 0.995]	Mean	0.44 [0.288, 0.502]
				Precision	4.414 [3.171, 7.906]
		2	0.452 [0.005, 0.904]	Mean	0.557 [0.501, 0.663]
				Precision	3.757 [1.642, 5.963]
Ideal rules	Inflation			Zero or one	0.028 [0.025, 0.31]
				Conditional one	0.504 [0.449, 0.558]
				Mid	0.188 [0.181, 0.196]
	Mixture	1	0.311 [0.005, 0.994]	Mean	0.465 [0.396, 0.507]
				Precision	3.18 [1.358, 4.346]
		2	0.689 [0.006, 0.995]	Mean	0.524 [0.505, 0.573]
				Precision	4.029 [1.515, 4.839]
Constructs real	Inflation			Zero or one	0.058 [0.053, 0.062]
				Conditional one	0.446 [0.408, 0.484]
				Mid	0.031 [0.028, 0.033]
	Mixture	1	0.481 [0.454, 0.508]	Mean	0.312 [0.299, 0.326]
				Precision	7.794 [6.887, 8.328]
		2	0.519 [0.492, 0.546]	Mean	0.721 [0.711, 0.731]
				Precision	9.237 [8.526, 9.964]
Personality stable	Inflation			Zero or one	0.024 [0.021, 0.027]
				Conditional one	0.353 [0.298, 0.41]
				Mid	0.043 [0.04, 0.047]
	Mixture	1	0.432 [0.408, 0.458]	Mean	0.336 [0.324, 0.35]
				Precision	9.301 [8.349, 10.243]
		2	0.568 [0.542, 0.592]	Mean	0.699 [0.692, 0.706]
				Precision	12.397 [11.585, 13.212]
Mind universal	Inflation			Zero or one	0.032 [0.029, 0.036]
				Conditional one	0.625 [0.577, 0.672]
				Mid	0.067 [0.062, 0.072]
	Mixture	1	0.664 [0.573, 0.737]	Mean	0.511 [0.491, 0.527]
				Precision	3.745 [3.586, 3.992]
		2	0.336 [0.263, 0.427]	Mean	0.675 [0.662, 0.687]
				Precision	11.803 [8.844, 15.933]
Holistic view	Inflation			Zero or one	0.042 [0.038, 0.046]
				Conditional one	0.821 [0.786, 0.853]

				Mid	0.117 [0.111, 0.123]
	Mixture	1	0.536 [0.355, 0.691]	Mean	0.498 [0.434, 0.537]
				Precision	3.933 [3.53, 4.622]
		2	0.464 [0.309, 0.645]	Mean	0.671 [0.647, 0.695]
				Precision	6.758 [5.766, 8.186]
Thinking∼language	Inflation			Zero or one	0.055 [0.051, 0.059]
				Conditional one	0.841 [0.812, 0.869]
				Mid	0.052 [0.048, 0.056]
	Mixture	1	0.302 [0.25, 0.375]	Mean	0.364 [0.327, 0.414]
				Precision	7.13 [5.566, 8.709]
		2	0.698 [0.625, 0.75]	Mean	0.724 [0.711, 0.739]
				Precision	8.427 [7.735, 9.178]
Context matters	Inflation			Zero or one	0.058 [0.054, 0.063]
				Conditional one	0.929 [0.908, 0.947]
				Mid	0.076 [0.071, 0.081]
	Mixture	1	0.236 [0.153, 0.414]	Mean	0.434 [0.352, 0.576]
				Precision	6.126 [3.545, 8.616]
		2	0.764 [0.586, 0.847]	Mean	0.754 [0.745, 0.765]
				Precision	10.375 [9.423, 12.188]
Social environment	Inflation			Zero or one	0.138 [0.132, 0.145]
				Conditional one	0.958 [0.948, 0.967]
				Mid	0.041 [0.038, 0.046]
	Mixture	1	0.288 [0.219, 0.356]	Mean	0.5 [0.435, 0.554]
				Precision	3.977 [3.339, 4.925]
		2	0.712 [0.644, 0.781]	Mean	0.798 [0.79, 0.805]
				Precision	9.432 [8.806, 10.076]

Appendix 2 Extended regression outputs

The first four tables in this section present the model outputs for controversial themes regressed on research areas (Supplementary Table 2), research methods (Supplementary Table 3), cognitive traits (Supplementary Table 4) and gender (Supplementary Table 5). These are summarized in Fig. 3 of the main text, with a subset of the cognitive trait results (for tolerance of ambiguity) also appearing in Fig. 2. Unlike in the previous Appendix, 'CIs' here represent frequentist confidence intervals. Next is a similar table for research areas regressed on research methods, corresponding to Extended Data Figure 4.

Following that is a table of regression coefficients for latent theme factor scores as a function of cognitive traits (Supplementary Table 7), which are summarized in Fig. 6b of the main text.

Supplementary Table 2: Regression coefficients for controversial themes as a function of research area (each a separate regression model). Results correspond to Fig. 3a in the main text. Regression coefficients (b) represent logits, with the outcome (theme) standardized. P-values in gray reflect cases that failed to meet the Bonferroni-adjusted significance threshold discussed in the main text ($p < .000208$).

Theme	Research area	Coefficient (b)	CIs	t	p
Constructs real	Biopsych	0.172	[0.095, 0.249]	4.398	<2.08e-04
Thinking language	Biopsych	-0.184	[-0.261, -0.107]	-4.707	<2.08e-04
Math models	Biopsych	0.197	[0.12, 0.273]	5.031	<2.08e-04
Wide reach	Biopsych	0.069	[-0.008, 0.146]	1.763	0.078
Rational self-interest	Biopsych	0.051	[-0.026, 0.128]	1.298	0.194
Neurobiology essential	Biopsych	0.712	[0.637, 0.787]	18.570	<2.08e-04
Holistic view	Biopsych	-0.059	[-0.136, 0.017]	-1.514	0.130
Personality stable	Biopsych	0.122	[0.045, 0.199]	3.119	0.002
Context matters	Biopsych	-0.067	[-0.143, 0.01]	-1.704	0.088
Capacities innate	Biopsych	0.099	[0.023, 0.176]	2.540	0.011
Ideal rules	Biopsych	0.050	[-0.027, 0.127]	1.276	0.202
Evolution matters	Biopsych	0.297	[0.22, 0.373]	7.612	<2.08e-04
Computer analogy	Biopsych	-0.133	[-0.21, -0.056]	-3.402	0.001
Mind universal	Biopsych	0.107	[0.03, 0.184]	2.737	0.006
Social environment	Biopsych	-0.246	[-0.323, -0.17]	-6.303	<2.08e-04
Perception veridical	Biopsych	-0.095	[-0.172, -0.018]	-2.422	0.015
Constructs real	Clinical/health psych	-0.085	[-0.134, -0.035]	-3.351	0.001
Thinking language	Clinical/health psych	0.063	[0.014, 0.113]	2.504	0.012
Math models	Clinical/health psych	-0.103	[-0.153, -0.054]	-4.103	<2.08e-04
Wide reach	Clinical/health psych	-0.129	[-0.179, -0.08]	-5.127	<2.08e-04
Rational self-interest	Clinical/health psych	0.133	[0.084, 0.182]	5.280	<2.08e-04
Neurobiology essential	Clinical/health psych	-0.092	[-0.141, -0.042]	-3.634	<0.001
Holistic view	Clinical/health psych	0.149	[0.1, 0.199]	5.933	<2.08e-04
Personality stable	Clinical/health psych	0.083	[0.033, 0.132]	3.281	0.001
Context matters	Clinical/health psych	-0.024	[-0.074, 0.025]	-0.964	0.335
Capacities innate	Clinical/health psych	0.029	[-0.02, 0.079]	1.165	0.244

Supplementary Table 2: Regression coefficients, research areas *(continued)*

Theme	Research area	Coefficient (b)	CIs	t	p
Ideal rules	Clinical/health psych	-0.137	[-0.187, -0.088]	-5.441	<2.08e-04
Evolution matters	Clinical/health psych	-0.004	[-0.054, 0.045]	-0.173	0.863
Computer analogy	Clinical/health psych	0.036	[-0.013, 0.086]	1.445	0.149
Mind universal	Clinical/health psych	-0.158	[-0.207, -0.108]	-6.256	<2.08e-04
Social environment	Clinical/health psych	0.254	[0.205, 0.303]	10.131	<2.08e-04
Perception veridical	Clinical/health psych	-0.136	[-0.186, -0.087]	-5.405	<2.08e-04
Constructs real	Cog neurosci	0.114	[0.055, 0.172]	3.821	<2.08e-04
Thinking language	Cog neurosci	-0.164	[-0.223, -0.106]	-5.521	<2.08e-04
Math models	Cog neurosci	0.386	[0.328, 0.444]	13.088	<2.08e-04
Wide reach	Cog neurosci	0.064	[0.005, 0.122]	2.145	0.032
Rational self-interest	Cog neurosci	-0.058	[-0.116, 0.001]	-1.943	0.052
Neurobiology essential	Cog neurosci	0.753	[0.697, 0.809]	26.333	<2.08e-04
Holistic view	Cog neurosci	-0.206	[-0.265, -0.148]	-6.945	<2.08e-04
Personality stable	Cog neurosci	0.032	[-0.027, 0.09]	1.065	0.287
Context matters	Cog neurosci	-0.116	[-0.175, -0.058]	-3.906	<2.08e-04
Capacities innate	Cog neurosci	0.041	[-0.017, 0.1]	1.380	0.168
Ideal rules	Cog neurosci	0.204	[0.146, 0.262]	6.866	<2.08e-04
Evolution matters	Cog neurosci	0.185	[0.127, 0.243]	6.227	<2.08e-04
Computer analogy	Cog neurosci	-0.039	[-0.097, 0.02]	-1.294	0.196
Mind universal	Cog neurosci	0.217	[0.159, 0.275]	7.302	<2.08e-04
Social environment	Cog neurosci	-0.593	[-0.65, -0.536]	-20.420	<2.08e-04
Perception veridical	Cog neurosci	-0.024	[-0.083, 0.034]	-0.817	0.414
Constructs real	Cognitive psych	0.177	[0.129, 0.226]	7.201	<2.08e-04
Thinking language	Cognitive psych	-0.078	[-0.127, -0.03]	-3.165	0.002
Math models	Cognitive psych	0.327	[0.279, 0.375]	13.367	<2.08e-04
Wide reach	Cognitive psych	0.076	[0.027, 0.124]	3.073	0.002
Rational self-interest	Cognitive psych	-0.093	[-0.142, -0.045]	-3.775	<2.08e-04
Neurobiology essential	Cognitive psych	0.141	[0.093, 0.189]	5.720	<2.08e-04
Holistic view	Cognitive psych	-0.282	[-0.33, -0.234]	-11.491	<2.08e-04
Personality stable	Cognitive psych	-0.029	[-0.078, 0.019]	-1.178	0.239
Context matters	Cognitive psych	-0.083	[-0.131, -0.034]	-3.348	0.001
Capacities innate	Cognitive psych	0.056	[0.008, 0.105]	2.280	0.023
Ideal rules	Cognitive psych	0.236	[0.188, 0.284]	9.606	<2.08e-04
Evolution matters	Cognitive psych	0.063	[0.015, 0.112]	2.556	0.011
Computer analogy	Cognitive psych	0.119	[0.071, 0.168]	4.837	<2.08e-04
Mind universal	Cognitive psych	0.327	[0.279, 0.375]	13.372	<2.08e-04
Social environment	Cognitive psych	-0.547	[-0.594, -0.5]	-22.844	<2.08e-04
Perception veridical	Cognitive psych	0.092	[0.043, 0.14]	3.708	<0.001
Constructs real	Comparative/animal behav	0.032	[-0.086, 0.15]	0.533	0.594
Thinking language	Comparative/animal behav	-0.399	[-0.517, -0.281]	-6.644	<2.08e-04
Math models	Comparative/animal behav	0.109	[-0.009, 0.227]	1.815	0.070
Wide reach	Comparative/animal behav	0.164	[0.046, 0.282]	2.732	0.006
Rational self-interest	Comparative/animal behav	-0.025	[-0.143, 0.094]	-0.407	0.684
Neurobiology essential	Comparative/animal behav	0.446	[0.328, 0.564]	7.431	<2.08e-04

Supplementary Table 2: Regression coefficients, research areas *(continued)*

Theme	Research area	Coefficient (b)	CIs	t	p
Holistic view	Comparative/animal behav	-0.044	[-0.162, 0.074]	-0.728	0.467
Personality stable	Comparative/animal behav	-0.018	[-0.136, 0.1]	-0.301	0.763
Context matters	Comparative/animal behav	-0.062	[-0.18, 0.056]	-1.028	0.304
Capacities innate	Comparative/animal behav	0.108	[-0.01, 0.226]	1.789	0.074
Ideal rules	Comparative/animal behav	0.137	[0.019, 0.255]	2.280	0.023
Evolution matters	Comparative/animal behav	0.778	[0.661, 0.895]	13.058	<2.08e-04
Computer analogy	Comparative/animal behav	-0.143	[-0.261, -0.025]	-2.383	0.017
Mind universal	Comparative/animal behav	0.166	[0.048, 0.284]	2.762	0.006
Social environment	Comparative/animal behav	-0.293	[-0.411, -0.175]	-4.877	<2.08e-04
Perception veridical	Comparative/animal behav	-0.150	[-0.268, -0.032]	-2.490	0.013
Constructs real	Counseling	-0.285	[-0.372, -0.197]	-6.367	<2.08e-04
Thinking language	Counseling	0.066	[-0.022, 0.154]	1.479	0.139
Math models	Counseling	-0.308	[-0.396, -0.221]	-6.895	<2.08e-04
Wide reach	Counseling	-0.142	[-0.23, -0.054]	-3.171	0.002
Rational self-interest	Counseling	0.140	[0.052, 0.228]	3.129	0.002
Neurobiology essential	Counseling	-0.213	[-0.301, -0.125]	-4.757	<2.08e-04
Holistic view	Counseling	0.105	[0.017, 0.193]	2.348	0.019
Personality stable	Counseling	-0.150	[-0.237, -0.062]	-3.340	0.001
Context matters	Counseling	0.103	[0.015, 0.191]	2.300	0.021
Capacities innate	Counseling	-0.083	[-0.171, 0.004]	-1.861	0.063
Ideal rules	Counseling	-0.227	[-0.315, -0.139]	-5.069	<2.08e-04
Evolution matters	Counseling	0.031	[-0.057, 0.119]	0.687	0.492
Computer analogy	Counseling	-0.064	[-0.152, 0.024]	-1.424	0.154
Mind universal	Counseling	-0.280	[-0.368, -0.192]	-6.263	<2.08e-04
Social environment	Counseling	0.350	[0.262, 0.437]	7.831	<2.08e-04
Perception veridical	Counseling	-0.016	[-0.104, 0.072]	-0.352	0.725
Constructs real	Developmental psych	-0.065	[-0.119, -0.011]	-2.369	0.018
Thinking language	Developmental psych	0.141	[0.087, 0.195]	5.123	<2.08e-04
Math models	Developmental psych	-0.100	[-0.154, -0.046]	-3.634	<0.001
Wide reach	Developmental psych	0.002	[-0.052, 0.056]	0.071	0.944
Rational self-interest	Developmental psych	0.033	[-0.021, 0.087]	1.214	0.225
Neurobiology essential	Developmental psych	-0.072	[-0.126, -0.019]	-2.637	0.008
Holistic view	Developmental psych	0.200	[0.147, 0.254]	7.313	<2.08e-04
Personality stable	Developmental psych	0.015	[-0.039, 0.069]	0.533	0.594
Context matters	Developmental psych	0.089	[0.036, 0.143]	3.255	0.001
Capacities innate	Developmental psych	-0.133	[-0.187, -0.079]	-4.851	<2.08e-04
Ideal rules	Developmental psych	-0.069	[-0.123, -0.015]	-2.505	0.012
Evolution matters	Developmental psych	-0.018	[-0.072, 0.036]	-0.656	0.512
Computer analogy	Developmental psych	0.032	[-0.022, 0.085]	1.148	0.251
Mind universal	Developmental psych	-0.097	[-0.15, -0.043]	-3.512	<0.001
Social environment	Developmental psych	0.165	[0.112, 0.219]	6.024	<2.08e-04
Perception veridical	Developmental psych	0.010	[-0.044, 0.064]	0.371	0.711
Constructs real	Educational psych	-0.088	[-0.156, -0.02]	-2.539	0.011
Thinking language	Educational psych	0.182	[0.114, 0.25]	5.266	<2.08e-04

Supplementary Table 2: Regression coefficients, research areas *(continued)*

Theme	Research area	Coefficient (b)	CIs	t	p
Math models	Educational psych	-0.049	[-0.117, 0.019]	-1.406	0.160
Wide reach	Educational psych	0.084	[0.016, 0.152]	2.435	0.015
Rational self-interest	Educational psych	0.100	[0.032, 0.167]	2.876	0.004
Neurobiology essential	Educational psych	-0.080	[-0.148, -0.012]	-2.315	0.021
Holistic view	Educational psych	0.058	[-0.01, 0.126]	1.674	0.094
Personality stable	Educational psych	-0.090	[-0.158, -0.022]	-2.605	0.009
Context matters	Educational psych	0.075	[0.007, 0.143]	2.175	0.030
Capacities innate	Educational psych	-0.200	[-0.268, -0.133]	-5.796	<2.08e-04
Ideal rules	Educational psych	-0.077	[-0.145, -0.009]	-2.228	0.026
Evolution matters	Educational psych	0.013	[-0.055, 0.081]	0.373	0.710
Computer analogy	Educational psych	0.061	[-0.006, 0.129]	1.774	0.076
Mind universal	Educational psych	-0.155	[-0.223, -0.087]	-4.472	<2.08e-04
Social environment	Educational psych	0.145	[0.078, 0.213]	4.202	<2.08e-04
Perception veridical	Educational psych	0.007	[-0.061, 0.075]	0.203	0.839
Constructs real	Evolutionary psych	0.272	[0.18, 0.365]	5.771	<2.08e-04
Thinking language	Evolutionary psych	-0.201	[-0.294, -0.109]	-4.259	<2.08e-04
Math models	Evolutionary psych	0.051	[-0.041, 0.144]	1.086	0.278
Wide reach	Evolutionary psych	0.117	[0.024, 0.209]	2.466	0.014
Rational self-interest	Evolutionary psych	-0.085	[-0.177, 0.008]	-1.791	0.073
Neurobiology essential	Evolutionary psych	0.251	[0.158, 0.343]	5.310	<2.08e-04
Holistic view	Evolutionary psych	0.042	[-0.051, 0.135]	0.885	0.376
Personality stable	Evolutionary psych	0.157	[0.064, 0.25]	3.318	0.001
Context matters	Evolutionary psych	-0.119	[-0.212, -0.027]	-2.522	0.012
Capacities innate	Evolutionary psych	0.470	[0.378, 0.562]	9.992	<2.08e-04
Ideal rules	Evolutionary psych	0.150	[0.058, 0.243]	3.181	0.001
Evolution matters	Evolutionary psych	1.293	[1.205, 1.381]	28.703	<2.08e-04
Computer analogy	Evolutionary psych	-0.010	[-0.103, 0.082]	-0.217	0.828
Mind universal	Evolutionary psych	0.367	[0.274, 0.459]	7.783	<2.08e-04
Social environment	Evolutionary psych	-0.120	[-0.213, -0.028]	-2.546	0.011
Perception veridical	Evolutionary psych	-0.031	[-0.124, 0.061]	-0.664	0.506
Constructs real	Forensic psych	0.046	[-0.065, 0.157]	0.811	0.418
Thinking language	Forensic psych	-0.021	[-0.132, 0.091]	-0.363	0.716
Math models	Forensic psych	-0.117	[-0.228, -0.006]	-2.062	0.039
Wide reach	Forensic psych	-0.023	[-0.135, 0.088]	-0.414	0.679
Rational self-interest	Forensic psych	0.111	[0, 0.222]	1.954	0.051
Neurobiology essential	Forensic psych	-0.072	[-0.183, 0.04]	-1.262	0.207
Holistic view	Forensic psych	-0.031	[-0.142, 0.08]	-0.547	0.584
Personality stable	Forensic psych	0.084	[-0.027, 0.195]	1.477	0.140
Context matters	Forensic psych	-0.083	[-0.194, 0.028]	-1.463	0.144
Capacities innate	Forensic psych	0.118	[0.007, 0.229]	2.085	0.037
Ideal rules	Forensic psych	-0.115	[-0.226, -0.004]	-2.031	0.042
Evolution matters	Forensic psych	0.046	[-0.065, 0.157]	0.811	0.417
Computer analogy	Forensic psych	0.044	[-0.067, 0.156]	0.781	0.435
Mind universal	Forensic psych	-0.046	[-0.157, 0.065]	-0.806	0.420

Supplementary Table 2: Regression coefficients, research areas *(continued)*

Theme	Research area	Coefficient (b)	CIs	t	p
Social environment	Forensic psych	-0.035	[-0.146, 0.076]	-0.622	0.534
Perception veridical	Forensic psych	-0.158	[-0.27, -0.047]	-2.794	0.005
Constructs real	Neuropsych	0.121	[0.049, 0.192]	3.319	0.001
Thinking language	Neuropsych	-0.073	[-0.144, -0.001]	-2.001	0.045
Math models	Neuropsych	0.088	[0.017, 0.159]	2.420	0.016
Wide reach	Neuropsych	-0.051	[-0.122, 0.02]	-1.405	0.160
Rational self-interest	Neuropsych	0.144	[0.072, 0.215]	3.955	<2.08e-04
Neurobiology essential	Neuropsych	0.551	[0.48, 0.621]	15.369	<2.08e-04
Holistic view	Neuropsych	-0.107	[-0.178, -0.036]	-2.940	0.003
Personality stable	Neuropsych	0.087	[0.016, 0.159]	2.406	0.016
Context matters	Neuropsych	-0.102	[-0.173, -0.031]	-2.806	0.005
Capacities innate	Neuropsych	0.029	[-0.042, 0.1]	0.799	0.424
Ideal rules	Neuropsych	0.081	[0.01, 0.152]	2.233	0.026
Evolution matters	Neuropsych	0.258	[0.187, 0.33]	7.134	<2.08e-04
Computer analogy	Neuropsych	0.017	[-0.054, 0.088]	0.467	0.641
Mind universal	Neuropsych	0.064	[-0.007, 0.136]	1.770	0.077
Social environment	Neuropsych	-0.229	[-0.3, -0.158]	-6.309	<2.08e-04
Perception veridical	Neuropsych	-0.066	[-0.138, 0.005]	-1.828	0.068
Constructs real	Occupational/IO psych	0.105	[0.028, 0.181]	2.675	0.007
Thinking language	Occupational/IO psych	0.085	[0.009, 0.162]	2.181	0.029
Math models	Occupational/IO psych	-0.123	[-0.2, -0.047]	-3.157	0.002
Wide reach	Occupational/IO psych	0.053	[-0.023, 0.13]	1.364	0.173
Rational self-interest	Occupational/IO psych	-0.161	[-0.238, -0.084]	-4.121	<2.08e-04
Neurobiology essential	Occupational/IO psych	-0.174	[-0.25, -0.097]	-4.445	<2.08e-04
Holistic view	Occupational/IO psych	0.084	[0.008, 0.161]	2.161	0.031
Personality stable	Occupational/IO psych	0.063	[-0.014, 0.139]	1.607	0.108
Context matters	Occupational/IO psych	0.071	[-0.006, 0.147]	1.807	0.071
Capacities innate	Occupational/IO psych	-0.065	[-0.142, 0.011]	-1.666	0.096
Ideal rules	Occupational/IO psych	-0.013	[-0.09, 0.063]	-0.341	0.733
Evolution matters	Occupational/IO psych	-0.091	[-0.168, -0.015]	-2.332	0.020
Computer analogy	Occupational/IO psych	-0.033	[-0.11, 0.043]	-0.849	0.396
Mind universal	Occupational/IO psych	-0.050	[-0.127, 0.027]	-1.278	0.201
Social environment	Occupational/IO psych	0.242	[0.165, 0.318]	6.197	<2.08e-04
Perception veridical	Occupational/IO psych	-0.048	[-0.125, 0.029]	-1.231	0.218
Constructs real	Psychometrics	0.140	[0.073, 0.208]	4.084	<2.08e-04
Thinking language	Psychometrics	0.012	[-0.055, 0.08]	0.357	0.721
Math models	Psychometrics	0.332	[0.265, 0.399]	9.696	<2.08e-04
Wide reach	Psychometrics	0.038	[-0.03, 0.105]	1.091	0.275
Rational self-interest	Psychometrics	0.046	[-0.022, 0.113]	1.333	0.182
Neurobiology essential	Psychometrics	0.043	[-0.024, 0.11]	1.249	0.212
Holistic view	Psychometrics	-0.053	[-0.12, 0.015]	-1.535	0.125
Personality stable	Psychometrics	0.127	[0.059, 0.194]	3.687	<0.001
Context matters	Psychometrics	-0.121	[-0.188, -0.054]	-3.518	<0.001
Capacities innate	Psychometrics	0.063	[-0.005, 0.13]	1.822	0.069

Supplementary Table 2: Regression coefficients, research areas *(continued)*

Theme	Research area	Coefficient (b)	CIs	t	p
Ideal rules	Psychometrics	0.087	[0.02, 0.155]	2.540	0.011
Evolution matters	Psychometrics	0.135	[0.068, 0.203]	3.941	<2.08e-04
Computer analogy	Psychometrics	0.080	[0.012, 0.147]	2.323	0.020
Mind universal	Psychometrics	0.033	[-0.034, 0.101]	0.966	0.334
Social environment	Psychometrics	-0.110	[-0.177, -0.042]	-3.191	0.001
Perception veridical	Psychometrics	0.035	[-0.033, 0.102]	1.009	0.313
Constructs real	Social psych	-0.051	[-0.101, -0.001]	-2.016	0.044
Thinking language	Social psych	0.058	[0.008, 0.108]	2.279	0.023
Math models	Social psych	-0.224	[-0.274, -0.175]	-8.869	<2.08e-04
Wide reach	Social psych	0.022	[-0.027, 0.072]	0.884	0.377
Rational self-interest	Social psych	-0.168	[-0.217, -0.118]	-6.607	<2.08e-04
Neurobiology essential	Social psych	-0.242	[-0.291, -0.192]	-9.560	<2.08e-04
Holistic view	Social psych	0.160	[0.111, 0.21]	6.328	<2.08e-04
Personality stable	Social psych	-0.063	[-0.113, -0.013]	-2.490	0.013
Context matters	Social psych	0.086	[0.036, 0.136]	3.392	0.001
Capacities innate	Social psych	-0.023	[-0.073, 0.027]	-0.909	0.364
Ideal rules	Social psych	-0.041	[-0.091, 0.009]	-1.610	0.107
Evolution matters	Social psych	-0.115	[-0.164, -0.065]	-4.514	<2.08e-04
Computer analogy	Social psych	0.022	[-0.028, 0.072]	0.877	0.380
Mind universal	Social psych	-0.039	[-0.089, 0.011]	-1.524	0.128
Social environment	Social psych	0.369	[0.32, 0.418]	14.706	<2.08e-04
Perception veridical	Social psych	0.018	[-0.032, 0.068]	0.702	0.483
Constructs real	Personality psych	0.191	[0.103, 0.279]	4.272	<2.08e-04
Thinking language	Personality psych	-0.014	[-0.102, 0.074]	-0.317	0.751
Math models	Personality psych	0.016	[-0.072, 0.104]	0.359	0.720
Wide reach	Personality psych	0.099	[0.011, 0.187]	2.210	0.027
Rational self-interest	Personality psych	0.001	[-0.087, 0.089]	0.024	0.981
Neurobiology essential	Personality psych	-0.087	[-0.175, 0.001]	-1.939	0.053
Holistic view	Personality psych	0.064	[-0.024, 0.152]	1.434	0.152
Personality stable	Personality psych	0.300	[0.212, 0.387]	6.706	<2.08e-04
Context matters	Personality psych	-0.148	[-0.236, -0.06]	-3.307	0.001
Capacities innate	Personality psych	0.212	[0.124, 0.3]	4.738	<2.08e-04
Ideal rules	Personality psych	-0.026	[-0.114, 0.062]	-0.583	0.560
Evolution matters	Personality psych	0.136	[0.048, 0.223]	3.028	0.002
Computer analogy	Personality psych	0.083	[-0.005, 0.171]	1.849	0.064
Mind universal	Personality psych	-0.027	[-0.115, 0.06]	-0.612	0.541
Social environment	Personality psych	0.002	[-0.085, 0.09]	0.055	0.956
Perception veridical	Personality psych	-0.021	[-0.109, 0.067]	-0.474	0.635

Supplementary Table 3: Regression coefficients for controversial themes as a function of research methods (each a separate regression model). Results correspond to Fig. 3b in the main text. Regression coefficients (b) represent logits, with the outcome (theme) standardized. P-values in gray reflect cases that failed to meet the Bonferroni-adjusted significance threshold discussed in the main text ($p < .000142$).

Theme	Research method	Coefficient (b)	CIs	t	p
Constructs real	Behav expt: typical adults	0.206	[0.162, 0.25]	9.248	<1.42e-04
Thinking language	Behav expt: typical adults	-0.115	[-0.159, -0.071]	-5.144	<1.42e-04
Math models	Behav expt: typical adults	0.291	[0.248, 0.335]	13.137	<1.42e-04
Wide reach	Behav expt: typical adults	0.103	[0.059, 0.147]	4.612	<1.42e-04
Rational self-interest	Behav expt: typical adults	-0.151	[-0.195, -0.107]	-6.771	<1.42e-04
Neurobiology essential	Behav expt: typical adults	0.173	[0.13, 0.217]	7.768	<1.42e-04
Holistic view	Behav expt: typical adults	-0.255	[-0.298, -0.211]	-11.463	<1.42e-04
Personality stable	Behav expt: typical adults	0.019	[-0.025, 0.063]	0.866	0.387
Context matters	Behav expt: typical adults	-0.108	[-0.151, -0.064]	-4.812	<1.42e-04
Capacities innate	Behav expt: typical adults	0.129	[0.085, 0.173]	5.772	<1.42e-04
Ideal rules	Behav expt: typical adults	0.265	[0.221, 0.308]	11.926	<1.42e-04
Evolution matters	Behav expt: typical adults	-0.022	[-0.066, 0.022]	-0.969	0.332
Computer analogy	Behav expt: typical adults	0.102	[0.059, 0.146]	4.580	<1.42e-04
Mind universal	Behav expt: typical adults	0.330	[0.287, 0.374]	14.948	<1.42e-04
Social environment	Behav expt: typical adults	-0.413	[-0.456, -0.37]	-18.863	<1.42e-04
Perception veridical	Behav expt: typical adults	0.013	[-0.031, 0.057]	0.569	0.569
Constructs real	Behav expt: children	0.053	[-0.004, 0.111]	1.817	0.069
Thinking language	Behav expt: children	0.028	[-0.029, 0.086]	0.962	0.336
Math models	Behav expt: children	0.053	[-0.004, 0.11]	1.812	0.070
Wide reach	Behav expt: children	0.049	[-0.008, 0.107]	1.685	0.092
Rational self-interest	Behav expt: children	0.128	[0.07, 0.185]	4.365	<1.42e-04
Neurobiology essential	Behav expt: children	0.079	[0.022, 0.136]	2.696	0.007
Holistic view	Behav expt: children	-0.007	[-0.065, 0.05]	-0.244	0.807
Personality stable	Behav expt: children	0.010	[-0.048, 0.067]	0.338	0.735
Context matters	Behav expt: children	0.040	[-0.018, 0.097]	1.353	0.176
Capacities innate	Behav expt: children	-0.100	[-0.158, -0.043]	-3.423	0.001
Ideal rules	Behav expt: children	0.070	[0.013, 0.127]	2.392	0.017
Evolution matters	Behav expt: children	-0.008	[-0.065, 0.05]	-0.261	0.794
Computer analogy	Behav expt: children	0.030	[-0.028, 0.087]	1.020	0.308
Mind universal	Behav expt: children	0.102	[0.045, 0.16]	3.499	<0.001
Social environment	Behav expt: children	-0.123	[-0.18, -0.065]	-4.188	<1.42e-04
Perception veridical	Behav expt: children	0.060	[0.002, 0.117]	2.036	0.042
Constructs real	Behav expt: atypical pop	0.094	[0.039, 0.149]	3.324	0.001
Thinking language	Behav expt: atypical pop	-0.073	[-0.128, -0.017]	-2.573	0.010
Math models	Behav expt: atypical pop	0.196	[0.14, 0.251]	6.934	<1.42e-04
Wide reach	Behav expt: atypical pop	0.071	[0.015, 0.126]	2.508	0.012
Rational self-interest	Behav expt: atypical pop	0.051	[-0.004, 0.107]	1.818	0.069
Neurobiology essential	Behav expt: atypical pop	0.300	[0.244, 0.355]	10.666	<1.42e-04
Holistic view	Behav expt: atypical pop	-0.150	[-0.206, -0.095]	-5.320	<1.42e-04
Personality stable	Behav expt: atypical pop	0.018	[-0.037, 0.074]	0.650	0.515

Supplementary Table 3: Regression coefficients, research methods *(continued)*

Theme	Research method	Coefficient (b)	CIs	t	p
Context matters	Behav expt: atypical pop	-0.057	[-0.113, -0.002]	-2.022	0.043
Capacities innate	Behav expt: atypical pop	0.033	[-0.022, 0.089]	1.174	0.240
Ideal rules	Behav expt: atypical pop	0.062	[0.007, 0.118]	2.201	0.028
Evolution matters	Behav expt: atypical pop	0.069	[0.013, 0.124]	2.434	0.015
Computer analogy	Behav expt: atypical pop	0.044	[-0.012, 0.099]	1.551	0.121
Mind universal	Behav expt: atypical pop	0.086	[0.031, 0.142]	3.060	0.002
Social environment	Behav expt: atypical pop	-0.300	[-0.356, -0.245]	-10.701	<1.42e-04
Perception veridical	Behav expt: atypical pop	-0.033	[-0.089, 0.022]	-1.170	0.242
Constructs real	Behav expt: animals	0.199	[0.104, 0.294]	4.127	<1.42e-04
Thinking language	Behav expt: animals	-0.273	[-0.367, -0.178]	-5.663	<1.42e-04
Math models	Behav expt: animals	0.185	[0.09, 0.279]	3.832	<1.42e-04
Wide reach	Behav expt: animals	0.185	[0.09, 0.279]	3.826	<1.42e-04
Rational self-interest	Behav expt: animals	0.048	[-0.047, 0.142]	0.990	0.322
Neurobiology essential	Behav expt: animals	0.651	[0.558, 0.745]	13.646	<1.42e-04
Holistic view	Behav expt: animals	-0.251	[-0.345, -0.156]	-5.199	<1.42e-04
Personality stable	Behav expt: animals	0.044	[-0.051, 0.138]	0.907	0.364
Context matters	Behav expt: animals	-0.054	[-0.148, 0.041]	-1.116	0.265
Capacities innate	Behav expt: animals	0.097	[0.002, 0.192]	2.008	0.045
Ideal rules	Behav expt: animals	0.224	[0.129, 0.318]	4.640	<1.42e-04
Evolution matters	Behav expt: animals	0.474	[0.38, 0.568]	9.881	<1.42e-04
Computer analogy	Behav expt: animals	-0.172	[-0.266, -0.077]	-3.559	<0.001
Mind universal	Behav expt: animals	0.160	[0.066, 0.255]	3.320	0.001
Social environment	Behav expt: animals	-0.375	[-0.47, -0.281]	-7.805	<1.42e-04
Perception veridical	Behav expt: animals	-0.124	[-0.218, -0.029]	-2.564	0.010
Constructs real	Electrophys (EEG/ERP)	0.164	[0.099, 0.228]	4.967	<1.42e-04
Thinking language	Electrophys (EEG/ERP)	-0.143	[-0.207, -0.078]	-4.337	<1.42e-04
Math models	Electrophys (EEG/ERP)	0.353	[0.288, 0.417]	10.765	<1.42e-04
Wide reach	Electrophys (EEG/ERP)	0.039	[-0.026, 0.104]	1.185	0.236
Rational self-interest	Electrophys (EEG/ERP)	0.005	[-0.06, 0.069]	0.139	0.889
Neurobiology essential	Electrophys (EEG/ERP)	0.580	[0.517, 0.643]	17.933	<1.42e-04
Holistic view	Electrophys (EEG/ERP)	-0.119	[-0.184, -0.055]	-3.614	<0.001
Personality stable	Electrophys (EEG/ERP)	0.026	[-0.038, 0.091]	0.796	0.426
Context matters	Electrophys (EEG/ERP)	-0.079	[-0.144, -0.014]	-2.399	0.016
Capacities innate	Electrophys (EEG/ERP)	0.030	[-0.035, 0.094]	0.903	0.367
Ideal rules	Electrophys (EEG/ERP)	0.150	[0.085, 0.214]	4.538	<1.42e-04
Evolution matters	Electrophys (EEG/ERP)	0.168	[0.103, 0.232]	5.087	<1.42e-04
Computer analogy	Electrophys (EEG/ERP)	-0.027	[-0.092, 0.038]	-0.817	0.414
Mind universal	Electrophys (EEG/ERP)	0.230	[0.166, 0.295]	7.003	<1.42e-04
Social environment	Electrophys (EEG/ERP)	-0.527	[-0.59, -0.463]	-16.227	<1.42e-04
Perception veridical	Electrophys (EEG/ERP)	-0.085	[-0.15, -0.021]	-2.586	0.010
Constructs real	Electrophys (single cell)	0.238	[0.054, 0.423]	2.540	0.011
Thinking language	Electrophys (single cell)	-0.316	[-0.5, -0.132]	-3.367	0.001
Math models	Electrophys (single cell)	0.461	[0.277, 0.644]	4.910	<1.42e-04
Wide reach	Electrophys (single cell)	0.192	[0.008, 0.377]	2.049	0.040

Supplementary Table 3: Regression coefficients, research methods *(continued)*

Theme	Research method	Coefficient (b)	CIs	t	p
Rational self-interest	Electrophys (single cell)	-0.040	[-0.224, 0.144]	-0.428	0.669
Neurobiology essential	Electrophys (single cell)	1.163	[0.98, 1.345]	12.497	<1.42e-04
Holistic view	Electrophys (single cell)	-0.237	[-0.421, -0.053]	-2.521	0.012
Personality stable	Electrophys (single cell)	-0.235	[-0.419, -0.051]	-2.504	0.012
Context matters	Electrophys (single cell)	0.024	[-0.16, 0.208]	0.257	0.797
Capacities innate	Electrophys (single cell)	0.044	[-0.14, 0.228]	0.470	0.638
Ideal rules	Electrophys (single cell)	0.092	[-0.092, 0.276]	0.980	0.327
Evolution matters	Electrophys (single cell)	0.312	[0.128, 0.496]	3.325	0.001
Computer analogy	Electrophys (single cell)	-0.299	[-0.483, -0.115]	-3.184	0.001
Mind universal	Electrophys (single cell)	0.085	[-0.1, 0.269]	0.900	0.368
Social environment	Electrophys (single cell)	-0.492	[-0.676, -0.308]	-5.250	<1.42e-04
Perception veridical	Electrophys (single cell)	-0.149	[-0.333, 0.036]	-1.582	0.114
Constructs real	Neuroimaging	0.047	[-0.018, 0.112]	1.427	0.154
Thinking language	Neuroimaging	-0.123	[-0.187, -0.058]	-3.716	<0.001
Math models	Neuroimaging	0.308	[0.243, 0.372]	9.370	<1.42e-04
Wide reach	Neuroimaging	0.029	[-0.035, 0.094]	0.892	0.373
Rational self-interest	Neuroimaging	-0.015	[-0.08, 0.049]	-0.468	0.639
Neurobiology essential	Neuroimaging	0.622	[0.559, 0.686]	19.288	<1.42e-04
Holistic view	Neuroimaging	-0.101	[-0.166, -0.036]	-3.066	0.002
Personality stable	Neuroimaging	0.036	[-0.028, 0.101]	1.104	0.270
Context matters	Neuroimaging	-0.105	[-0.17, -0.04]	-3.179	0.001
Capacities innate	Neuroimaging	0.065	[0, 0.13]	1.972	0.049
Ideal rules	Neuroimaging	0.126	[0.061, 0.191]	3.825	<1.42e-04
Evolution matters	Neuroimaging	0.107	[0.042, 0.171]	3.236	0.001
Computer analogy	Neuroimaging	-0.051	[-0.116, 0.013]	-1.559	0.119
Mind universal	Neuroimaging	0.183	[0.119, 0.248]	5.559	<1.42e-04
Social environment	Neuroimaging	-0.515	[-0.579, -0.452]	-15.856	<1.42e-04
Perception veridical	Neuroimaging	-0.016	[-0.081, 0.049]	-0.488	0.626
Constructs real	Observational Studies	-0.102	[-0.148, -0.056]	-4.316	<1.42e-04
Thinking language	Observational Studies	0.072	[0.026, 0.119]	3.054	0.002
Math models	Observational Studies	-0.168	[-0.214, -0.122]	-7.121	<1.42e-04
Wide reach	Observational Studies	-0.006	[-0.053, 0.04]	-0.263	0.792
Rational self-interest	Observational Studies	0.023	[-0.023, 0.07]	0.980	0.327
Neurobiology essential	Observational Studies	-0.176	[-0.222, -0.13]	-7.460	<1.42e-04
Holistic view	Observational Studies	0.205	[0.159, 0.251]	8.709	<1.42e-04
Personality stable	Observational Studies	-0.042	[-0.089, 0.004]	-1.793	0.073
Context matters	Observational Studies	0.131	[0.085, 0.177]	5.549	<1.42e-04
Capacities innate	Observational Studies	-0.074	[-0.12, -0.028]	-3.127	0.002
Ideal rules	Observational Studies	-0.149	[-0.195, -0.102]	-6.299	<1.42e-04
Evolution matters	Observational Studies	0.049	[0.003, 0.096]	2.092	0.036
Computer analogy	Observational Studies	-0.027	[-0.073, 0.02]	-1.131	0.258
Mind universal	Observational Studies	-0.107	[-0.153, -0.06]	-4.510	<1.42e-04
Social environment	Observational Studies	0.299	[0.253, 0.345]	12.768	<1.42e-04
Perception veridical	Observational Studies	-0.003	[-0.05, 0.043]	-0.139	0.889

Supplementary Table 3: Regression coefficients, research methods *(continued)*

Theme	Research method	Coefficient (b)	CIs	t	p
Constructs real	Comp ling/corpus analysis	-0.083	[-0.173, 0.007]	-1.817	0.069
Thinking language	Comp ling/corpus analysis	0.185	[0.096, 0.275]	4.050	<1.42e-04
Math models	Comp ling/corpus analysis	0.287	[0.197, 0.376]	6.274	<1.42e-04
Wide reach	Comp ling/corpus analysis	-0.015	[-0.105, 0.075]	-0.325	0.745
Rational self-interest	Comp ling/corpus analysis	-0.133	[-0.223, -0.043]	-2.902	0.004
Neurobiology essential	Comp ling/corpus analysis	-0.067	[-0.157, 0.023]	-1.466	0.143
Holistic view	Comp ling/corpus analysis	-0.044	[-0.133, 0.046]	-0.953	0.341
Personality stable	Comp ling/corpus analysis	-0.202	[-0.291, -0.112]	-4.409	<1.42e-04
Context matters	Comp ling/corpus analysis	0.078	[-0.012, 0.168]	1.703	0.089
Capacities innate	Comp ling/corpus analysis	-0.041	[-0.13, 0.049]	-0.887	0.375
Ideal rules	Comp ling/corpus analysis	0.190	[0.1, 0.28]	4.153	<1.42e-04
Evolution matters	Comp ling/corpus analysis	-0.051	[-0.14, 0.039]	-1.107	0.269
Computer analogy	Comp ling/corpus analysis	0.082	[-0.007, 0.172]	1.798	0.072
Mind universal	Comp ling/corpus analysis	0.177	[0.088, 0.267]	3.877	<1.42e-04
Social environment	Comp ling/corpus analysis	-0.235	[-0.325, -0.146]	-5.146	<1.42e-04
Perception veridical	Comp ling/corpus analysis	-0.033	[-0.123, 0.057]	-0.715	0.474
Constructs real	Comp/math modeling	0.063	[0.005, 0.121]	2.144	0.032
Thinking language	Comp/math modeling	-0.101	[-0.158, -0.043]	-3.431	0.001
Math models	Comp/math modeling	0.723	[0.668, 0.778]	25.595	<1.42e-04
Wide reach	Comp/math modeling	0.088	[0.03, 0.145]	2.991	0.003
Rational self-interest	Comp/math modeling	-0.103	[-0.161, -0.045]	-3.507	<0.001
Neurobiology essential	Comp/math modeling	0.132	[0.074, 0.189]	4.492	<1.42e-04
Holistic view	Comp/math modeling	-0.135	[-0.192, -0.077]	-4.591	<1.42e-04
Personality stable	Comp/math modeling	-0.083	[-0.14, -0.025]	-2.810	0.005
Context matters	Comp/math modeling	-0.025	[-0.082, 0.033]	-0.840	0.401
Capacities innate	Comp/math modeling	0.063	[0.005, 0.121]	2.144	0.032
Ideal rules	Comp/math modeling	0.203	[0.145, 0.26]	6.918	<1.42e-04
Evolution matters	Comp/math modeling	-0.015	[-0.073, 0.043]	-0.510	0.610
Computer analogy	Comp/math modeling	-0.003	[-0.061, 0.055]	-0.102	0.919
Mind universal	Comp/math modeling	0.219	[0.162, 0.277]	7.493	<1.42e-04
Social environment	Comp/math modeling	-0.398	[-0.455, -0.341]	-13.686	<1.42e-04
Perception veridical	Comp/math modeling	0.049	[-0.008, 0.107]	1.674	0.094
Constructs real	Clin intervention	-0.077	[-0.132, -0.021]	-2.715	0.007
Thinking language	Clin intervention	0.022	[-0.033, 0.078]	0.789	0.430
Math models	Clin intervention	-0.103	[-0.158, -0.047]	-3.626	<0.001
Wide reach	Clin intervention	-0.144	[-0.2, -0.089]	-5.102	<1.42e-04
Rational self-interest	Clin intervention	0.173	[0.118, 0.229]	6.132	<1.42e-04
Neurobiology essential	Clin intervention	-0.081	[-0.136, -0.025]	-2.844	0.004
Holistic view	Clin intervention	0.158	[0.102, 0.213]	5.579	<1.42e-04
Personality stable	Clin intervention	-0.001	[-0.057, 0.054]	-0.045	0.964
Context matters	Clin intervention	0.037	[-0.019, 0.093]	1.305	0.192
Capacities innate	Clin intervention	-0.052	[-0.108, 0.003]	-1.851	0.064
Ideal rules	Clin intervention	-0.149	[-0.204, -0.093]	-5.255	<1.42e-04
Evolution matters	Clin intervention	0.070	[0.014, 0.125]	2.459	0.014

Supplementary Table 3: Regression coefficients, research methods *(continued)*

Theme	Research method	Coefficient (b)	CIs	t	p
Computer analogy	Clin intervention	-0.011	[-0.067, 0.044]	-0.392	0.695
Mind universal	Clin intervention	-0.184	[-0.24, -0.129]	-6.527	<1.42e-04
Social environment	Clin intervention	0.232	[0.176, 0.287]	8.205	<1.42e-04
Perception veridical	Clin intervention	-0.103	[-0.159, -0.048]	-3.645	<0.001
Constructs real	Case study	-0.305	[-0.359, -0.25]	-10.960	<1.42e-04
Thinking language	Case study	0.111	[0.056, 0.165]	3.950	<1.42e-04
Math models	Case study	-0.360	[-0.415, -0.306]	-12.993	<1.42e-04
Wide reach	Case study	-0.127	[-0.182, -0.073]	-4.554	<1.42e-04
Rational self-interest	Case study	0.031	[-0.024, 0.086]	1.100	0.271
Neurobiology essential	Case study	-0.213	[-0.267, -0.158]	-7.618	<1.42e-04
Holistic view	Case study	0.230	[0.176, 0.285]	8.259	<1.42e-04
Personality stable	Case study	-0.235	[-0.289, -0.18]	-8.414	<1.42e-04
Context matters	Case study	0.182	[0.127, 0.237]	6.519	<1.42e-04
Capacities innate	Case study	-0.188	[-0.243, -0.133]	-6.719	<1.42e-04
Ideal rules	Case study	-0.272	[-0.327, -0.218]	-9.773	<1.42e-04
Evolution matters	Case study	0.063	[0.008, 0.118]	2.244	0.025
Computer analogy	Case study	-0.153	[-0.208, -0.098]	-5.473	<1.42e-04
Mind universal	Case study	-0.299	[-0.354, -0.245]	-10.753	<1.42e-04
Social environment	Case study	0.348	[0.294, 0.402]	12.539	<1.42e-04
Perception veridical	Case study	-0.032	[-0.087, 0.023]	-1.147	0.252
Constructs real	Surveys	0.006	[-0.038, 0.051]	0.285	0.776
Thinking language	Surveys	0.016	[-0.028, 0.06]	0.710	0.478
Math models	Surveys	-0.168	[-0.213, -0.124]	-7.455	<1.42e-04
Wide reach	Surveys	-0.007	[-0.051, 0.037]	-0.306	0.760
Rational self-interest	Surveys	0.055	[0.01, 0.099]	2.416	0.016
Neurobiology essential	Surveys	-0.257	[-0.301, -0.212]	-11.413	<1.42e-04
Holistic view	Surveys	0.151	[0.106, 0.195]	6.665	<1.42e-04
Personality stable	Surveys	0.048	[0.004, 0.092]	2.122	0.034
Context matters	Surveys	0.015	[-0.03, 0.059]	0.641	0.522
Capacities innate	Surveys	0.032	[-0.013, 0.076]	1.402	0.161
Ideal rules	Surveys	-0.057	[-0.101, -0.013]	-2.519	0.012
Evolution matters	Surveys	-0.180	[-0.225, -0.136]	-7.994	<1.42e-04
Computer analogy	Surveys	0.108	[0.064, 0.153]	4.790	<1.42e-04
Mind universal	Surveys	-0.120	[-0.164, -0.075]	-5.285	<1.42e-04
Social environment	Surveys	0.389	[0.345, 0.433]	17.495	<1.42e-04
Perception veridical	Surveys	-0.002	[-0.046, 0.043]	-0.075	0.941
Constructs real	Meta-analyses	0.014	[-0.043, 0.071]	0.490	0.624
Thinking language	Meta-analyses	0.028	[-0.028, 0.085]	0.976	0.329
Math models	Meta-analyses	0.074	[0.017, 0.131]	2.555	0.011
Wide reach	Meta-analyses	0.030	[-0.026, 0.087]	1.053	0.292
Rational self-interest	Meta-analyses	-0.011	[-0.068, 0.046]	-0.387	0.699
Neurobiology essential	Meta-analyses	-0.071	[-0.128, -0.014]	-2.459	0.014
Holistic view	Meta-analyses	0.066	[0.01, 0.123]	2.293	0.022
Personality stable	Meta-analyses	0.064	[0.007, 0.12]	2.195	0.028

Supplementary Table 3: Regression coefficients, research methods *(continued)*

Theme	Research method	Coefficient (b)	CIs	t	p
Context matters	Meta-analyses	-0.010	[-0.067, 0.047]	-0.341	0.733
Capacities innate	Meta-analyses	-0.017	[-0.074, 0.039]	-0.595	0.552
Ideal rules	Meta-analyses	-0.016	[-0.073, 0.04]	-0.564	0.572
Evolution matters	Meta-analyses	0.002	[-0.055, 0.059]	0.072	0.943
Computer analogy	Meta-analyses	0.026	[-0.03, 0.083]	0.910	0.363
Mind universal	Meta-analyses	-0.025	[-0.081, 0.032]	-0.849	0.396
Social environment	Meta-analyses	0.048	[-0.009, 0.105]	1.659	0.097
Perception veridical	Meta-analyses	0.026	[-0.031, 0.083]	0.898	0.369
Constructs real	Pharm intervention	0.053	[-0.05, 0.156]	1.008	0.314
Thinking language	Pharm intervention	-0.210	[-0.313, -0.107]	-4.007	<1.42e-04
Math models	Pharm intervention	0.079	[-0.024, 0.182]	1.503	0.133
Wide reach	Pharm intervention	-0.025	[-0.128, 0.078]	-0.477	0.633
Rational self-interest	Pharm intervention	0.163	[0.06, 0.265]	3.106	0.002
Neurobiology essential	Pharm intervention	0.569	[0.467, 0.671]	10.927	<1.42e-04
Holistic view	Pharm intervention	-0.142	[-0.245, -0.039]	-2.706	0.007
Personality stable	Pharm intervention	0.093	[-0.01, 0.196]	1.772	0.076
Context matters	Pharm intervention	-0.156	[-0.259, -0.053]	-2.973	0.003
Capacities innate	Pharm intervention	0.077	[-0.026, 0.18]	1.473	0.141
Ideal rules	Pharm intervention	0.076	[-0.027, 0.179]	1.446	0.148
Evolution matters	Pharm intervention	0.163	[0.06, 0.266]	3.114	0.002
Computer analogy	Pharm intervention	-0.109	[-0.212, -0.006]	-2.078	0.038
Mind universal	Pharm intervention	-0.033	[-0.136, 0.07]	-0.631	0.528
Social environment	Pharm intervention	-0.270	[-0.373, -0.168]	-5.165	<1.42e-04
Perception veridical	Pharm intervention	-0.138	[-0.24, -0.035]	-2.628	0.009
Constructs real	Eye-tracking	0.078	[0.014, 0.142]	2.399	0.016
Thinking language	Eye-tracking	-0.096	[-0.16, -0.033]	-2.967	0.003
Math models	Eye-tracking	0.340	[0.277, 0.403]	10.519	<1.42e-04
Wide reach	Eye-tracking	0.065	[0.002, 0.129]	2.008	0.045
Rational self-interest	Eye-tracking	-0.075	[-0.139, -0.011]	-2.303	0.021
Neurobiology essential	Eye-tracking	0.295	[0.232, 0.359]	9.123	<1.42e-04
Holistic view	Eye-tracking	-0.211	[-0.275, -0.148]	-6.514	<1.42e-04
Personality stable	Eye-tracking	-0.053	[-0.117, 0.011]	-1.624	0.104
Context matters	Eye-tracking	-0.062	[-0.126, 0.001]	-1.916	0.055
Capacities innate	Eye-tracking	0.034	[-0.03, 0.097]	1.034	0.301
Ideal rules	Eye-tracking	0.184	[0.121, 0.248]	5.681	<1.42e-04
Evolution matters	Eye-tracking	0.001	[-0.063, 0.065]	0.037	0.971
Computer analogy	Eye-tracking	0.044	[-0.02, 0.108]	1.360	0.174
Mind universal	Eye-tracking	0.309	[0.246, 0.373]	9.566	<1.42e-04
Social environment	Eye-tracking	-0.508	[-0.571, -0.445]	-15.857	<1.42e-04
Perception veridical	Eye-tracking	0.000	[-0.064, 0.064]	-0.008	0.993
Constructs real	Cranial stim (e.g. TMS; tDCS)	0.085	[-0.034, 0.203]	1.402	0.161
Thinking language	Cranial stim (e.g. TMS; tDCS)	-0.220	[-0.339, -0.102]	-3.651	<0.001
Math models	Cranial stim (e.g. TMS; tDCS)	0.301	[0.183, 0.419]	4.985	<1.42e-04
Wide reach	Cranial stim (e.g. TMS; tDCS)	-0.006	[-0.125, 0.112]	-0.100	0.920

Supplementary Table 3: Regression coefficients, research methods *(continued)*

Theme	Research method	Coefficient (b)	CIs	t	p
Rational self-interest	Cranial stim (e.g. TMS; tDCS)	-0.080	[-0.199, 0.038]	-1.329	0.184
Neurobiology essential	Cranial stim (e.g. TMS; tDCS)	0.748	[0.63, 0.865]	12.496	<1.42e-04
Holistic view	Cranial stim (e.g. TMS; tDCS)	-0.201	[-0.319, -0.083]	-3.328	0.001
Personality stable	Cranial stim (e.g. TMS; tDCS)	0.074	[-0.045, 0.192]	1.219	0.223
Context matters	Cranial stim (e.g. TMS; tDCS)	-0.113	[-0.232, 0.005]	-1.877	0.061
Capacities innate	Cranial stim (e.g. TMS; tDCS)	0.099	[-0.019, 0.218]	1.645	0.100
Ideal rules	Cranial stim (e.g. TMS; tDCS)	0.141	[0.022, 0.259]	2.329	0.020
Evolution matters	Cranial stim (e.g. TMS; tDCS)	0.077	[-0.042, 0.195]	1.267	0.205
Computer analogy	Cranial stim (e.g. TMS; tDCS)	-0.130	[-0.248, -0.012]	-2.151	0.032
Mind universal	Cranial stim (e.g. TMS; tDCS)	0.272	[0.154, 0.391]	4.513	<1.42e-04
Social environment	Cranial stim (e.g. TMS; tDCS)	-0.720	[-0.838, -0.603]	-12.031	<1.42e-04
Perception veridical	Cranial stim (e.g. TMS; tDCS)	-0.102	[-0.22, 0.017]	-1.686	0.092
Constructs real	Interviews	-0.228	[-0.273, -0.184]	-10.032	<1.42e-04
Thinking language	Interviews	0.116	[0.071, 0.161]	5.081	<1.42e-04
Math models	Interviews	-0.368	[-0.412, -0.324]	-16.312	<1.42e-04
Wide reach	Interviews	-0.072	[-0.117, -0.027]	-3.137	0.002
Rational self-interest	Interviews	0.053	[0.008, 0.098]	2.316	0.021
Neurobiology essential	Interviews	-0.336	[-0.38, -0.292]	-14.857	<1.42e-04
Holistic view	Interviews	0.320	[0.275, 0.364]	14.121	<1.42e-04
Personality stable	Interviews	-0.065	[-0.11, -0.02]	-2.835	0.005
Context matters	Interviews	0.154	[0.109, 0.198]	6.723	<1.42e-04
Capacities innate	Interviews	-0.116	[-0.16, -0.071]	-5.055	<1.42e-04
Ideal rules	Interviews	-0.260	[-0.304, -0.215]	-11.436	<1.42e-04
Evolution matters	Interviews	-0.151	[-0.195, -0.106]	-6.591	<1.42e-04
Computer analogy	Interviews	-0.076	[-0.121, -0.031]	-3.314	0.001
Mind universal	Interviews	-0.263	[-0.307, -0.218]	-11.552	<1.42e-04
Social environment	Interviews	0.505	[0.461, 0.548]	22.733	<1.42e-04
Perception veridical	Interviews	-0.006	[-0.051, 0.039]	-0.258	0.796
Constructs real	Longitudinal studies	-0.021	[-0.07, 0.027]	-0.868	0.386
Thinking language	Longitudinal studies	0.051	[0.002, 0.099]	2.061	0.039
Math models	Longitudinal studies	-0.027	[-0.076, 0.021]	-1.113	0.266
Wide reach	Longitudinal studies	-0.045	[-0.093, 0.004]	-1.814	0.070
Rational self-interest	Longitudinal studies	0.048	[0, 0.096]	1.965	0.049
Neurobiology essential	Longitudinal studies	-0.106	[-0.154, -0.058]	-4.306	<1.42e-04
Holistic view	Longitudinal studies	0.197	[0.149, 0.245]	8.041	<1.42e-04
Personality stable	Longitudinal studies	0.066	[0.018, 0.114]	2.692	0.007
Context matters	Longitudinal studies	0.018	[-0.03, 0.066]	0.738	0.460
Capacities innate	Longitudinal studies	-0.070	[-0.118, -0.021]	-2.831	0.005
Ideal rules	Longitudinal studies	-0.135	[-0.184, -0.087]	-5.519	<1.42e-04
Evolution matters	Longitudinal studies	-0.096	[-0.144, -0.048]	-3.919	<1.42e-04
Computer analogy	Longitudinal studies	-0.001	[-0.049, 0.047]	-0.044	0.965
Mind universal	Longitudinal studies	-0.098	[-0.147, -0.05]	-4.006	<1.42e-04
Social environment	Longitudinal studies	0.187	[0.139, 0.235]	7.636	<1.42e-04
Perception veridical	Longitudinal studies	-0.045	[-0.093, 0.003]	-1.842	0.066

Supplementary Table 3: Regression coefficients, research methods *(continued)*

Theme	Research method	Coefficient (b)	CIs	t	p
Constructs real	Behavioral intervention	-0.015	[-0.067, 0.037]	-0.561	0.575
Thinking language	Behavioral intervention	0.023	[-0.029, 0.075]	0.859	0.390
Math models	Behavioral intervention	-0.035	[-0.087, 0.017]	-1.310	0.190
Wide reach	Behavioral intervention	0.012	[-0.04, 0.064]	0.444	0.657
Rational self-interest	Behavioral intervention	0.138	[0.086, 0.191]	5.199	<1.42e-04
Neurobiology essential	Behavioral intervention	-0.067	[-0.12, -0.015]	-2.521	0.012
Holistic view	Behavioral intervention	0.108	[0.056, 0.161]	4.063	<1.42e-04
Personality stable	Behavioral intervention	-0.002	[-0.054, 0.05]	-0.071	0.944
Context matters	Behavioral intervention	0.026	[-0.027, 0.078]	0.964	0.335
Capacities innate	Behavioral intervention	-0.121	[-0.173, -0.069]	-4.539	<1.42e-04
Ideal rules	Behavioral intervention	-0.057	[-0.109, -0.005]	-2.130	0.033
Evolution matters	Behavioral intervention	-0.049	[-0.102, 0.003]	-1.855	0.064
Computer analogy	Behavioral intervention	0.012	[-0.04, 0.064]	0.454	0.650
Mind universal	Behavioral intervention	-0.111	[-0.163, -0.059]	-4.163	<1.42e-04
Social environment	Behavioral intervention	0.193	[0.14, 0.245]	7.242	<1.42e-04
Perception veridical	Behavioral intervention	-0.052	[-0.104, 0.001]	-1.936	0.053
Constructs real	Twin studies	0.217	[0.052, 0.381]	2.587	0.010
Thinking language	Twin studies	-0.169	[-0.333, -0.004]	-2.012	0.044
Math models	Twin studies	0.188	[0.024, 0.353]	2.247	0.025
Wide reach	Twin studies	0.041	[-0.124, 0.205]	0.486	0.627
Rational self-interest	Twin studies	0.049	[-0.116, 0.213]	0.582	0.561
Neurobiology essential	Twin studies	0.105	[-0.06, 0.269]	1.249	0.212
Holistic view	Twin studies	0.054	[-0.11, 0.218]	0.646	0.518
Personality stable	Twin studies	0.348	[0.184, 0.512]	4.160	<1.42e-04
Context matters	Twin studies	-0.314	[-0.478, -0.15]	-3.751	<0.001
Capacities innate	Twin studies	0.350	[0.186, 0.515]	4.186	<1.42e-04
Ideal rules	Twin studies	-0.026	[-0.191, 0.138]	-0.314	0.754
Evolution matters	Twin studies	0.351	[0.186, 0.515]	4.187	<1.42e-04
Computer analogy	Twin studies	0.136	[-0.028, 0.301]	1.626	0.104
Mind universal	Twin studies	0.013	[-0.151, 0.178]	0.158	0.875
Social environment	Twin studies	-0.356	[-0.52, -0.192]	-4.248	<1.42e-04
Perception veridical	Twin studies	0.105	[-0.059, 0.27]	1.257	0.209
Constructs real	Self-report/guided writing	-0.039	[-0.097, 0.019]	-1.313	0.189
Thinking language	Self-report/guided writing	0.094	[0.036, 0.152]	3.158	0.002
Math models	Self-report/guided writing	-0.115	[-0.173, -0.057]	-3.875	<1.42e-04
Wide reach	Self-report/guided writing	-0.079	[-0.137, -0.021]	-2.660	0.008
Rational self-interest	Self-report/guided writing	-0.014	[-0.073, 0.044]	-0.485	0.628
Neurobiology essential	Self-report/guided writing	-0.186	[-0.244, -0.128]	-6.277	<1.42e-04
Holistic view	Self-report/guided writing	0.165	[0.107, 0.223]	5.568	<1.42e-04
Personality stable	Self-report/guided writing	-0.025	[-0.084, 0.033]	-0.857	0.391
Context matters	Self-report/guided writing	0.087	[0.029, 0.145]	2.928	0.003
Capacities innate	Self-report/guided writing	-0.026	[-0.084, 0.032]	-0.872	0.383
Ideal rules	Self-report/guided writing	-0.149	[-0.207, -0.091]	-5.037	<1.42e-04
Evolution matters	Self-report/guided writing	-0.093	[-0.151, -0.035]	-3.129	0.002

Supplementary Table 3: Regression coefficients, research methods *(continued)*

Theme	Research method	Coefficient (b)	CIs	t	p
Computer analogy	Self-report/guided writing	0.049	[-0.009, 0.107]	1.642	0.101
Mind universal	Self-report/guided writing	-0.160	[-0.218, -0.102]	-5.398	<1.42e-04
Social environment	Self-report/guided writing	0.288	[0.231, 0.346]	9.782	<1.42e-04
Perception veridical	Self-report/guided writing	-0.059	[-0.117, -0.001]	-2.002	0.045

Supplementary Table 4: Regression coefficients for controversial themes as a function of cognitive traits (each a separate regression model). Results correspond to Fig. 3c in the main text, with a subset also shown in Fig. 2. Regression coefficients (β) are equivalent to Pearson correlations, as both outcome and predictor have been standardized. P-values in gray reflect cases that failed to meet the Bonferroni-adjusted significance threshold discussed in the main text ($p < .000223$).

Theme	Cognitive trait	Coefficient (β)	CIs	t	p
Constructs real	Analytic	0.053	[0.031, 0.075]	4.718	<2.23e-04
Thinking language	Analytic	-0.018	[-0.04, 0.004]	-1.586	0.113
Math models	Analytic	0.126	[0.104, 0.148]	11.342	<2.23e-04
Wide reach	Analytic	0.046	[0.024, 0.068]	4.149	<2.23e-04
Rational self-interest	Analytic	-0.002	[-0.024, 0.02]	-0.148	0.882
Neurobiology essential	Analytic	0.007	[-0.015, 0.029]	0.653	0.513
Holistic view	Analytic	-0.061	[-0.083, -0.039]	-5.422	<2.23e-04
Personality stable	Analytic	0.035	[0.013, 0.056]	3.087	0.002
Context matters	Analytic	-0.042	[-0.064, -0.02]	-3.786	<2.23e-04
Capacities innate	Analytic	0.050	[0.028, 0.072]	4.433	<2.23e-04
Ideal rules	Analytic	0.051	[0.029, 0.073]	4.560	<2.23e-04
Evolution matters	Analytic	-0.030	[-0.052, -0.008]	-2.694	0.007
Computer analogy	Analytic	0.031	[0.009, 0.053]	2.781	0.005
Mind universal	Analytic	0.073	[0.051, 0.095]	6.508	<2.23e-04
Social environment	Analytic	-0.072	[-0.094, -0.05]	-6.478	<2.23e-04
Perception veridical	Analytic	0.035	[0.013, 0.057]	3.165	0.002
Constructs real	Creative	-0.029	[-0.051, -0.008]	-2.635	0.008
Thinking language	Creative	0.005	[-0.017, 0.027]	0.439	0.661
Math models	Creative	-0.061	[-0.083, -0.039]	-5.469	<2.23e-04
Wide reach	Creative	0.010	[-0.012, 0.032]	0.868	0.386
Rational self-interest	Creative	-0.039	[-0.06, -0.017]	-3.445	0.001
Neurobiology essential	Creative	0.001	[-0.021, 0.023]	0.052	0.958
Holistic view	Creative	0.053	[0.031, 0.075]	4.705	<2.23e-04
Personality stable	Creative	-0.078	[-0.1, -0.056]	-6.978	<2.23e-04
Context matters	Creative	0.057	[0.035, 0.079]	5.086	<2.23e-04
Capacities innate	Creative	-0.015	[-0.037, 0.007]	-1.370	0.171
Ideal rules	Creative	-0.029	[-0.051, -0.007]	-2.602	0.009
Evolution matters	Creative	0.057	[0.035, 0.079]	5.133	<2.23e-04
Computer analogy	Creative	-0.072	[-0.094, -0.05]	-6.424	<2.23e-04
Mind universal	Creative	-0.041	[-0.063, -0.019]	-3.683	<0.001
Social environment	Creative	0.029	[0.007, 0.051]	2.576	0.010
Perception veridical	Creative	-0.002	[-0.024, 0.02]	-0.161	0.872
Constructs real	Practical	0.014	[-0.008, 0.036]	1.285	0.199
Thinking language	Practical	-0.031	[-0.053, -0.009]	-2.777	0.006
Math models	Practical	-0.010	[-0.032, 0.012]	-0.923	0.356
Wide reach	Practical	0.024	[0.002, 0.046]	2.132	0.033
Rational self-interest	Practical	0.076	[0.055, 0.098]	6.846	<2.23e-04
Neurobiology essential	Practical	0.015	[-0.007, 0.037]	1.363	0.173
Holistic view	Practical	0.006	[-0.016, 0.028]	0.560	0.575

Supplementary Table 4: Regression coefficients, cognitive traits *(continued)*

Theme	Cognitive trait	Coefficient (β)	CIs	t	p
Personality stable	Practical	-0.003	[-0.025, 0.019]	-0.252	0.801
Context matters	Practical	0.033	[0.011, 0.055]	2.951	0.003
Capacities innate	Practical	-0.042	[-0.064, -0.02]	-3.725	<2.23e-04
Ideal rules	Practical	-0.019	[-0.041, 0.003]	-1.726	0.084
Evolution matters	Practical	-0.011	[-0.033, 0.011]	-0.980	0.327
Computer analogy	Practical	0.000	[-0.022, 0.022]	0.029	0.977
Mind universal	Practical	-0.016	[-0.038, 0.006]	-1.398	0.162
Social environment	Practical	0.029	[0.008, 0.051]	2.635	0.008
Perception veridical	Practical	0.009	[-0.013, 0.031]	0.820	0.412
Constructs real	Deliberation	0.112	[0.09, 0.133]	10.031	<2.23e-04
Thinking language	Deliberation	-0.001	[-0.023, 0.021]	-0.077	0.939
Math models	Deliberation	0.046	[0.024, 0.068]	4.145	<2.23e-04
Wide reach	Deliberation	0.059	[0.037, 0.081]	5.291	<2.23e-04
Rational self-interest	Deliberation	0.106	[0.085, 0.128]	9.552	<2.23e-04
Neurobiology essential	Deliberation	0.041	[0.019, 0.063]	3.664	<0.001
Holistic view	Deliberation	-0.026	[-0.048, -0.004]	-2.282	0.023
Personality stable	Deliberation	0.124	[0.102, 0.146]	11.167	<2.23e-04
Context matters	Deliberation	-0.018	[-0.04, 0.004]	-1.642	0.101
Capacities innate	Deliberation	0.027	[0.006, 0.049]	2.454	0.014
Ideal rules	Deliberation	0.041	[0.019, 0.063]	3.697	<2.23e-04
Evolution matters	Deliberation	-0.037	[-0.059, -0.015]	-3.291	0.001
Computer analogy	Deliberation	0.065	[0.043, 0.087]	5.799	<2.23e-04
Mind universal	Deliberation	0.033	[0.011, 0.055]	2.917	0.004
Social environment	Deliberation	-0.026	[-0.048, -0.005]	-2.365	0.018
Perception veridical	Deliberation	0.012	[-0.01, 0.034]	1.082	0.279
Constructs real	Cognitive structure	0.115	[0.093, 0.137]	10.333	<2.23e-04
Thinking language	Cognitive structure	-0.005	[-0.027, 0.017]	-0.485	0.628
Math models	Cognitive structure	0.071	[0.049, 0.092]	6.313	<2.23e-04
Wide reach	Cognitive structure	0.034	[0.012, 0.056]	3.054	0.002
Rational self-interest	Cognitive structure	0.136	[0.114, 0.158]	12.259	<2.23e-04
Neurobiology essential	Cognitive structure	0.052	[0.03, 0.073]	4.610	<2.23e-04
Holistic view	Cognitive structure	-0.054	[-0.076, -0.032]	-4.796	<2.23e-04
Personality stable	Cognitive structure	0.107	[0.085, 0.129]	9.599	<2.23e-04
Context matters	Cognitive structure	-0.032	[-0.054, -0.01]	-2.878	0.004
Capacities innate	Cognitive structure	0.038	[0.016, 0.06]	3.384	0.001
Ideal rules	Cognitive structure	0.056	[0.034, 0.078]	4.979	<2.23e-04
Evolution matters	Cognitive structure	-0.060	[-0.082, -0.038]	-5.387	<2.23e-04
Computer analogy	Cognitive structure	0.095	[0.073, 0.117]	8.509	<2.23e-04
Mind universal	Cognitive structure	0.042	[0.02, 0.064]	3.792	<2.23e-04
Social environment	Cognitive structure	-0.032	[-0.054, -0.01]	-2.885	0.004
Perception veridical	Cognitive structure	0.034	[0.012, 0.056]	3.064	0.002
Constructs real	Aesthetics	-0.038	[-0.06, -0.016]	-3.400	0.001
Thinking language	Aesthetics	0.025	[0.003, 0.047]	2.201	0.028
Math models	Aesthetics	-0.054	[-0.076, -0.033]	-4.867	<2.23e-04

Supplementary Table 4: Regression coefficients, cognitive traits *(continued)*

Theme	Cognitive trait	Coefficient (β)	CIs	t	p
Wide reach	Aesthetics	-0.001	[-0.023, 0.021]	-0.066	0.947
Rational self-interest	Aesthetics	-0.072	[-0.094, -0.05]	-6.478	<2.23e-04
Neurobiology essential	Aesthetics	-0.010	[-0.032, 0.012]	-0.926	0.355
Holistic view	Aesthetics	0.035	[0.013, 0.057]	3.161	0.002
Personality stable	Aesthetics	-0.072	[-0.094, -0.051]	-6.485	<2.23e-04
Context matters	Aesthetics	0.064	[0.042, 0.086]	5.753	<2.23e-04
Capacities innate	Aesthetics	-0.006	[-0.028, 0.016]	-0.538	0.590
Ideal rules	Aesthetics	-0.047	[-0.069, -0.025]	-4.191	<2.23e-04
Evolution matters	Aesthetics	0.058	[0.036, 0.08]	5.180	<2.23e-04
Computer analogy	Aesthetics	-0.056	[-0.078, -0.034]	-4.976	<2.23e-04
Mind universal	Aesthetics	-0.024	[-0.046, -0.002]	-2.154	0.031
Social environment	Aesthetics	0.072	[0.05, 0.094]	6.470	<2.23e-04
Perception veridical	Aesthetics	-0.009	[-0.031, 0.013]	-0.814	0.416
Constructs real	Breadth of interest	-0.040	[-0.062, -0.018]	-3.557	<0.001
Thinking language	Breadth of interest	0.045	[0.023, 0.067]	4.039	<2.23e-04
Math models	Breadth of interest	-0.064	[-0.085, -0.042]	-5.681	<2.23e-04
Wide reach	Breadth of interest	0.024	[0.002, 0.046]	2.173	0.030
Rational self-interest	Breadth of interest	-0.096	[-0.118, -0.074]	-8.597	<2.23e-04
Neurobiology essential	Breadth of interest	0.001	[-0.021, 0.023]	0.058	0.954
Holistic view	Breadth of interest	0.045	[0.023, 0.067]	4.043	<2.23e-04
Personality stable	Breadth of interest	-0.055	[-0.077, -0.033]	-4.946	<2.23e-04
Context matters	Breadth of interest	0.061	[0.039, 0.083]	5.441	<2.23e-04
Capacities innate	Breadth of interest	0.000	[-0.022, 0.022]	-0.035	0.972
Ideal rules	Breadth of interest	-0.053	[-0.075, -0.031]	-4.768	<2.23e-04
Evolution matters	Breadth of interest	0.063	[0.041, 0.085]	5.628	<2.23e-04
Computer analogy	Breadth of interest	-0.063	[-0.085, -0.041]	-5.633	<2.23e-04
Mind universal	Breadth of interest	-0.018	[-0.04, 0.004]	-1.568	0.117
Social environment	Breadth of interest	0.085	[0.063, 0.107]	7.601	<2.23e-04
Perception veridical	Breadth of interest	-0.032	[-0.054, -0.01]	-2.848	0.004
Constructs real	Dominance	0.003	[-0.019, 0.025]	0.241	0.809
Thinking language	Dominance	-0.014	[-0.036, 0.008]	-1.247	0.212
Math models	Dominance	-0.010	[-0.031, 0.012]	-0.852	0.394
Wide reach	Dominance	0.041	[0.019, 0.063]	3.662	<0.001
Rational self-interest	Dominance	0.074	[0.052, 0.096]	6.653	<2.23e-04
Neurobiology essential	Dominance	0.033	[0.011, 0.055]	2.912	0.004
Holistic view	Dominance	0.034	[0.012, 0.056]	3.022	0.003
Personality stable	Dominance	0.004	[-0.018, 0.026]	0.392	0.695
Context matters	Dominance	0.030	[0.008, 0.052]	2.703	0.007
Capacities innate	Dominance	-0.033	[-0.055, -0.012]	-2.990	0.003
Ideal rules	Dominance	-0.018	[-0.04, 0.004]	-1.589	0.112
Evolution matters	Dominance	-0.002	[-0.023, 0.02]	-0.135	0.893
Computer analogy	Dominance	0.027	[0.005, 0.049]	2.396	0.017
Mind universal	Dominance	-0.023	[-0.045, -0.001]	-2.041	0.041
Social environment	Dominance	0.037	[0.015, 0.059]	3.297	0.001

Supplementary Table 4: Regression coefficients, cognitive traits *(continued)*

Theme	Cognitive trait	Coefficient (β)	CIs	t	p
Perception veridical	Dominance	-0.005	[-0.027, 0.017]	-0.440	0.660
Constructs real	Verbal orientation	-0.019	[-0.041, 0.003]	-1.684	0.092
Thinking language	Verbal orientation	0.064	[0.042, 0.086]	5.706	<2.23e-04
Math models	Verbal orientation	-0.055	[-0.077, -0.033]	-4.893	<2.23e-04
Wide reach	Verbal orientation	0.016	[-0.006, 0.038]	1.449	0.147
Rational self-interest	Verbal orientation	-0.030	[-0.052, -0.008]	-2.652	0.008
Neurobiology essential	Verbal orientation	-0.040	[-0.062, -0.018]	-3.557	<0.001
Holistic view	Verbal orientation	0.057	[0.035, 0.079]	5.083	<2.23e-04
Personality stable	Verbal orientation	0.009	[-0.013, 0.031]	0.797	0.426
Context matters	Verbal orientation	0.026	[0.004, 0.048]	2.340	0.019
Capacities innate	Verbal orientation	0.050	[0.028, 0.072]	4.473	<2.23e-04
Ideal rules	Verbal orientation	-0.038	[-0.06, -0.016]	-3.410	0.001
Evolution matters	Verbal orientation	-0.021	[-0.043, 0.001]	-1.910	0.056
Computer analogy	Verbal orientation	-0.018	[-0.04, 0.004]	-1.644	0.100
Mind universal	Verbal orientation	-0.031	[-0.053, -0.009]	-2.808	0.005
Social environment	Verbal orientation	0.082	[0.06, 0.104]	7.328	<2.23e-04
Perception veridical	Verbal orientation	-0.048	[-0.07, -0.026]	-4.269	<2.23e-04
Constructs real	Visual orientation	0.055	[0.033, 0.077]	4.933	<2.23e-04
Thinking language	Visual orientation	-0.036	[-0.058, -0.015]	-3.258	0.001
Math models	Visual orientation	0.097	[0.075, 0.119]	8.688	<2.23e-04
Wide reach	Visual orientation	0.015	[-0.006, 0.037]	1.382	0.167
Rational self-interest	Visual orientation	0.004	[-0.018, 0.026]	0.379	0.704
Neurobiology essential	Visual orientation	0.053	[0.031, 0.075]	4.761	<2.23e-04
Holistic view	Visual orientation	-0.029	[-0.051, -0.007]	-2.615	0.009
Personality stable	Visual orientation	0.055	[0.033, 0.077]	4.953	<2.23e-04
Context matters	Visual orientation	-0.018	[-0.04, 0.004]	-1.578	0.115
Capacities innate	Visual orientation	0.060	[0.038, 0.082]	5.336	<2.23e-04
Ideal rules	Visual orientation	0.032	[0.01, 0.054]	2.859	0.004
Evolution matters	Visual orientation	0.030	[0.008, 0.052]	2.683	0.007
Computer analogy	Visual orientation	0.020	[-0.002, 0.042]	1.801	0.072
Mind universal	Visual orientation	0.061	[0.039, 0.083]	5.491	<2.23e-04
Social environment	Visual orientation	-0.009	[-0.031, 0.013]	-0.815	0.415
Perception veridical	Visual orientation	0.007	[-0.015, 0.029]	0.656	0.512
Constructs real	Tolerance of Ambiguity	-0.154	[-0.176, -0.132]	-13.926	<2.23e-04
Thinking language	Tolerance of Ambiguity	-0.001	[-0.023, 0.02]	-0.132	0.895
Math models	Tolerance of Ambiguity	-0.123	[-0.145, -0.101]	-11.084	<2.23e-04
Wide reach	Tolerance of Ambiguity	-0.039	[-0.061, -0.017]	-3.464	0.001
Rational self-interest	Tolerance of Ambiguity	-0.181	[-0.202, -0.159]	-16.412	<2.23e-04
Neurobiology essential	Tolerance of Ambiguity	-0.104	[-0.126, -0.082]	-9.332	<2.23e-04
Holistic view	Tolerance of Ambiguity	0.122	[0.1, 0.144]	10.981	<2.23e-04
Personality stable	Tolerance of Ambiguity	-0.097	[-0.118, -0.075]	-8.666	<2.23e-04
Context matters	Tolerance of Ambiguity	0.080	[0.058, 0.102]	7.155	<2.23e-04
Capacities innate	Tolerance of Ambiguity	-0.011	[-0.033, 0.011]	-0.943	0.346
Ideal rules	Tolerance of Ambiguity	-0.098	[-0.12, -0.076]	-8.790	<2.23e-04

Supplementary Table 4: Regression coefficients, cognitive traits *(continued)*

Theme	Cognitive trait	Coefficient (β)	CIs	t	p
Evolution matters	Tolerance of Ambiguity	0.017	[-0.005, 0.039]	1.548	0.122
Computer analogy	Tolerance of Ambiguity	-0.132	[-0.154, -0.11]	-11.865	<2.23e-04
Mind universal	Tolerance of Ambiguity	-0.069	[-0.091, -0.047]	-6.178	<2.23e-04
Social environment	Tolerance of Ambiguity	0.080	[0.058, 0.102]	7.172	<2.23e-04
Perception veridical	Tolerance of Ambiguity	-0.079	[-0.101, -0.058]	-7.113	<2.23e-04
Constructs real	Object imagery	0.019	[-0.003, 0.041]	1.662	0.097
Thinking language	Object imagery	-0.032	[-0.054, -0.01]	-2.870	0.004
Math models	Object imagery	-0.025	[-0.047, -0.003]	-2.241	0.025
Wide reach	Object imagery	0.000	[-0.022, 0.022]	-0.014	0.989
Rational self-interest	Object imagery	0.073	[0.051, 0.095]	6.546	<2.23e-04
Neurobiology essential	Object imagery	0.044	[0.022, 0.066]	3.947	<2.23e-04
Holistic view	Object imagery	0.039	[0.017, 0.06]	3.442	0.001
Personality stable	Object imagery	0.006	[-0.016, 0.028]	0.562	0.574
Context matters	Object imagery	0.017	[-0.005, 0.039]	1.495	0.135
Capacities innate	Object imagery	-0.018	[-0.04, 0.004]	-1.624	0.104
Ideal rules	Object imagery	-0.033	[-0.054, -0.011]	-2.904	0.004
Evolution matters	Object imagery	0.063	[0.041, 0.084]	5.598	<2.23e-04
Computer analogy	Object imagery	0.025	[0.003, 0.047]	2.204	0.028
Mind universal	Object imagery	-0.052	[-0.074, -0.03]	-4.664	<2.23e-04
Social environment	Object imagery	0.075	[0.053, 0.097]	6.727	<2.23e-04
Perception veridical	Object imagery	0.003	[-0.019, 0.025]	0.271	0.786
Constructs real	Spatial imagery	0.039	[0.017, 0.061]	3.470	0.001
Thinking language	Spatial imagery	-0.040	[-0.062, -0.018]	-3.601	<0.001
Math models	Spatial imagery	0.107	[0.085, 0.129]	9.630	<2.23e-04
Wide reach	Spatial imagery	0.063	[0.041, 0.085]	5.625	<2.23e-04
Rational self-interest	Spatial imagery	-0.010	[-0.031, 0.012]	-0.850	0.396
Neurobiology essential	Spatial imagery	0.052	[0.03, 0.074]	4.669	<2.23e-04
Holistic view	Spatial imagery	-0.055	[-0.077, -0.034]	-4.961	<2.23e-04
Personality stable	Spatial imagery	-0.010	[-0.032, 0.012]	-0.895	0.371
Context matters	Spatial imagery	-0.036	[-0.058, -0.014]	-3.250	0.001
Capacities innate	Spatial imagery	0.033	[0.011, 0.055]	2.940	0.003
Ideal rules	Spatial imagery	0.040	[0.018, 0.062]	3.552	<0.001
Evolution matters	Spatial imagery	0.032	[0.01, 0.054]	2.827	0.005
Computer analogy	Spatial imagery	-0.021	[-0.043, 0.001]	-1.895	0.058
Mind universal	Spatial imagery	0.055	[0.033, 0.077]	4.917	<2.23e-04
Social environment	Spatial imagery	-0.078	[-0.1, -0.056]	-6.998	<2.23e-04
Perception veridical	Spatial imagery	0.027	[0.005, 0.049]	2.444	0.015
Constructs real	Need for Cognition	0.027	[0.002, 0.053]	2.120	0.034
Thinking language	Need for Cognition	0.025	[0, 0.05]	1.932	0.053
Math models	Need for Cognition	0.047	[0.021, 0.072]	3.615	<0.001
Wide reach	Need for Cognition	0.038	[0.013, 0.063]	2.951	0.003
Rational self-interest	Need for Cognition	-0.100	[-0.125, -0.075]	-7.840	<2.23e-04
Neurobiology essential	Need for Cognition	0.023	[-0.003, 0.048]	1.734	0.083
Holistic view	Need for Cognition	0.034	[0.009, 0.059]	2.652	0.008

Supplementary Table 4: Regression coefficients, cognitive traits *(continued)*

Theme	Cognitive trait	Coefficient (β)	CIs	t	p
Personality stable	Need for Cognition	0.013	[-0.012, 0.038]	1.007	0.314
Context matters	Need for Cognition	0.023	[-0.003, 0.048]	1.767	0.077
Capacities innate	Need for Cognition	0.058	[0.033, 0.083]	4.515	<2.23e-04
Ideal rules	Need for Cognition	0.029	[0.004, 0.054]	2.272	0.023
Evolution matters	Need for Cognition	0.024	[-0.001, 0.049]	1.848	0.065
Computer analogy	Need for Cognition	-0.065	[-0.09, -0.04]	-5.114	<2.23e-04
Mind universal	Need for Cognition	0.047	[0.022, 0.072]	3.666	<0.001
Social environment	Need for Cognition	-0.027	[-0.052, -0.002]	-2.115	0.035
Perception veridical	Need for Cognition	-0.015	[-0.04, 0.01]	-1.178	0.239

Supplementary Table 5: Regression coefficients for controversial themes as a function of (binary) gender (each a separate regression model). Results correspond to Fig. 3d in the main text. Regression coefficients (b) represent logits, with the outcome (theme) standardized, for 'gender=male' compared to the reference level 'gender = female'. P-values in gray reflect cases that failed to meet the Bonferroni-adjusted significance threshold discussed in the main text ($p < .00313$). See `https://osf.io/zyec9/` for further results including nonbinary participants.

Theme	Gender (binary)	Coefficient (b)	CIs	t	p
Constructs real	male	0.145	[0.101, 0.189]	6.443	<3.13e-03
Thinking language	male	-0.024	[-0.069, 0.02]	-1.082	0.279
Math models	male	0.285	[0.241, 0.329]	12.733	<3.13e-03
Wide reach	male	0.074	[0.029, 0.118]	3.265	0.001
Rational self-interest	male	-0.174	[-0.218, -0.13]	-7.729	<3.13e-03
Neurobiology essential	male	0.024	[-0.02, 0.068]	1.065	0.287
Holistic view	male	-0.206	[-0.25, -0.162]	-9.181	<3.13e-03
Personality stable	male	0.020	[-0.025, 0.064]	0.872	0.383
Context matters	male	-0.077	[-0.121, -0.033]	-3.411	0.001
Capacities innate	male	0.103	[0.059, 0.148]	4.583	<3.13e-03
Ideal rules	male	0.159	[0.115, 0.203]	7.047	<3.13e-03
Evolution matters	male	0.250	[0.206, 0.294]	11.132	<3.13e-03
Computer analogy	male	-0.114	[-0.158, -0.07]	-5.044	<3.13e-03
Mind universal	male	0.219	[0.174, 0.263]	9.727	<3.13e-03
Social environment	male	-0.321	[-0.365, -0.277]	-14.381	<3.13e-03
Perception veridical	male	0.135	[0.091, 0.179]	5.986	<3.13e-03

Supplementary Table 6: Regression coefficients for research areas as a function of research methods. Results correspond to Extended Data Figure 4. Regression coefficients (b) represent logits. P-values in gray reflect cases that failed to meet the Bonferroni-adjusted significance threshold discussed in the caption of Extended Data Figure 4 ($p < .000152$).

Research area	Research method	Coefficient (b)	CIs	t	p
Biopsych	Behav expt: typical adults	0.029	[0.016, 0.041]	4.457	<1.52e-04
Clinical/health psych	Behav expt: typical adults	-0.135	[-0.155, -0.116]	-13.791	<1.52e-04
Cog neurosci	Behav expt: typical adults	0.177	[0.161, 0.193]	21.689	<1.52e-04
Cognitive psych	Behav expt: typical adults	0.336	[0.318, 0.355]	35.644	<1.52e-04
Comparative/animal behav	Behav expt: typical adults	0.003	[-0.006, 0.011]	0.618	0.536
Counseling	Behav expt: typical adults	-0.080	[-0.09, -0.069]	-14.400	<1.52e-04
Developmental psych	Behav expt: typical adults	-0.096	[-0.114, -0.079]	-10.637	<1.52e-04
Educational psych	Behav expt: typical adults	-0.073	[-0.087, -0.059]	-10.144	<1.52e-04
Evolutionary psych	Behav expt: typical adults	0.017	[0.006, 0.027]	3.129	0.002
Forensic psych	Behav expt: typical adults	-0.011	[-0.02, -0.003]	-2.563	0.010
Neuropsych	Behav expt: typical adults	0.030	[0.016, 0.043]	4.281	<1.52e-04
Occupational/IO psych	Behav expt: typical adults	-0.016	[-0.028, -0.003]	-2.435	0.015
Psychometrics	Behav expt: typical adults	-0.024	[-0.038, -0.009]	-3.258	0.001
Social psych	Behav expt: typical adults	0.074	[0.054, 0.093]	7.484	<1.52e-04
Personality psych	Behav expt: typical adults	-0.012	[-0.023, -0.001]	-2.152	0.031
Biopsych	Behav expt: children	0.011	[-0.006, 0.027]	1.287	0.198
Clinical/health psych	Behav expt: children	-0.043	[-0.068, -0.017]	-3.287	0.001
Cog neurosci	Behav expt: children	0.068	[0.046, 0.089]	6.159	<1.52e-04
Cognitive psych	Behav expt: children	0.178	[0.152, 0.203]	13.538	<1.52e-04
Comparative/animal behav	Behav expt: children	0.016	[0.006, 0.027]	3.021	0.003
Counseling	Behav expt: children	-0.044	[-0.059, -0.03]	-6.081	<1.52e-04
Developmental psych	Behav expt: children	0.490	[0.469, 0.511]	46.229	<1.52e-04
Educational psych	Behav expt: children	0.137	[0.118, 0.155]	14.605	<1.52e-04
Evolutionary psych	Behav expt: children	0.007	[-0.007, 0.02]	0.938	0.348
Forensic psych	Behav expt: children	-0.025	[-0.036, -0.013]	-4.245	<1.52e-04
Neuropsych	Behav expt: children	0.042	[0.024, 0.059]	4.614	<1.52e-04
Occupational/IO psych	Behav expt: children	-0.089	[-0.105, -0.073]	-10.689	<1.52e-04
Psychometrics	Behav expt: children	-0.026	[-0.045, -0.008]	-2.772	0.006
Social psych	Behav expt: children	-0.135	[-0.16, -0.11]	-10.528	<1.52e-04
Personality psych	Behav expt: children	-0.045	[-0.06, -0.031]	-6.214	<1.52e-04
Biopsych	Behav expt: atypical pop	0.084	[0.068, 0.1]	10.483	<1.52e-04
Clinical/health psych	Behav expt: atypical pop	0.150	[0.126, 0.174]	12.065	<1.52e-04
Cog neurosci	Behav expt: atypical pop	0.267	[0.247, 0.287]	26.161	<1.52e-04
Cognitive psych	Behav expt: atypical pop	0.177	[0.153, 0.202]	14.011	<1.52e-04
Comparative/animal behav	Behav expt: atypical pop	0.001	[-0.009, 0.011]	0.196	0.844
Counseling	Behav expt: atypical pop	-0.034	[-0.048, -0.02]	-4.855	<1.52e-04
Developmental psych	Behav expt: atypical pop	0.059	[0.037, 0.082]	5.156	<1.52e-04
Educational psych	Behav expt: atypical pop	-0.016	[-0.034, 0.002]	-1.756	0.079
Evolutionary psych	Behav expt: atypical pop	-0.014	[-0.027, -0.001]	-2.094	0.036
Forensic psych	Behav expt: atypical pop	0.003	[-0.008, 0.014]	0.557	0.577
Neuropsych	Behav expt: atypical pop	0.199	[0.183, 0.216]	23.638	<1.52e-04

Supplementary Table 6: Regression coefficients, research areas   methods *(continued)*

Research area	Research method	Coefficient (b)	CIs	t	p
Occupational/IO psych	Behav expt: atypical pop	-0.074	[-0.09, -0.059]	-9.234	<1.52e-04
Psychometrics	Behav expt: atypical pop	0.005	[-0.013, 0.023]	0.592	0.554
Social psych	Behav expt: atypical pop	-0.153	[-0.177, -0.129]	-12.381	<1.52e-04
Personality psych	Behav expt: atypical pop	-0.028	[-0.042, -0.014]	-3.964	<1.52e-04
Biopsych	Behav expt: animals	0.371	[0.345, 0.397]	28.146	<1.52e-04
Clinical/health psych	Behav expt: animals	-0.134	[-0.176, -0.092]	-6.281	<1.52e-04
Cog neurosci	Behav expt: animals	0.134	[0.099, 0.17]	7.419	<1.52e-04
Cognitive psych	Behav expt: animals	-0.045	[-0.088, -0.002]	-2.058	0.040
Comparative/animal behav	Behav expt: animals	0.437	[0.423, 0.452]	58.133	<1.52e-04
Counseling	Behav expt: animals	-0.062	[-0.085, -0.038]	-5.114	<1.52e-04
Developmental psych	Behav expt: animals	-0.015	[-0.054, 0.023]	-0.768	0.443
Educational psych	Behav expt: animals	-0.088	[-0.119, -0.058]	-5.676	<1.52e-04
Evolutionary psych	Behav expt: animals	0.182	[0.16, 0.204]	16.143	<1.52e-04
Forensic psych	Behav expt: animals	-0.022	[-0.041, -0.003]	-2.321	0.020
Neuropsych	Behav expt: animals	0.116	[0.087, 0.145]	7.810	<1.52e-04
Occupational/IO psych	Behav expt: animals	-0.075	[-0.102, -0.048]	-5.408	<1.52e-04
Psychometrics	Behav expt: animals	-0.053	[-0.084, -0.022]	-3.389	0.001
Social psych	Behav expt: animals	-0.179	[-0.221, -0.138]	-8.459	<1.52e-04
Personality psych	Behav expt: animals	-0.041	[-0.064, -0.017]	-3.377	0.001
Biopsych	Electrophys (EEG/ERP)	0.184	[0.165, 0.202]	19.925	<1.52e-04
Clinical/health psych	Electrophys (EEG/ERP)	-0.033	[-0.062, -0.005]	-2.274	0.023
Cog neurosci	Electrophys (EEG/ERP)	0.491	[0.47, 0.513]	44.229	<1.52e-04
Cognitive psych	Electrophys (EEG/ERP)	0.238	[0.21, 0.267]	16.208	<1.52e-04
Comparative/animal behav	Electrophys (EEG/ERP)	0.002	[-0.01, 0.014]	0.351	0.726
Counseling	Electrophys (EEG/ERP)	-0.044	[-0.061, -0.028]	-5.406	<1.52e-04
Developmental psych	Electrophys (EEG/ERP)	-0.015	[-0.041, 0.012]	-1.087	0.277
Educational psych	Electrophys (EEG/ERP)	-0.061	[-0.081, -0.04]	-5.689	<1.52e-04
Evolutionary psych	Electrophys (EEG/ERP)	-0.002	[-0.018, 0.013]	-0.300	0.764
Forensic psych	Electrophys (EEG/ERP)	-0.019	[-0.031, -0.006]	-2.854	0.004
Neuropsych	Electrophys (EEG/ERP)	0.186	[0.167, 0.206]	18.745	<1.52e-04
Occupational/IO psych	Electrophys (EEG/ERP)	-0.076	[-0.094, -0.057]	-8.045	<1.52e-04
Psychometrics	Electrophys (EEG/ERP)	-0.003	[-0.024, 0.018]	-0.280	0.780
Social psych	Electrophys (EEG/ERP)	-0.141	[-0.169, -0.112]	-9.731	<1.52e-04
Personality psych	Electrophys (EEG/ERP)	-0.025	[-0.041, -0.009]	-3.036	0.002
Biopsych	Electrophys (single cell)	0.412	[0.36, 0.464]	15.548	<1.52e-04
Clinical/health psych	Electrophys (single cell)	-0.159	[-0.24, -0.077]	-3.810	<1.52e-04
Cog neurosci	Electrophys (single cell)	0.463	[0.394, 0.531]	13.246	<1.52e-04
Cognitive psych	Electrophys (single cell)	0.016	[-0.067, 0.099]	0.375	0.707
Comparative/animal behav	Electrophys (single cell)	0.211	[0.177, 0.245]	12.169	<1.52e-04
Counseling	Electrophys (single cell)	-0.059	[-0.105, -0.013]	-2.516	0.012
Developmental psych	Electrophys (single cell)	-0.107	[-0.182, -0.032]	-2.799	0.005
Educational psych	Electrophys (single cell)	-0.076	[-0.136, -0.017]	-2.509	0.012
Evolutionary psych	Electrophys (single cell)	-0.016	[-0.06, 0.027]	-0.735	0.463
Forensic psych	Electrophys (single cell)	-0.041	[-0.078, -0.005]	-2.224	0.026

Supplementary Table 6: Regression coefficients, research areas methods *(continued)*

Research area	Research method	Coefficient (b)	CIs	t	p
Neuropsych	Electrophys (single cell)	0.227	[0.171, 0.284]	7.889	<1.52e-04
Occupational/IO psych	Electrophys (single cell)	-0.065	[-0.118, -0.012]	-2.418	0.016
Psychometrics	Electrophys (single cell)	-0.043	[-0.103, 0.017]	-1.402	0.161
Social psych	Electrophys (single cell)	-0.188	[-0.269, -0.107]	-4.545	<1.52e-04
Personality psych	Electrophys (single cell)	-0.050	[-0.096, -0.004]	-2.143	0.032
Biopsych	Neuroimaging	0.172	[0.154, 0.19]	18.605	<1.52e-04
Clinical/health psych	Neuroimaging	0.025	[-0.004, 0.054]	1.709	0.088
Cog neurosci	Neuroimaging	0.608	[0.588, 0.628]	58.624	<1.52e-04
Cognitive psych	Neuroimaging	0.146	[0.117, 0.175]	9.795	<1.52e-04
Comparative/animal behav	Neuroimaging	0.016	[0.004, 0.028]	2.672	0.008
Counseling	Neuroimaging	-0.055	[-0.071, -0.039]	-6.723	<1.52e-04
Developmental psych	Neuroimaging	-0.029	[-0.056, -0.003]	-2.190	0.029
Educational psych	Neuroimaging	-0.078	[-0.099, -0.057]	-7.316	<1.52e-04
Evolutionary psych	Neuroimaging	-0.004	[-0.02, 0.011]	-0.562	0.574
Forensic psych	Neuroimaging	-0.024	[-0.037, -0.011]	-3.680	<0.001
Neuropsych	Neuroimaging	0.258	[0.239, 0.277]	26.433	<1.52e-04
Occupational/IO psych	Neuroimaging	-0.083	[-0.102, -0.065]	-8.848	<1.52e-04
Psychometrics	Neuroimaging	-0.034	[-0.055, -0.013]	-3.198	0.001
Social psych	Neuroimaging	-0.173	[-0.201, -0.145]	-11.997	<1.52e-04
Personality psych	Neuroimaging	-0.033	[-0.049, -0.016]	-3.947	<1.52e-04
Biopsych	Observational Studies	-0.010	[-0.023, 0.003]	-1.488	0.137
Clinical/health psych	Observational Studies	0.079	[0.058, 0.099]	7.549	<1.52e-04
Cog neurosci	Observational Studies	-0.112	[-0.129, -0.095]	-12.709	<1.52e-04
Cognitive psych	Observational Studies	-0.105	[-0.126, -0.084]	-9.865	<1.52e-04
Comparative/animal behav	Observational Studies	0.008	[-0.001, 0.017]	1.791	0.073
Counseling	Observational Studies	0.007	[-0.004, 0.019]	1.266	0.205
Developmental psych	Observational Studies	0.128	[0.109, 0.146]	13.397	<1.52e-04
Educational psych	Observational Studies	0.080	[0.065, 0.095]	10.520	<1.52e-04
Evolutionary psych	Observational Studies	0.016	[0.005, 0.027]	2.802	0.005
Forensic psych	Observational Studies	0.005	[-0.005, 0.014]	0.982	0.326
Neuropsych	Observational Studies	-0.009	[-0.023, 0.005]	-1.232	0.218
Occupational/IO psych	Observational Studies	0.025	[0.012, 0.038]	3.665	<0.001
Psychometrics	Observational Studies	0.053	[0.038, 0.068]	6.947	<1.52e-04
Social psych	Observational Studies	0.047	[0.026, 0.067]	4.481	<1.52e-04
Personality psych	Observational Studies	0.004	[-0.007, 0.016]	0.760	0.447
Biopsych	Comp ling/corpus analysis	-0.052	[-0.078, -0.026]	-3.970	<1.52e-04
Clinical/health psych	Comp ling/corpus analysis	-0.191	[-0.231, -0.152]	-9.461	<1.52e-04
Cog neurosci	Comp ling/corpus analysis	0.032	[-0.001, 0.066]	1.874	0.061
Cognitive psych	Comp ling/corpus analysis	0.323	[0.283, 0.363]	15.821	<1.52e-04
Comparative/animal behav	Comp ling/corpus analysis	-0.011	[-0.028, 0.006]	-1.295	0.195
Counseling	Comp ling/corpus analysis	-0.046	[-0.069, -0.024]	-4.044	<1.52e-04
Developmental psych	Comp ling/corpus analysis	0.007	[-0.03, 0.043]	0.359	0.719
Educational psych	Comp ling/corpus analysis	-0.028	[-0.057, 0.001]	-1.889	0.059
Evolutionary psych	Comp ling/corpus analysis	-0.001	[-0.022, 0.021]	-0.063	0.950

Supplementary Table 6: Regression coefficients, research areas methods *(continued)*

Research area	Research method	Coefficient (b)	CIs	t	p
Forensic psych	Comp ling/corpus analysis	-0.018	[-0.036, 0]	-2.015	0.044
Neuropsych	Comp ling/corpus analysis	-0.011	[-0.038, 0.017]	-0.754	0.451
Occupational/IO psych	Comp ling/corpus analysis	-0.023	[-0.049, 0.003]	-1.744	0.081
Psychometrics	Comp ling/corpus analysis	0.003	[-0.026, 0.033]	0.232	0.817
Social psych	Comp ling/corpus analysis	-0.034	[-0.073, 0.006]	-1.671	0.095
Personality psych	Comp ling/corpus analysis	-0.036	[-0.058, -0.013]	-3.134	0.002
Biopsych	Comp/math modeling	0.020	[0.004, 0.037]	2.421	0.016
Clinical/health psych	Comp/math modeling	-0.134	[-0.159, -0.108]	-10.320	<1.52e-04
Cog neurosci	Comp/math modeling	0.143	[0.122, 0.165]	13.094	<1.52e-04
Cognitive psych	Comp/math modeling	0.241	[0.216, 0.267]	18.491	<1.52e-04
Comparative/animal behav	Comp/math modeling	0.019	[0.009, 0.03]	3.568	<0.001
Counseling	Comp/math modeling	-0.050	[-0.064, -0.036]	-6.842	<1.52e-04
Developmental psych	Comp/math modeling	-0.072	[-0.095, -0.048]	-6.004	<1.52e-04
Educational psych	Comp/math modeling	-0.020	[-0.038, -0.001]	-2.059	0.040
Evolutionary psych	Comp/math modeling	0.012	[-0.001, 0.026]	1.767	0.077
Forensic psych	Comp/math modeling	-0.012	[-0.024, -0.001]	-2.104	0.035
Neuropsych	Comp/math modeling	0.007	[-0.011, 0.024]	0.728	0.467
Occupational/IO psych	Comp/math modeling	0.003	[-0.014, 0.019]	0.329	0.742
Psychometrics	Comp/math modeling	0.117	[0.098, 0.136]	12.347	<1.52e-04
Social psych	Comp/math modeling	-0.060	[-0.085, -0.035]	-4.631	<1.52e-04
Personality psych	Comp/math modeling	-0.016	[-0.03, -0.001]	-2.136	0.033
Biopsych	Clin intervention	0.031	[0.015, 0.047]	3.872	<1.52e-04
Clinical/health psych	Clin intervention	0.557	[0.536, 0.578]	51.027	<1.52e-04
Cog neurosci	Clin intervention	-0.036	[-0.057, -0.015]	-3.392	0.001
Cognitive psych	Clin intervention	-0.193	[-0.218, -0.169]	-15.280	<1.52e-04
Comparative/animal behav	Clin intervention	-0.027	[-0.037, -0.017]	-5.100	<1.52e-04
Counseling	Clin intervention	0.132	[0.119, 0.146]	19.107	<1.52e-04
Developmental psych	Clin intervention	0.018	[-0.005, 0.04]	1.518	0.129
Educational psych	Clin intervention	-0.039	[-0.057, -0.022]	-4.310	<1.52e-04
Evolutionary psych	Clin intervention	-0.025	[-0.038, -0.012]	-3.719	<0.001
Forensic psych	Clin intervention	0.042	[0.031, 0.053]	7.519	<1.52e-04
Neuropsych	Clin intervention	0.080	[0.063, 0.097]	9.231	<1.52e-04
Occupational/IO psych	Clin intervention	-0.056	[-0.072, -0.041]	-6.977	<1.52e-04
Psychometrics	Clin intervention	0.030	[0.012, 0.048]	3.290	0.001
Social psych	Clin intervention	-0.129	[-0.154, -0.105]	-10.421	<1.52e-04
Personality psych	Clin intervention	0.009	[-0.005, 0.023]	1.306	0.192
Biopsych	Case study	-0.051	[-0.067, -0.035]	-6.390	<1.52e-04
Clinical/health psych	Case study	0.104	[0.08, 0.128]	8.397	<1.52e-04
Cog neurosci	Case study	-0.075	[-0.096, -0.055]	-7.164	<1.52e-04
Cognitive psych	Case study	-0.106	[-0.131, -0.081]	-8.395	<1.52e-04
Comparative/animal behav	Case study	-0.020	[-0.031, -0.01]	-3.932	<1.52e-04
Counseling	Case study	0.127	[0.113, 0.14]	18.519	<1.52e-04
Developmental psych	Case study	0.014	[-0.008, 0.037]	1.249	0.212
Educational psych	Case study	0.095	[0.078, 0.113]	10.600	<1.52e-04

Supplementary Table 6: Regression coefficients, research areas methods *(continued)*

Research area	Research method	Coefficient (b)	CIs	t	p
Evolutionary psych	Case study	-0.012	[-0.025, 0.001]	-1.751	0.080
Forensic psych	Case study	0.047	[0.036, 0.057]	8.459	<1.52e-04
Neuropsych	Case study	0.036	[0.019, 0.053]	4.186	<1.52e-04
Occupational/IO psych	Case study	0.062	[0.047, 0.078]	7.809	<1.52e-04
Psychometrics	Case study	-0.015	[-0.033, 0.003]	-1.615	0.106
Social psych	Case study	0.018	[-0.006, 0.042]	1.460	0.144
Personality psych	Case study	0.003	[-0.011, 0.016]	0.386	0.700
Biopsych	Surveys	-0.075	[-0.087, -0.062]	-11.611	<1.52e-04
Clinical/health psych	Surveys	0.084	[0.064, 0.104]	8.385	<1.52e-04
Cog neurosci	Surveys	-0.214	[-0.23, -0.198]	-26.164	<1.52e-04
Cognitive psych	Surveys	-0.192	[-0.212, -0.173]	-19.150	<1.52e-04
Comparative/animal behav	Surveys	-0.043	[-0.051, -0.035]	-10.239	<1.52e-04
Counseling	Surveys	0.020	[0.009, 0.031]	3.579	<0.001
Developmental psych	Surveys	-0.005	[-0.023, 0.013]	-0.517	0.605
Educational psych	Surveys	0.050	[0.035, 0.064]	6.779	<1.52e-04
Evolutionary psych	Surveys	-0.018	[-0.029, -0.008]	-3.373	0.001
Forensic psych	Surveys	0.006	[-0.003, 0.015]	1.335	0.182
Neuropsych	Surveys	-0.094	[-0.108, -0.081]	-13.623	<1.52e-04
Occupational/IO psych	Surveys	0.102	[0.089, 0.114]	15.942	<1.52e-04
Psychometrics	Surveys	0.069	[0.055, 0.084]	9.462	<1.52e-04
Social psych	Surveys	0.216	[0.197, 0.235]	22.267	<1.52e-04
Personality psych	Surveys	0.058	[0.047, 0.069]	10.294	<1.52e-04
Biopsych	Meta-analyses	-0.005	[-0.021, 0.012]	-0.563	0.574
Clinical/health psych	Meta-analyses	0.078	[0.053, 0.103]	6.069	<1.52e-04
Cog neurosci	Meta-analyses	-0.020	[-0.041, 0.001]	-1.848	0.065
Cognitive psych	Meta-analyses	-0.059	[-0.084, -0.033]	-4.489	<1.52e-04
Comparative/animal behav	Meta-analyses	-0.017	[-0.028, -0.007]	-3.188	0.001
Counseling	Meta-analyses	0.005	[-0.009, 0.019]	0.718	0.473
Developmental psych	Meta-analyses	-0.021	[-0.044, 0.002]	-1.776	0.076
Educational psych	Meta-analyses	0.025	[0.006, 0.043]	2.650	0.008
Evolutionary psych	Meta-analyses	-0.003	[-0.017, 0.01]	-0.508	0.611
Forensic psych	Meta-analyses	0.016	[0.005, 0.028]	2.866	0.004
Neuropsych	Meta-analyses	0.002	[-0.015, 0.02]	0.242	0.809
Occupational/IO psych	Meta-analyses	0.054	[0.038, 0.071]	6.590	<1.52e-04
Psychometrics	Meta-analyses	0.073	[0.055, 0.092]	7.819	<1.52e-04
Social psych	Meta-analyses	0.038	[0.013, 0.063]	3.001	0.003
Personality psych	Meta-analyses	0.028	[0.013, 0.042]	3.824	<1.52e-04
Biopsych	Pharm intervention	0.356	[0.328, 0.385]	24.649	<1.52e-04
Clinical/health psych	Pharm intervention	0.220	[0.175, 0.266]	9.518	<1.52e-04
Cog neurosci	Pharm intervention	0.206	[0.168, 0.245]	10.534	<1.52e-04
Cognitive psych	Pharm intervention	-0.135	[-0.182, -0.089]	-5.710	<1.52e-04
Comparative/animal behav	Pharm intervention	0.130	[0.111, 0.149]	13.486	<1.52e-04
Counseling	Pharm intervention	-0.001	[-0.027, 0.024]	-0.113	0.910
Developmental psych	Pharm intervention	-0.102	[-0.144, -0.06]	-4.791	<1.52e-04

Supplementary Table 6: Regression coefficients, research areas methods *(continued)*

Research area	Research method	Coefficient (b)	CIs	t	p
Educational psych	Pharm intervention	-0.091	[-0.125, -0.058]	-5.408	<1.52e-04
Evolutionary psych	Pharm intervention	0.006	[-0.018, 0.03]	0.497	0.619
Forensic psych	Pharm intervention	0.007	[-0.013, 0.027]	0.658	0.511
Neuropsych	Pharm intervention	0.218	[0.187, 0.25]	13.671	<1.52e-04
Occupational/IO psych	Pharm intervention	-0.064	[-0.094, -0.035]	-4.297	<1.52e-04
Psychometrics	Pharm intervention	-0.003	[-0.036, 0.031]	-0.168	0.867
Social psych	Pharm intervention	-0.194	[-0.239, -0.149]	-8.443	<1.52e-04
Personality psych	Pharm intervention	-0.024	[-0.049, 0.002]	-1.801	0.072
Biopsych	Eye-tracking	0.029	[0.011, 0.047]	3.132	0.002
Clinical/health psych	Eye-tracking	-0.157	[-0.185, -0.129]	-10.934	<1.52e-04
Cog neurosci	Eye-tracking	0.265	[0.242, 0.289]	22.367	<1.52e-04
Cognitive psych	Eye-tracking	0.378	[0.35, 0.406]	26.757	<1.52e-04
Comparative/animal behav	Eye-tracking	-0.001	[-0.013, 0.011]	-0.224	0.823
Counseling	Eye-tracking	-0.064	[-0.08, -0.048]	-7.872	<1.52e-04
Developmental psych	Eye-tracking	0.028	[0.002, 0.054]	2.096	0.036
Educational psych	Eye-tracking	-0.036	[-0.056, -0.015]	-3.403	0.001
Evolutionary psych	Eye-tracking	-0.016	[-0.031, -0.001]	-2.095	0.036
Forensic psych	Eye-tracking	-0.022	[-0.034, -0.009]	-3.380	0.001
Neuropsych	Eye-tracking	0.048	[0.029, 0.068]	4.829	<1.52e-04
Occupational/IO psych	Eye-tracking	-0.057	[-0.075, -0.039]	-6.119	<1.52e-04
Psychometrics	Eye-tracking	-0.020	[-0.041, 0.001]	-1.897	0.058
Social psych	Eye-tracking	-0.109	[-0.137, -0.081]	-7.666	<1.52e-04
Personality psych	Eye-tracking	-0.048	[-0.064, -0.032]	-5.913	<1.52e-04
Biopsych	Cranial stim (e.g. TMS; tDCS)	0.217	[0.184, 0.251]	12.684	<1.52e-04
Clinical/health psych	Cranial stim (e.g. TMS; tDCS)	-0.046	[-0.098, 0.007]	-1.704	0.088
Cog neurosci	Cranial stim (e.g. TMS; tDCS)	0.652	[0.61, 0.694]	30.336	<1.52e-04
Cognitive psych	Cranial stim (e.g. TMS; tDCS)	0.245	[0.191, 0.298]	8.984	<1.52e-04
Comparative/animal behav	Cranial stim (e.g. TMS; tDCS)	0.014	[-0.008, 0.036]	1.239	0.215
Counseling	Cranial stim (e.g. TMS; tDCS)	-0.051	[-0.081, -0.021]	-3.386	0.001
Developmental psych	Cranial stim (e.g. TMS; tDCS)	-0.101	[-0.149, -0.053]	-4.098	<1.52e-04
Educational psych	Cranial stim (e.g. TMS; tDCS)	-0.079	[-0.117, -0.041]	-4.053	<1.52e-04
Evolutionary psych	Cranial stim (e.g. TMS; tDCS)	-0.011	[-0.039, 0.017]	-0.745	0.456
Forensic psych	Cranial stim (e.g. TMS; tDCS)	-0.020	[-0.044, 0.003]	-1.696	0.090
Neuropsych	Cranial stim (e.g. TMS; tDCS)	0.306	[0.27, 0.342]	16.723	<1.52e-04
Occupational/IO psych	Cranial stim (e.g. TMS; tDCS)	-0.075	[-0.109, -0.041]	-4.353	<1.52e-04
Psychometrics	Cranial stim (e.g. TMS; tDCS)	-0.041	[-0.08, -0.002]	-2.085	0.037
Social psych	Cranial stim (e.g. TMS; tDCS)	-0.178	[-0.23, -0.126]	-6.713	<1.52e-04
Personality psych	Cranial stim (e.g. TMS; tDCS)	-0.040	[-0.07, -0.011]	-2.665	0.008
Biopsych	Interviews	-0.070	[-0.083, -0.058]	-10.803	<1.52e-04
Clinical/health psych	Interviews	0.164	[0.144, 0.183]	16.350	<1.52e-04
Cog neurosci	Interviews	-0.171	[-0.187, -0.154]	-20.349	<1.52e-04
Cognitive psych	Interviews	-0.207	[-0.227, -0.188]	-20.488	<1.52e-04
Comparative/animal behav	Interviews	-0.038	[-0.046, -0.029]	-8.896	<1.52e-04
Counseling	Interviews	0.073	[0.062, 0.084]	12.853	<1.52e-04

Supplementary Table 6: Regression coefficients, research areas methods *(continued)*

Research area	Research method	Coefficient (b)	CIs	t	p
Developmental psych	Interviews	0.060	[0.042, 0.078]	6.423	<1.52e-04
Educational psych	Interviews	0.080	[0.065, 0.094]	10.862	<1.52e-04
Evolutionary psych	Interviews	-0.028	[-0.039, -0.017]	-5.180	<1.52e-04
Forensic psych	Interviews	0.035	[0.026, 0.044]	7.822	<1.52e-04
Neuropsych	Interviews	-0.053	[-0.066, -0.039]	-7.477	<1.52e-04
Occupational/IO psych	Interviews	0.074	[0.061, 0.087]	11.393	<1.52e-04
Psychometrics	Interviews	0.010	[-0.004, 0.025]	1.394	0.163
Social psych	Interviews	0.117	[0.098, 0.137]	11.718	<1.52e-04
Personality psych	Interviews	0.016	[0.005, 0.027]	2.778	0.005
Biopsych	Longitudinal studies	0.000	[-0.014, 0.013]	-0.060	0.952
Clinical/health psych	Longitudinal studies	0.103	[0.082, 0.124]	9.516	<1.52e-04
Cog neurosci	Longitudinal studies	-0.059	[-0.077, -0.041]	-6.412	<1.52e-04
Cognitive psych	Longitudinal studies	-0.146	[-0.168, -0.125]	-13.284	<1.52e-04
Comparative/animal behav	Longitudinal studies	-0.022	[-0.031, -0.013]	-4.771	<1.52e-04
Counseling	Longitudinal studies	-0.020	[-0.032, -0.008]	-3.312	0.001
Developmental psych	Longitudinal studies	0.192	[0.173, 0.212]	19.681	<1.52e-04
Educational psych	Longitudinal studies	0.041	[0.026, 0.057]	5.203	<1.52e-04
Evolutionary psych	Longitudinal studies	-0.022	[-0.033, -0.01]	-3.699	<0.001
Forensic psych	Longitudinal studies	-0.014	[-0.024, -0.005]	-2.891	0.004
Neuropsych	Longitudinal studies	0.013	[-0.001, 0.028]	1.763	0.078
Occupational/IO psych	Longitudinal studies	0.041	[0.027, 0.055]	5.823	<1.52e-04
Psychometrics	Longitudinal studies	0.072	[0.057, 0.088]	9.076	<1.52e-04
Social psych	Longitudinal studies	0.033	[0.012, 0.054]	3.065	0.002
Personality psych	Longitudinal studies	0.039	[0.027, 0.051]	6.317	<1.52e-04
Biopsych	Behavioral intervention	0.037	[0.022, 0.052]	4.878	<1.52e-04
Clinical/health psych	Behavioral intervention	0.269	[0.247, 0.292]	23.528	<1.52e-04
Cog neurosci	Behavioral intervention	-0.061	[-0.081, -0.042]	-6.114	<1.52e-04
Cognitive psych	Behavioral intervention	-0.117	[-0.141, -0.094]	-9.744	<1.52e-04
Comparative/animal behav	Behavioral intervention	-0.010	[-0.02, 0]	-2.055	0.040
Counseling	Behavioral intervention	0.025	[0.012, 0.038]	3.770	<0.001
Developmental psych	Behavioral intervention	0.035	[0.014, 0.056]	3.205	0.001
Educational psych	Behavioral intervention	0.044	[0.027, 0.061]	5.139	<1.52e-04
Evolutionary psych	Behavioral intervention	-0.027	[-0.039, -0.014]	-4.229	<1.52e-04
Forensic psych	Behavioral intervention	0.004	[-0.006, 0.015]	0.811	0.418
Neuropsych	Behavioral intervention	0.029	[0.013, 0.045]	3.513	<0.001
Occupational/IO psych	Behavioral intervention	0.015	[0, 0.03]	1.936	0.053
Psychometrics	Behavioral intervention	0.026	[0.009, 0.043]	2.993	0.003
Social psych	Behavioral intervention	-0.013	[-0.036, 0.01]	-1.068	0.286
Personality psych	Behavioral intervention	-0.006	[-0.019, 0.007]	-0.850	0.395
Biopsych	Twin studies	0.126	[0.079, 0.173]	5.270	<1.52e-04
Clinical/health psych	Twin studies	0.056	[-0.017, 0.129]	1.499	0.134
Cog neurosci	Twin studies	-0.012	[-0.074, 0.05]	-0.374	0.708
Cognitive psych	Twin studies	-0.069	[-0.144, 0.005]	-1.822	0.069
Comparative/animal behav	Twin studies	0.020	[-0.011, 0.05]	1.261	0.207

Supplementary Table 6: Regression coefficients, research areas   methods *(continued)*

Research area	Research method	Coefficient (b)	CIs	t	p
Counseling	Twin studies	-0.033	[-0.074, 0.008]	-1.575	0.115
Developmental psych	Twin studies	0.222	[0.155, 0.289]	6.515	<1.52e-04
Educational psych	Twin studies	0.020	[-0.033, 0.073]	0.730	0.466
Evolutionary psych	Twin studies	0.157	[0.118, 0.196]	7.948	<1.52e-04
Forensic psych	Twin studies	0.001	[-0.032, 0.033]	0.046	0.964
Neuropsych	Twin studies	0.039	[-0.011, 0.09]	1.522	0.128
Occupational/IO psych	Twin studies	-0.043	[-0.09, 0.004]	-1.778	0.075
Psychometrics	Twin studies	0.151	[0.098, 0.205]	5.550	<1.52e-04
Social psych	Twin studies	-0.065	[-0.137, 0.008]	-1.749	0.080
Personality psych	Twin studies	0.135	[0.095, 0.176]	6.483	<1.52e-04
Biopsych	Self-report/guided writing	-0.022	[-0.038, -0.005]	-2.547	0.011
Clinical/health psych	Self-report/guided writing	0.105	[0.08, 0.131]	8.041	<1.52e-04
Cog neurosci	Self-report/guided writing	-0.094	[-0.116, -0.072]	-8.468	<1.52e-04
Cognitive psych	Self-report/guided writing	-0.124	[-0.15, -0.097]	-9.245	<1.52e-04
Comparative/animal behav	Self-report/guided writing	-0.028	[-0.039, -0.018]	-5.156	<1.52e-04
Counseling	Self-report/guided writing	0.056	[0.041, 0.07]	7.523	<1.52e-04
Developmental psych	Self-report/guided writing	0.043	[0.019, 0.066]	3.548	<0.001
Educational psych	Self-report/guided writing	0.071	[0.053, 0.09]	7.470	<1.52e-04
Evolutionary psych	Self-report/guided writing	-0.003	[-0.016, 0.011]	-0.358	0.720
Forensic psych	Self-report/guided writing	0.005	[-0.007, 0.016]	0.776	0.438
Neuropsych	Self-report/guided writing	-0.033	[-0.05, -0.015]	-3.560	<0.001
Occupational/IO psych	Self-report/guided writing	0.041	[0.024, 0.057]	4.778	<1.52e-04
Psychometrics	Self-report/guided writing	0.054	[0.035, 0.073]	5.602	<1.52e-04
Social psych	Self-report/guided writing	0.159	[0.134, 0.185]	12.306	<1.52e-04
Personality psych	Self-report/guided writing	0.097	[0.082, 0.111]	13.186	<1.52e-04

Supplementary Table 7: Regression coefficients for each latent theme factor as a function of select cognitive traits, corresponding to Fig. 6b in the main text. Each latent factor outcome represents one model with multiple predictors. Regression coefficients (beta) reflect outcomes and predictors all having been standardized.

Latent factor	Cognitive trait	Coefficient (β)	CIs	t	p
1: Essential	Tolerance of Ambiguity	-0.133	[-0.183, -0.084]	-5.245	< 0.001
1: Essential	Need for Cognition	0.139	[0.094, 0.184]	6.068	< 0.001
1: Essential	Verbal orientation	-0.007	[-0.045, 0.032]	-0.344	0.731
1: Essential	Spatial imagery	0.012	[-0.007, 0.03]	1.226	0.220
1: Essential	Cognitive structure	0.001	[-0.032, 0.034]	0.084	0.933
1: Essential	Breadth of interest	-0.036	[-0.066, -0.006]	-2.378	0.017
2: Biological	Tolerance of Ambiguity	-0.232	[-0.283, -0.181]	-8.940	< 0.001
2: Biological	Need for Cognition	0.104	[0.058, 0.15]	4.455	< 0.001
2: Biological	Verbal orientation	-0.097	[-0.136, -0.058]	-4.872	< 0.001
2: Biological	Spatial imagery	0.041	[0.022, 0.06]	4.205	< 0.001
2: Biological	Cognitive structure	-0.004	[-0.037, 0.03]	-0.228	0.820
2: Biological	Breadth of interest	0.045	[0.014, 0.075]	2.898	0.004
3: Logical	Tolerance of Ambiguity	-0.276	[-0.323, -0.229]	-11.551	< 0.001
3: Logical	Need for Cognition	-0.069	[-0.111, -0.027]	-3.197	0.001
3: Logical	Verbal orientation	-0.010	[-0.046, 0.026]	-0.544	0.587
3: Logical	Spatial imagery	0.028	[0.01, 0.045]	3.104	0.002
3: Logical	Cognitive structure	0.120	[0.089, 0.151]	7.570	< 0.001
3: Logical	Breadth of interest	-0.041	[-0.068, -0.013]	-2.863	0.004
4: Contextual	Tolerance of Ambiguity	0.346	[0.289, 0.404]	11.798	< 0.001
4: Contextual	Need for Cognition	-0.130	[-0.182, -0.078]	-4.915	< 0.001
4: Contextual	Verbal orientation	0.136	[0.091, 0.18]	6.007	< 0.001
4: Contextual	Spatial imagery	-0.086	[-0.108, -0.065]	-7.856	< 0.001
4: Contextual	Cognitive structure	-0.026	[-0.064, 0.012]	-1.355	0.176
4: Contextual	Breadth of interest	0.080	[0.046, 0.114]	4.594	< 0.001
5: Objective	Tolerance of Ambiguity	-0.401	[-0.454, -0.348]	-14.784	< 0.001
5: Objective	Need for Cognition	0.191	[0.143, 0.239]	7.844	< 0.001
5: Objective	Verbal orientation	-0.138	[-0.179, -0.097]	-6.611	< 0.001
5: Objective	Spatial imagery	0.076	[0.056, 0.096]	7.506	< 0.001
5: Objective	Cognitive structure	0.019	[-0.016, 0.054]	1.055	0.291
5: Objective	Breadth of interest	-0.034	[-0.065, -0.002]	-2.093	0.036

Appendix 3 Additional details on the bibliometric models

To create the input for the semantics model, we first concatenated the title and the abstract of each article. Then, we used spaCy (https://spacy.io/) library to tokenize the words in each article. Words that were classified as punctuation or number by the tokenizer were thrown out. We also removed the stop words included in the spaCy model. Finally, a bigram detector implemented in Gensim[1] package (the phrases module) was trained on the whole corpus and was applied to the corpus. We used the distributed memory training algorithm for the Doc2vec[2] algorithm. All hyperparameters were identical to the default value in the Gensim package, except that we used 128 dimensions for the vector space (this was to match the citation and the co-authorship models). For the citation and co-authorship networks, we removed the self-loops in the network. We used the Node2vec[3] algorithm implemented in https://github.com/eliorc/node2vec with all hyperparameters set as default in the implementation.

Appendix 4 Structural equation models

In addition to the linear regressions reported in the main text, we report two Structural Equation Models (SEMs) that encompass multiple relationships between the variables. In the model summaries below, 'topic' refers to surveyed research topics; 'trait' refers to cognitive traits; 'branch' to research area; 'controversy' to controversial themes; 'method' to research methods; 'coauthor' to the bibliometric model of co-authorship; 'semantics' to the bibliometric model of abstract texts; and 'citations' to the bibliometric model of citation practices.

The first SEM, which includes semantic similarity but not citation similarity, is specified as:

$$
\begin{gathered}
topic \sim trait \\
branch \sim trait + topic \\
method \sim trait + branch \\
coauthor \sim trait + topic + branch + method \\
controversy \sim trait + topic + branch + method \\
semantics \sim trait + topic + branch + method + coauthor + controversy
\end{gathered}
$$

Supplementary Table 8 and Supplementary Figure 1 below show the results from the first SEM.

The second SEM, which includes citation similarity but not semantic similarity, is specified as:

$$
\begin{gathered}
topic \sim trait \\
branch \sim trait + topic \\
method \sim trait + branch \\
coauthor \sim trait + topic + branch + method \\
controversy \sim trait + topic + branch + method \\
citations \sim trait + topic + branch + method + coauthor + controversy
\end{gathered}
$$

Supplementary Table 9 and Supplementary Figure 2 below show the results from the second SEM.

Supplementary Table 8: Structural Equation Model 1 Results

DV	IV	Estimate	Std. Err.	z-value	p-value	95% CI
topic	trait	0.008	< 0.001	31.149	< 0.001	[0.007, 0.008]
branch	trait	0.009	< 0.001	35.789	< 0.001	[0.008, 0.009]
branch	topic	0.153	< 0.001	761.663	< 0.001	[0.153, 0.154]
method	trait	0.029	< 0.001	128.438	< 0.001	[0.028, 0.029]
method	branch	0.236	< 0.001	1245.784	< 0.001	[0.235, 0.236]
coauthor	trait	-0.002	< 0.001	-8.692	< 0.001	[-0.002, -0.001]
coauthor	topic	0.038	< 0.001	203.614	< 0.001	[0.038, 0.038]
coauthor	branch	0.115	< 0.001	589.162	< 0.001	[0.114, 0.115]
coauthor	method	0.069	< 0.001	338.707	< 0.001	[0.069, 0.069]
controversy	trait	0.089	< 0.001	432.632	< 0.001	[0.088, 0.089]
controversy	topic	0.003	< 0.001	16.647	< 0.001	[0.003, 0.003]
controversy	branch	0.025	< 0.001	136.407	< 0.001	[0.025, 0.025]
controversy	method	0.068	< 0.001	353.805	< 0.001	[0.067, 0.068]
semantics	trait	0.006	< 0.001	32.560	< 0.001	[0.006, 0.007]
semantics	topic	0.053	< 0.001	328.737	< 0.001	[0.052, 0.053]
semantics	branch	0.106	< 0.001	633.113	< 0.001	[0.106, 0.107]
semantics	method	0.092	< 0.001	521.997	< 0.001	[0.091, 0.092]
semantics	coauthor	0.025	< 0.001	142.990	< 0.001	[0.025, 0.026]
semantics	controversy	0.027	< 0.001	141.096	< 0.001	[0.026, 0.027]

Supplementary Figure 1: Structural Equation Model 1 Results

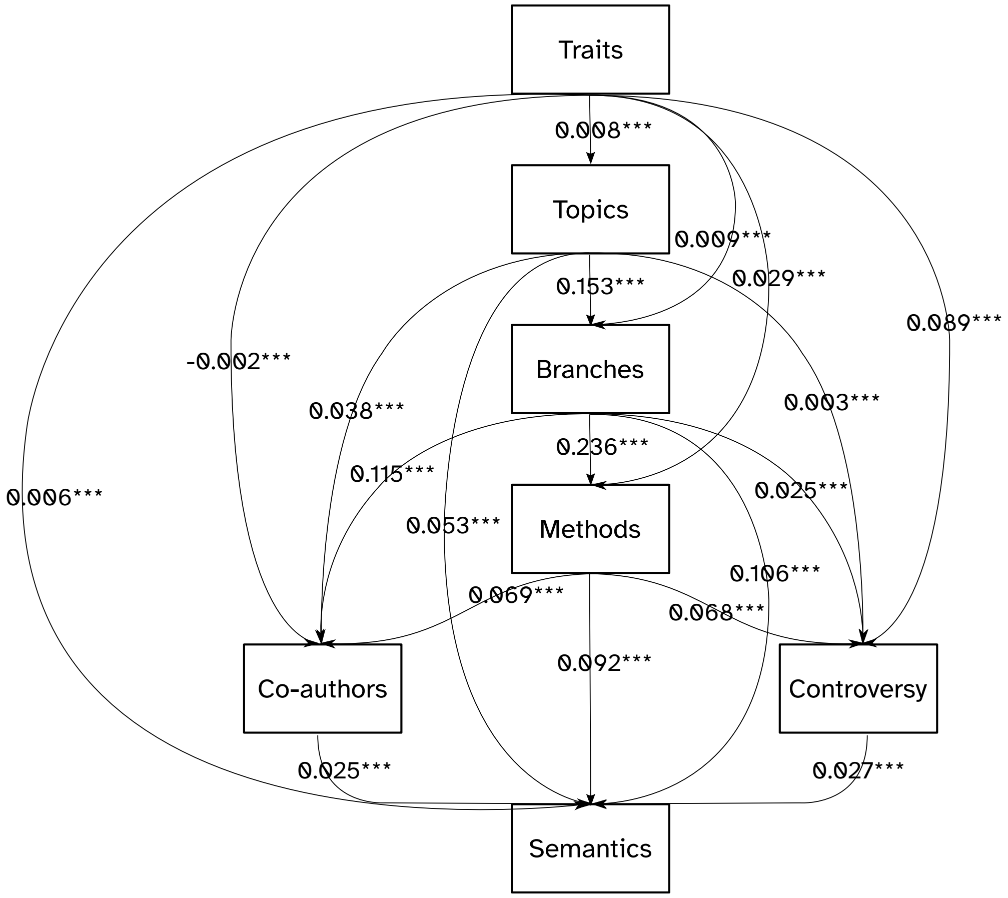

Pathways from the first SEM above (cf. Supplementary Table 8). * $p < 0.05$, ** $p < 0.01$, *** $p < 0.001$, all two-sided and based on z-statistics.

Supplementary Table 9: Structural Equation Model 2 Results

DV	IV	Estimate	Std. Err.	z-value	p-value	95% CI
topic	trait	0.008	< 0.001	35.271	< 0.001	[0.008, 0.009]
branch	trait	0.009	< 0.001	38.569	< 0.001	[0.008, 0.009]
branch	topic	0.153	< 0.001	795.646	< 0.001	[0.152, 0.153]
method	trait	0.027	< 0.001	129.963	< 0.001	[0.027, 0.028]
method	branch	0.233	< 0.001	1295.369	< 0.001	[0.233, 0.234]
coauthor	trait	-0.002	< 0.001	-8.166	< 0.001	[-0.002, -0.001]
coauthor	topic	0.036	< 0.001	203.939	< 0.001	[0.036, 0.036]
coauthor	branch	0.110	< 0.001	595.699	< 0.001	[0.109, 0.11]
coauthor	method	0.069	< 0.001	358.666	< 0.001	[0.069, 0.07]
controversy	trait	0.086	< 0.001	442.016	< 0.001	[0.086, 0.087]
controversy	topic	0.003	< 0.001	19.514	< 0.001	[0.003, 0.004]
controversy	branch	0.024	< 0.001	140.615	< 0.001	[0.024, 0.025]
controversy	method	0.070	< 0.001	385.947	< 0.001	[0.07, 0.07]
citations	trait	-0.010	< 0.001	-50.519	< 0.001	[-0.01, -0.009]
citations	topic	0.110	< 0.001	670.254	< 0.001	[0.11, 0.111]
citations	branch	0.290	< 0.001	1684.131	< 0.001	[0.29, 0.291]
citations	method	0.253	< 0.001	1400.533	< 0.001	[0.252, 0.253]
citations	coauthor	0.179	< 0.001	980.932	< 0.001	[0.179, 0.179]
citations	controversy	0.024	< 0.001	121.177	< 0.001	[0.023, 0.024]

Supplementary Figure 2: Structural Equation Model 2 Results

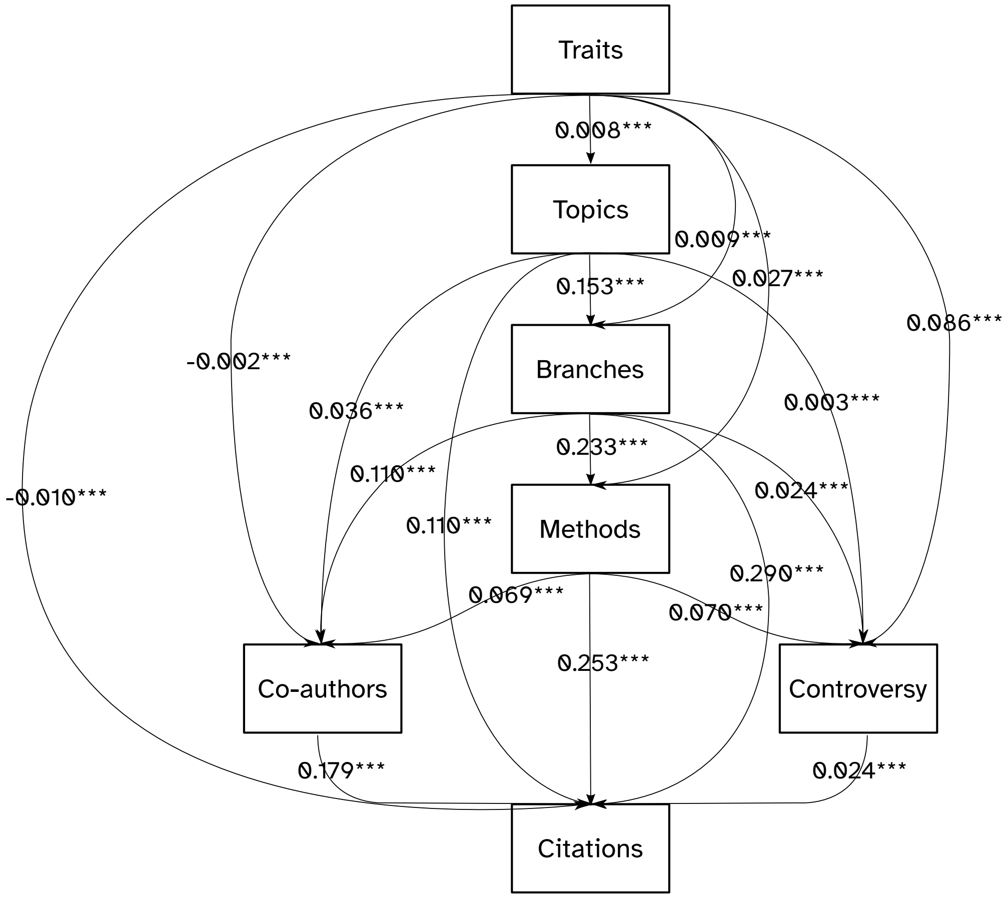

Pathways from the second SEM above (cf. Supplementary Table 9). * $p < 0.05$, ** $p < 0.01$, *** $p < 0.001$, all two-sided and based on z-statistics.

Appendix 5 Robustness checks

With a large-enough sample size, even random noise could pass conventional thresholds for significance. While we stress that we do not rely on a conventional $p < 0.05$ threshold anywhere in the main text (and indeed use much smaller Bonferroni-corrected thresholds such as $p < 0.000208$ in main text Figure 3a, $p < 0.000142$ in Figure 3b and $p < 0.000223$ in Figure 3c, all two-tailed), we ran additional analyses to assess the extent to which reported effects may have reflected the large sample size.

To do so, we drew random samples from our data for simulated sample sizes ranging from 300 to 3000 in multiples of 300. For each simulated sample size in this range, we repeatedly drew random samples and re-ran benchmark analyses on each of those samples, assessing the stability of effects across simulated sample sizes.

For the benchmarks, we focused on one of our main findings: the association between cognitive measure Tolerance for Ambiguity and numerous controversial themes (Figure 3c). We selected the largest effect in that column (Tolerance for Ambiguity∼Rational self-interest: $r = -0.18$), the smallest effect in that column that passed the Bonferroni-adjusted cutoff (Tolerance for Ambiguity∼Mind universal: $r = -0.07$), and an effect numerically intermediate between these extremes (Tolerance for Ambiguity∼Holistic view: $r = 0.12$).

We repeated the sampling procedure 1000 times for each simulated sample size, each time tracking the size and direction of the correlation coefficient for each of these benchmark associations, as well as whether it passed the conventional $p < 0.05$ threshold. Each panel in Supplementary Figure 3 focuses on one of these associations. The top half of each panel plots the median correlation coefficient across all 1000 simulations for each sample size, as well as indicating the central 95% of values (2.5% to 97.5%) with a ribbon. The bottom half of each panel plots how often the association was significant for each simulated sample size.

The larger $r = -0.18$ effect was remarkably stable across the whole range of simulated sample sizes, and the intermediate $r = 0.12$ effect lay above 0 in over 95% of samples even at the smallest simulated sample size, where we would be more likely to find a significant effect than not. Even for the smallest $r = -0.07$ effect, our sample size would have had to drop below 1000 (almost an eight of the actual size) before 0 lies within 95% of simulated effects, or for it to be more likely that we find a non-significant effect.

Thus, it is not the case that we are only finding such effects because we had almost 8000 respondents. In the Discussion, we further address why anything more than small effects would be implausible for this topic.

Supplementary Figure 3: Effect sizes across a range of simulated sample sizes for select controversial themes as a function of cognitive trait 'tolerance of ambiguity'.

a

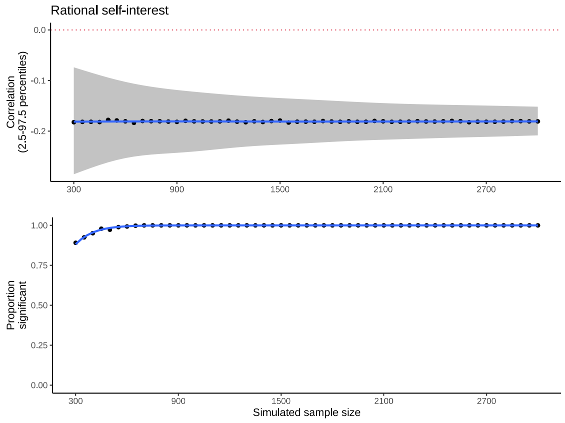

b

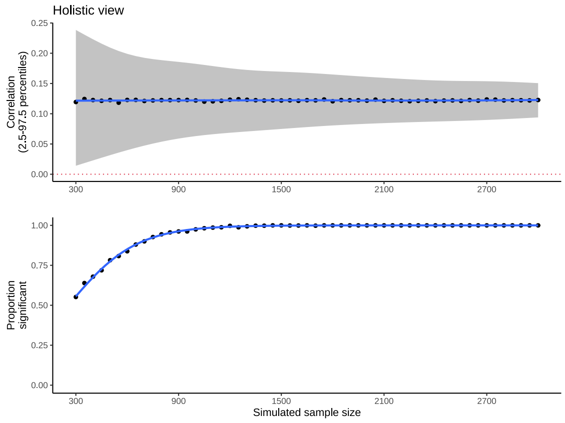

c

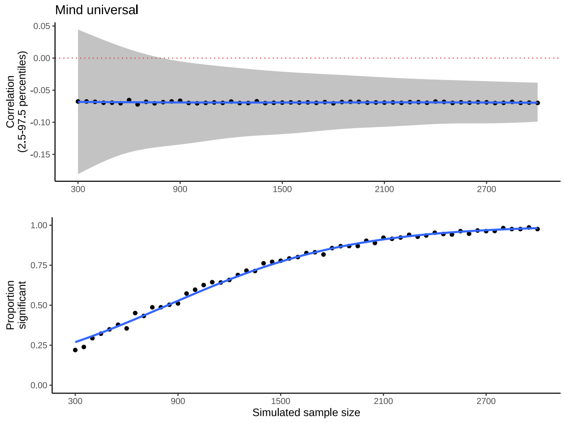

Plots of the effect sizes (top) and the proportion of samples with a significant effect (bottom) across associations between Tolerance for Ambiguity and three benchmark controversial themes: (a) Rational self-interest; (b) Holistic view; and (c) Mind universal. For the plots of effect size, the dots show the median across 1000 random samples for a given simulated sample size, the blue fit lines represent a (Loess) smooth of those medians, and the while the grey ribbons show the central 95% of the distribution. For the plots of significance, the dots simply show what proportion of the 1000 samples had a significant effect at the conventional $p < 0.05$ level, with the blue fit line reflecting a (Loess) smooth of those medians.

References

[1] Radim Řehůřek and Petr Sojka. Software Framework for Topic Modelling with Large Corpora. In *Proceedings of the LREC 2010 Workshop on New Challenges for NLP Frameworks*, pages 45–50, Valletta, Malta, May 2010. ELRA. `http://is.muni.cz/publication/884893/en`.

[2] Tomas Mikolov, Ilya Sutskever, Kai Chen, Greg Corrado, and Jeffrey Dean. Distributed representations of words and phrases and their compositionality. In *Proceedings of the 26th International Conference on Neural Information Processing Systems*, pages 3111–3119, 2013.

[3] Aditya Grover and Jure Leskovec. Node2vec: Scalable feature learning for networks. In *Proceedings of the 22nd ACM SIGKDD International Conference on Knowledge Discovery and Data Mining*, KDD '16, pages 855–864, New York, NY, USA, 2016. Association for Computing Machinery. ISBN 9781450342322. doi: 10.1145/2939672.2939754. URL `https://doi.org/10.1145/2939672.2939754`.
