# Supplementary material for: Differences in psychologists’ cognitive traits are associated with scientific divides
Source: Nat Hum Behav. 2025 Apr 17;9(6):1147–61. doi: 10.1038/s41562-025-02153-1 (PMC12185325; doi:10.1038/s41562-025-02153-1)
Supplement: Supplementary file 2 — Reporting Summary [file 41562_2025_2153_MOESM2_ESM.pdf]

nature portfolio

Corresponding author(s): Justin Sulik

Last updated by author(s): 10.10.2024

Reporting Summary

Nature Portfolio wishes to improve the reproducibility of the work that we publish. This form provides structure for consistency and transparency in reporting. For further information on Nature Portfolio policies, see our Editorial Policies and the Editorial Policy Checklist.

Statistics

For all statistical analyses, confirm that the following items are present in the figure legend, table legend, main text, or Methods section.

n/a	Confirmed	
☐	☒	The exact sample size (n) for each experimental group/condition, given as a discrete number and unit of measurement
☐	☒	A statement on whether measurements were taken from distinct samples or whether the same sample was measured repeatedly
☐	☒	The statistical test(s) used AND whether they are one- or two-sided<br>*Only common tests should be described solely by name; describe more complex techniques in the Methods section.*
☐	☒	A description of all covariates tested
☐	☒	A description of any assumptions or corrections, such as tests of normality and adjustment for multiple comparisons
☐	☒	A full description of the statistical parameters including central tendency (e.g. means) or other basic estimates (e.g. regression coefficient) AND variation (e.g. standard deviation) or associated estimates of uncertainty (e.g. confidence intervals)
☐	☒	For null hypothesis testing, the test statistic (e.g. F, t, r) with confidence intervals, effect sizes, degrees of freedom and P value noted<br>*Give P values as exact values whenever suitable.*
☐	☒	For Bayesian analysis, information on the choice of priors and Markov chain Monte Carlo settings
☐	☒	For hierarchical and complex designs, identification of the appropriate level for tests and full reporting of outcomes
☐	☒	Estimates of effect sizes (e.g. Cohen's d, Pearson's r), indicating how they were calculated

Our web collection on statistics for biologists contains articles on many of the points above.

Software and code

Policy information about availability of computer code

Data collection	Data were collected via the Qualtrics platform
Data analysis	Survey data were analyzed in R (4.4.1) with analysis code provided in the online repository at https://osf.io/zyec9. The analysis scripts list R libraries. Bibliometric models were created in Python (version 3.7.6) with Gensim (version 3.8.0) and node2vec (version 0.4.1, https://github.com/eliorc/node2vec)

For manuscripts utilizing custom algorithms or software that are central to the research but not yet described in published literature, software must be made available to editors and reviewers. We strongly encourage code deposition in a community repository (e.g. GitHub). See the Nature Portfolio guidelines for submitting code & software for further information.

Data

Policy information about availability of data

All manuscripts must include a data availability statement. This statement should provide the following information, where applicable:

- Accession codes, unique identifiers, or web links for publicly available datasets
- A description of any restrictions on data availability
- For clinical datasets or third party data, please ensure that the statement adheres to our policy

Survey data are available at https://osf.io/zyec9/. Web Of Science data used in participant recruitment and bibliometric models are not openly available, as Web of Science is a paid-access platform. The same applies to article abstracts retrieved from Microsoft Academic Graph (MAG).

Research involving human participants, their data, or biological material

Policy information about studies with human participants or human data. See also policy information about sex, gender (identity/presentation), and sexual orientation and race, ethnicity and racism.

Reporting on sex and gender	Participants self-reported gender. In addition to binary options (and an explicit opt-out response response option), participants could enter their own description of their gender identity.
Reporting on race, ethnicity, or other socially relevant groupings	N/A
Population characteristics	See above, and Supplementary Figure 1
Recruitment	Participants were recruited via email, based on email addresses published as corresponding addresses for academic research articles in psychology journals. The Methods and Limitations sections contain further details, including discussion of potential selection biases.
Ethics oversight	The study was approved by the University of Chicago Social & Behavioral Sciences IRB

Note that full information on the approval of the study protocol must also be provided in the manuscript.

Field-specific reporting

Please select the one below that is the best fit for your research. If you are not sure, read the appropriate sections before making your selection.

☐ Life sciences ☒ Behavioural & social sciences ☐ Ecological, evolutionary & environmental sciences

For a reference copy of the document with all sections, see nature.com/documents/nr-reporting-summary-flat.pdf

Behavioural & social sciences study design

All studies must disclose on these points even when the disclosure is negative.

Study description	Mixed methods: quantitative cross-sectional survey combined with bibliometric models of published research articles.
Research sample	Academic psychologists (and researchers in related disciplines) who had published research articles with a corresponding email address in journals classed as "psychology" by Web of Science. The final sample included 4182 men, 3683 women and 108 non-binary respondents. The modal decade of age was 30-39. Further participant information is provided in Supplementary Figure 1. The sample is not intended to be representative due to the recruitment strategy below, though the Limitations section discusses issues of representativeness.
Sampling strategy	Sampling was a mixture of convenience sampling (participants were emailed an invitation to the study based on email addresses we retrieved from academic psychology journals) and snowballing sampling (participants were invited to forward the invitation to their colleagues). The Limitations section addresses the adequacy of the sample size, as does Appendix 4 in the Supplementary Material.
Data collection	Data were collected online via the Qualtrics survey platform.
Timing	24.08.2016 - 30.08.2018
Data exclusions	No data were excluded from analysis
Non-participation	We retrieved 278,692 email addresses from psychology journals and sent invitations to all of these. 7973 (or 3%) completed the study. We report this in the "Limitations" section, comparing our 3% rate with response rates to other similar studies, and comparing respondents with non-respondents on bibliographic dimensions.
Randomization	Randomization not relevant to a cross-sectional survey

Reporting for specific materials, systems and methods

We require information from authors about some types of materials, experimental systems and methods used in many studies. Here, indicate whether each material, system or method listed is relevant to your study. If you are not sure if a list item applies to your research, read the appropriate section before selecting a response.

Materials & experimental systems

n/a	Involved in the study
☒	☐ Antibodies
☒	☐ Eukaryotic cell lines
☒	☐ Palaeontology and archaeology
☒	☐ Animals and other organisms
☒	☐ Clinical data
☒	☐ Dual use research of concern
☒	☐ Plants

Methods

n/a	Involved in the study
☒	☐ ChIP-seq
☒	☐ Flow cytometry
☒	☐ MRI-based neuroimaging

Plants

Seed stocks	NA
Novel plant genotypes	NA
Authentication	NA
